# Bioactive Bromotyrosine-Derived Alkaloids from the Polynesian Sponge *Suberea ianthelliformis*

**DOI:** 10.3390/md16050146

**Published:** 2018-04-27

**Authors:** Amr El-Demerdash, Céline Moriou, Jordan Toullec, Marc Besson, Stéphanie Soulet, Nelly Schmitt, Sylvain Petek, David Lecchini, Cécile Debitus, Ali Al-Mourabit

**Affiliations:** 1 Institut de Chimie des Substances Naturelles, CNRS UPR 2301, University Paris-Sud, University of Paris-Saclay, 1, Avenue de la Terrasse, 91198 Gif-Sur-Yvette, France; eldemerdash555@gmail.com (A.E.-D.); Celine.Moriou@cnrs.fr (C.M.); 2 Organic Chemistry Division, Chemistry Department, Faculty of Science, Mansoura University, Mansoura 35516, Egypt; 3 LEMAR, IRD, UBO, CNRS, IFREMER, IUEM, 29280 Plouzané, France; Jordan.Toullec@univ-brest.fr (J.T.); sylvain.petek@ird.fr (S.P.); 4 CRIOBE, CNRS, EPHE, UPVD, PSL Research University, 98729 Moorea, French Polynesia; bessonmarcluc@gmail.com (M.B.); david.lecchini@ephe.sorbonne.fr (D.L.); 5 Observatoire Océanologique de Banyuls-sur-Mer, Université Pierre et Marie Curie Paris, 66650 Banyuls-sur-Mer, France; 6 EIO, UPF, ILM, IFREMER, IRD, Faa’a, 98702 Tahiti, French Polynesia; stephanie.soulet@upf.pf (S.S.); nelly.schmitt@upf.pf (N.S.)

**Keywords:** brominated tyrosine alkaloids, *Suberea ianthelliformis*, cytotoxicity, acetylcholinesterase inhibition

## Abstract

Herein, we describe the isolation and spectroscopic identification of eight new tetrabrominated tyrosine alkaloids **2**–**9** from the Polynesian sponge *Suberea ianthelliformis*, along with known major compound psammaplysene D (**1**), *N*,*N*-dimethyldibromotyramine, 5-hydroxy xanthenuric acid, and xanthenuric acid. Cytotoxicity and acetylcholinesterase inhibition activities were evaluated for some of the isolated metabolites. They exhibited moderate antiproliferative activity against KB cancer cell lines, but psammaplysene D (**1**) displayed substantial cytotoxicity as well as acetylcholinesterase inhibition with IC_50_ values of 0.7 μM and 1.3 μM, respectively.

## 1. Introduction

**Figure 1:**
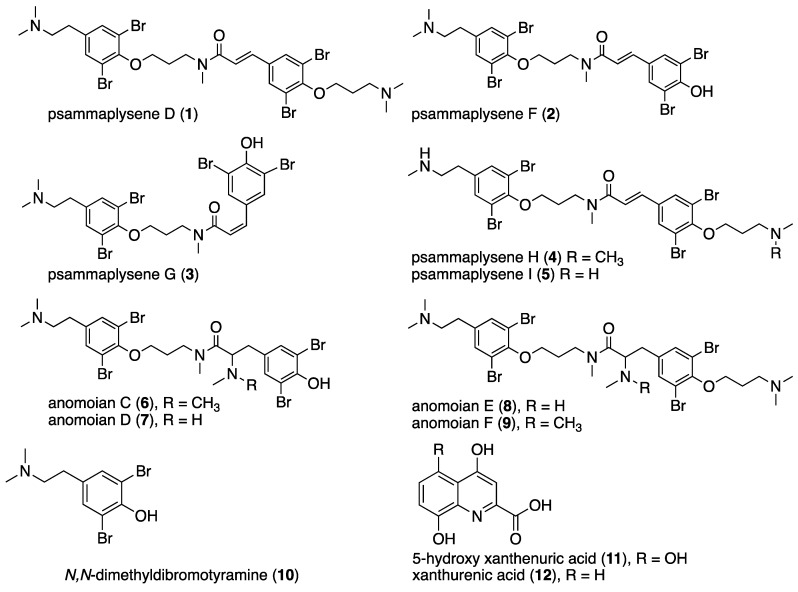
Structures of isolated compounds **1**–**12**.

Sponges of the marine genus *Suberea* (order: Verongiida) are known to produce a large array of structurally diverse brominated tyrosine alkaloids, which are considered as chemotaxonomical markers [1,2,3]. Nevertheless, bromotyrosine-derived metabolites have been isolated from sponges belonging to distinct orders such as Agelasida [4] and others. In addition, an interesting recent study showed that culture of the marine sponge-derived bacterium *Pseudovibrio denitrificans* Ab134 isolated from the *Arenosclera brasiliensis* sponge (order Haplosclerida) can produce bromotyrosine-derived alkaloids as well [5]. Although misidentifications and contaminations due to misplaced specimens are possible, these reports compromise this family of compounds as a Verongiida marker and suggest staying cautious about secondary metabolites markers for sponge phylogeny [6]. The bromotyrosine derivatives’ architectures vary from the simple monomeric molecules such as subereaphenol K [7], or the dimeric suberedamine A [8,9], to more complex derivatives like the well-known fistularin-3 [10]. Many compounds of this class of bromotyrosine alkaloids display a wide scope of bioactivities including cytotoxicity against murine leukemia L1201 cells and KB cells [8,9], DNA demethylating [11], antiproliferative [12], antimicrobial [13,14,15,16], and antiplasmodial activities [17]. In continuation of our search for bioactive marine natural products from South Pacific sponges [18], in both health and chemical ecology fields, we report here the isolation, structural determination, and cytotoxicity evaluation of eight new tetrabromotyrosine alkaloids (Figure 1), along with four known compounds from the sponge *Suberea ianthelliformis*. The known major compound psammaplysene D (**1**) was also evaluated for its acetylcholinesterase inhibition activity. Although acetylcholinesterase inhibition has been mostly explored as treatment for Alzheimer’s pathology, our attention has been brought to the chemical ecology importance of such enzyme inhibitors in their environment or against predation [19]. Only one publication relates fish toxicity on *Gambusia affini*s (western mosquitofish) and butylcholinesterase from a *Latrunculia magnifica* toxin [20]. We describe our observations of the inhibitory effect of psammaplysene D on acetylcholinesterase, using balneation experiments on two fish species, *Poecilia reticulata* (guppy) and *Acanthurus triostegus* (reef fish).

## 2. Results and Discussion

### 2.1. Isolation and Structure Elucidation

Each crude extract obtained by CH_2_Cl_2_ (10.5 g) and *n*-BuOH, (13 g) of *Suberea ianthelliformis* was subjected to normal phase MPLC and eluted with gradients of CH_2_Cl_2_/MeOH. The sub-fractions were purified by preparative, semi-preparative, and analytical reversed-phase HPLC and led to the isolation of eight new compounds **2**–**9**, along with the known derivatives psammaplysene D (**1**), *N*,*N*-dimethyldibromotyramine (**10**), 5-hydroxy xanthurenic acid (**11**) and xanthurenic acid acid (**12**) (Figure 1). The known natural products psammaplysene D (**1**) [21], *N*,*N*-dimethyldibromotyramine (**10**) [22], 5-hydroxy xanthenuric acid (4,5,8-trihydroxyquinoline-2-carboxilic acid) (**11**) [23] and xanthurenic acid (**12**) [24] were identified by comparison of their ^1^H NMR and MS data with those reported in the literature. It is worth to point out that psammaplysene D (**1**) was isolated by normal phase MPLC as the major compound (6.7 g) representing over 50% of the entire *n*-BuOH extract.

**Figure 2:**
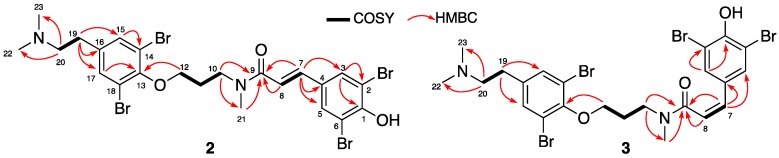
COSY and key HMBC correlations for psammaplysenes F (**2**) and G (**3**).

The molecular formula C_23_H_27_^79^Br_2_^81^Br_2_N_2_O_3_ of compound **2** was determined by HRESIMS (*m*/*z* 698.8675 [M + H]^+^) indicating ten degrees of unsaturation. The isotopic pattern of the protonated molecule confirmed the presence of four bromines. Analysis of the NMR data recorded for **2** revealed striking similarities with the NMR data recorded for the co-isolated psammaplysene D (**1**), except for the absence of the signal corresponding to the propylamine group, as confirmed by the HRESIMS spectrum. In general, the ^1^H NMR spectra of these families of compounds were complicated by the multiplicity of the signals due to the two rotational isomers *trans*/*cis* (6:4 ratio) along the amide bond [21]. The same behavior was already reported for tetrabromotyrosine derivatives psammaplysene A-D, isolated from the sponges *Psammaplysilla* and *Psammoclemma* [21,22,23,24,25]. The 1D and 2D NMR analysis revealed the existence of four aromatic protons comprising two singlets at δ_H_ 7.78 and 7.70, assigned to H-3/5, and another broad singlet at δ_H_ 7.57 integrating for 2 protons, attributed to H-15/17. An A-B system signal, corresponding to two olefinic protons at δ_H_ 7.39–7.36/7.38–7.35 (*J* = 15.5 Hz) and δ_H_ 7.15–7.11/7.02–6.99 (*J* = 15.5 Hz), were assigned to the *E* olefin –CH-7=CH-8–, satisfying the tenth degree of unsaturation. The HMBC data analysis showed correlations between H-7 and the two sp^2^ aromatic carbons C-3/5 (δ_C_ 133.09/133.01) and the sp^2^ non-protonated carbon C-4 (δ_C_ 130.98) confirming the connection of the *trans*-cinnamoyl motif to the 2,6-dibrominated phenolic ring (deduced with the chemical shifts of C-2/6 at δ_C_ 112.80 and C-1 at δ_C_ 154.10). The second olefinic proton H-8 exhibited an HMBC correlation with the non-protonated carbon C-9 at (δ_C_ 168.96/168.80) linking this end to an amidic carbonyl function. HSQC and COSY spectra analysis allowed the assignment of the signals at δ_H_ 4.11 and 4.05 (2H, 2t, *J* = 6.4), 3.89 and 3.74 (2H, 2t, *J* = 7.2 Hz), 2.22 and 2.16 (2H, 2m) to the three propylic carbon sequence *N*–CH_2_-10–CH_2_-11–CH_2_-12–O, while those at δ_H_ 3.30 (2H, m) and 3.00 (2H, m) were attributed to the ethylic chain CH_2_-19–CH_2_-20. The protons H-20 were correlated to *N*-Me_22_ and *N*-Me_23_ by HMBC analysis (Figure 2). The methylene group CH_2_-10 was connected to the amidic *N*-Me_21_ based on its chemical shift (δ_C_ 47.14/48.2) and the HMBC correlation to C-9 (δ_C_ 168.96/168.80) and C-21 (δ_C_ 34.74/36.55). The chemical shifts of CH_2_-12 at (δ_H_ 4.11/4.05) and δ_C_ (71.94/72.64) showed its link to the ether oxygen. Furthermore, the correlation of H-12 to the non-protonated carbon C-13 (δ_C_ 153.88/153.94) allowed the connection of this part of the molecule to the other 1,2,4,6-tetrasubstituted-phenyl ring. Furthermore, CH_2_-19 (δ_H_ 3.00) displayed HMBC correlations with the aromatic non-protonated carbon C-16 (δ_C_ 136.81/136.99) and CH-15/17 (134.63/134.56), confirming the substitution of this aromatic ring by the *N*,*N*-dimethylaminoethyl chain. Compound **2** was named psammaplysene F.

Compound **3** displayed the same molecular formula than compound **2** obtained by HRESIMS (*m*/*z* 698.8675 [M + H]^+^). A preliminary ^1^H NMR inspection (Table 1) showed similarity between compounds **1** and **2,** which was easily observed by superimposition of their spectra. The A-B system of two downfield doublets of doublets at δ_H_ 6.49–6.52/6.56–6.58 (*J* = 12.5 Hz, *cis*) and δ_H_ 6.13–6.16/6.09–6.12 (*J* = 12.5 Hz) were assigned to –CH-7=CH-8 of the cinnamoyl moiety with a *Z* configuration. Furthermore, the COSY, HSQC, and HMBC analysis data (Figure 2) showed the same correlations as previously found for **2**. Thus, the tetrabrominated tyrosine **3** was assigned to psammaplysene G.

**Figure 3:**
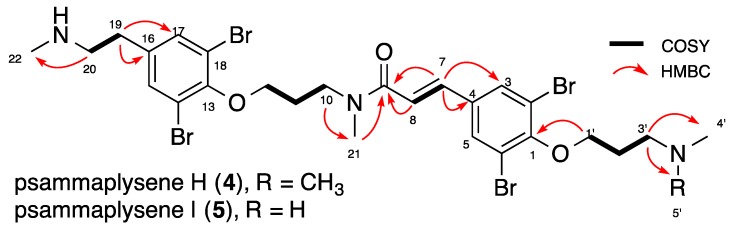
COSY and key HMBC correlations for psammaplysenes H (**4**) and I (**5**).

Compounds **4** and **5** were obtained in a minute quantity (0.7 mg) as a 1:1 mixture that could not be separated. Due to the minute quantity of the mixture, we decided to determine the structures of the latter two metabolites from the mixture. HRESIMS analysis revealed the molecular formulas C_27_H_36_^79^Br_2_^81^Br_2_N_3_O_3_ (*m*/*z* 769.9521 [M + H]^+^) and C_26_H_34_^79^Br_2_^81^Br_2_N_3_O_3_ (*m*/*z* 755.9309 [M + H]^+^) for **4** and **5**, with similar isotopic patterns indicating the presence of four bromines, but 14 and 28 a.m.u. (atomic mass unit) less than psammaplysene D (**1**), respectively. Direct comparison of the ^1^H NMR spectra of this mixture (Table 2) and the co-isolated major compound psammaplysene D (**1**) showed good superimposition, including the rotameric forms. The main differences concerned the signals of CH_2_-3′, CH_2_-19 and CH_2_-20 (Figure 3) indicating modifications of the number of the *N*-methyl groups linked to them. The 2D NMR experiments (COSY, HSQC, and HMBC) of both compounds showed the same correlations as the co-isolated psammaplysene D (**1**) (Figure 2). ^1^H NMR (Table 2) revealed four signals of *N*-methyl groups at δ_H_ 3.10 and 3.29 (2s, 6H, s-*trans*-CH_3_-21, s-*cis*-CH_3_-21), δ_H_ 2.70 (s, CH_3_-22, 3H) and δ_H_ 2.74–2.79 (CH_3_-4′ and CH_3_-5′, 6H). The HMBC analysis showed correlation from *N*-CH_3_-22 (δ_H_ 2.70, δ_C_ 34.0) to C-20 (δ_C_ 51.1) for compound **4**. The chemical shifts differences observed for H-19 (δ_H_ 2.93), H-20 (δ_H_ 3.21) and CH_3_-22 (δ_H_ 2.70) suggested a modification on this part of the molecule. The chemical shifts of C-20 (δ_C_ 51.1) and CH_3_-22 (δ_C_ 34.0) suggested the existence of only one methyl group on this side of the chain. At the other end of the molecule, correlations of *N*-CH_3_-4′ and 5′ (δ_H_ 2.74–2.79, δ_C_ 44.7) to C-3′ (δ_C_ 57.2) along with the chemical shift of C-3′, showed the presence of two methyl groups on the nitrogen *N*-C-3′. The chemical shift of C-20 and C-22 were the same for compound **5**, but the chemical shift of C-2′ (δ_C_ 27.9), C-3′ (δ_C_ 48.7), and CH_3_-4′ (δ_C_ 34.0), as well as the HMBC correlations between C-3′ and CH_3_-4′, suggested the presence of a NH-Me connected to the carbon C-3′. Therefore, our mixture was composed of two psammaplysene D derivatives lacking one and two *N*-methyls and named psammaplysene H (**4**) and psammaplysene I (**5**), respectively.

**Figure 4:**
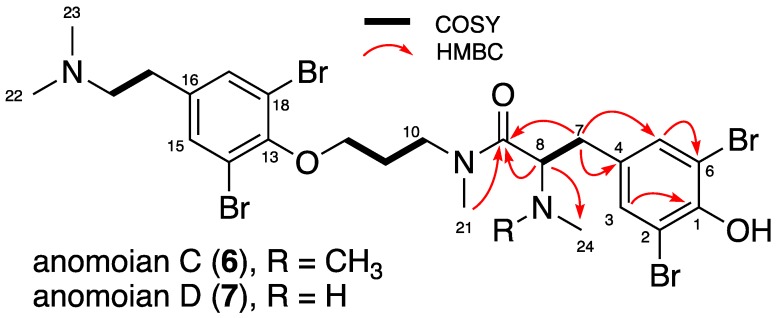
COSY and key HMBC correlations for anomoians C (**6**) and D (**7**).

Compounds 6 and 7 exhibited the molecular formula were determined by HRESIMS as C_25_H_33_^79^Br_2_^81^Br_2_N_3_O_3_ at *m*/*z* 743.9386 [M + H]^+^, and C_24_H_31_^79^Br_2_^81^Br_2_N_3_O_3_ at *m/z* 729.9190 [M + H]^+^ respectively, indicating nine degrees of unsaturation with an isotopic pattern of four bromines. These NMR and mass data showed that compound 7 has one methyl group less than compound 6. Examination of the ^1^H NMR and HSQC spectra (Table 3) displayed signals with similar chemical shifts to those reported for the tetrabromotyrosine compounds anomoians A and B, previously isolated from *Anomoianthella popeae* [26] (order: Verongiida) and from an unknown species of the genus *Hexadella* (order: Verongiida) collected in Indonesia [27]. 2D NMR analysis including COSY, HSQC, and HMBC (Table 3, Figure 4), allowed identification of the structures of 6 and 7 as the *O*-demethyl anomoian B and *O*-demethyl anomoian A, respectively. Thus, the new compounds were named anomoian C (6) and anomoian D (7). The ^1^H NMR signals of the aromatic protons CH-3/5 and CH-15/17 of these new compounds indicated a 6:4 rotameric proportionality.

**Figure 5:**
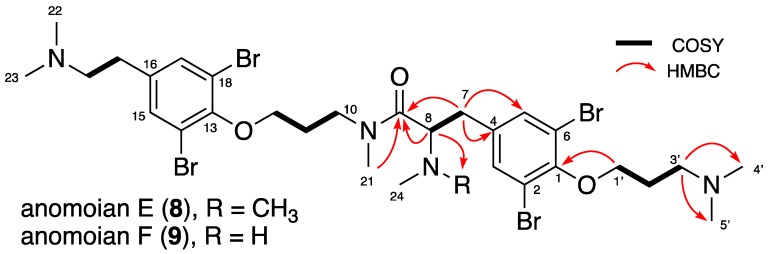
COSY and key HMBC correlations for anomoians E (**8**) and F (**9**).

Compound **8** had a protonated molecular ion at *m*/*z* 815.0068 [M + H]^+^ corresponding to the molecular formula C_29_H_43_^79^Br_2_^81^Br_2_N_4_O_3_, determined by HRESIMS. The mass spectrum indicated an isotopic matrix of a tetrabrominated compound. Quick examination of the ^1^H NMR spectrum (Table 4) showed signals with identical chemical shifts to those reported for anomoian C (**6**) and D (**7**). Furthermore, it revealed the presence of an additional dimethylaminopropylic chain, as observed for psammaplysene D (**1**). This was confirmed by the 2D NMR spectra analysis, including COSY, HSQC, and HMBC, enabling us to set the correlations shown in Figure 5. The new compound **8** was named anomoian E.

The HRESIMS data of 9 revealed the molecular formula C_30_H_45_^79^Br_2_^81^Br_2_N_4_O_3_ (*m*/*z* 829.0259 [M + H]^+^). Its ^1^H NMR spectrum was very similar to anomoian E (8) spectrum, except for the two additional methyl groups at δ_H_ 2.51/2.54 integrating for six protons for 9. HSQC, COSY, and HMBC spectra analysis led to the same correlations as in compound 8 and enabled the assignment of the *N*-8-methyl groups through an additional correlation from the *N*–CH_3_-24/25 (δ_H_ 2.51/2.54, 6H) to the methine group CH-8 (δ_H_ 3.97/3.89, m; δ_C_ 66.79/66.58). Therefore, structure 9 was determined as anomoian F, existing as 6:4 rotamers (Table 4, Figure 5).

The absolute configuration of the stereocenter C-8 for anomoian C-F (**6**–**9**) can be proposed by comparison of the values and signs of their optical rotations in MeOH ([α]${}_{D}^{25}$ +9°, +11°, +7° and +6°, respectively), with the closely related congeners, anomoian A ([α]_D_ +5.1°) [26], anomoian B ([α]_D_ +10.5°) [27], *iso*-Anomoian A ([α]${}_{D}^{20}$ +4.5°) [28], suberedamines A ([α]${}_{D}^{25}$ +21°) and B ([α]${}_{D}^{25}$ +16°) [9], indicating that all are bearing l-tyrosine residue. The synthetic *iso*-Anomoian A ([α]${}_{D}^{20}$ +4.5°) and suberedamines A ([α]${}_{D}^{20}$ +19.5°) allowed the determination of their absolute configurations [28].

### 2.2. Biological Activities

The isolated compounds **1**–**3** and **6**–**11** were tested for their cytotoxic activity against human epidermoid carcinoma cells (KB) cancer cell lines (Table 5) and acetylcholinesterase. The results showed of the compounds exhibited moderate cytotoxicity. The major compound psammaplysene D (**1**) showed the most interesting cytotoxicity with an IC_50_ = 0.7 μM. Anomoian E (**8**) and F (**9**), containing the *N*,*N*-dimethylaminopropyl side as psammaplysene D, but lacking the double bond –CH-7=CH-8, displayed a lower activity. Moreover, psammaplysene F (**2**), which bear a *trans*-cinnamoyl part, was found to exhibit a lower activity than psammaplysene D (**1**). Therefore, from a structural point of view, both motifs (*N*,*N*-dimethylaminopropyl and *trans*-cinnamoyl) seem to be together correlated to the activity of psammaplysene D (**1**).

The screening of our French Polynesian sponges extract library was performed on acetylcholinesterase inhibitor (AChE) in the aim to investigate a possible chemical defense of sessile organisms on the reef against fishes, their main predators. The chemical study of the bioactive extract of *Suberea ianthelliformis* using AChE led to the identification of psammaplysene D (**1**) as its unique in vitro AChE inhibitor with an IC_50_ = 1.3 μM. None of the other closely related compounds displayed any activity on this target enhancing here again the importance of the phenoxy-1-*N*,*N*-dimethyl propane chain and the *trans*-cinnamoyl motifs (Figure 1). Further kinetics investigation of the mode of AChE inhibition of psammaplysene D (convergence of the lines on the Lineweaver Burk plot, decreasing *V*_max_ values (from 0.0036 to 0.0007 μmol·min^−1^) and increasing *K*_M_ values (from 0.14 to 0.23 μM)) suggested a mixed competitive/non-competitive inhibition suggesting that psammaplysene D (**1**) not only binds to the free enzyme, but also to the enzyme-substrate complex. In order to try to evaluate the effect of psammaplysene D in the environment, it was further tested on fresh water fish (guppy, *Poecilia reticulata*) and reef fish (*Acanthurus triostegus*) feeding experiment using bathing with or without the bioactive compound. Guppies are fed with commercial flakes, but the wild reef fish feed on natural coral heads, and their appetite evaluated by counting the bites on the corals. Two experiments were run on guppies using *Suberea ianthelliformis* crude extract and purified psammaplysene D (**1**): an acute toxicity bioassay (100 μg/mL of crude extract or purified psammaplysene D (**1**), 1 h) and a chronic toxicity (5 μg/mL of crude extract or 1 μg/mL of purified psammalysene D (**1**), 72 h).

**Figure 6:**
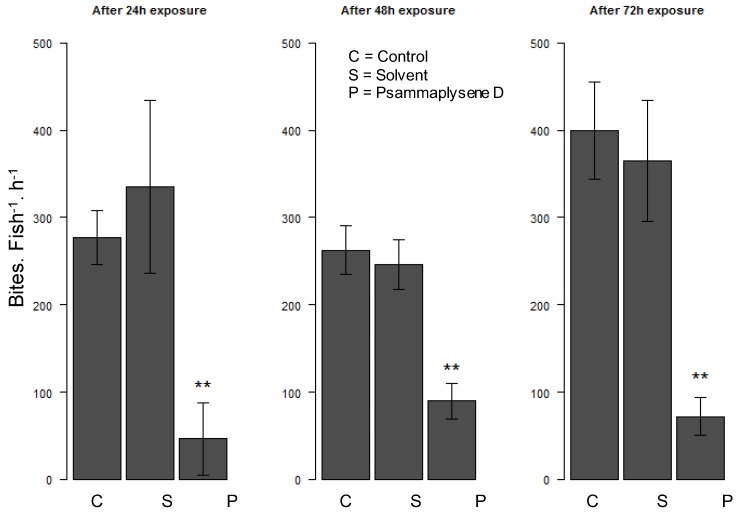
Number of bites on coral pieces per *A. triostegus* recruit and per hour. Error bars represent standard deviation of the mean (*N* = 6). ** *p* < 0.01.

During the acute exposure, fish were totally confused and had uncontrolled mobility, displaying sudden jumps and balance control loss, as soon as 20 min after the addition of psammaplysene D in the tank. This result can be compared to the observation reported by Nèeman et al. with *Latrunculia* species toxins [20]. Exposure of guppies during 24 h of at a sub-lethal dose (5 μg/mL of crude extract or 1 μg/mL of purified psammalysene D (**1**) of the compound caused passivity of the fish towards the food flakes offered daily, whereas those of the controls ate normally. These observations indicate a clear link between the loss of appetite and psammaplysene D (**1**) toxicity. Similar activity was observed on reef fish recruits (fish larvae undergoing metamorphosis) of *Acanthurus triostegus*: the presence psammaplysene D (**1**) (1 μg/mL) in tanks significantly and readily decreased the number of bites of recruits, suggesting a rapid saturation of the environment perception sensors of the fish larvae during the 72 h of experiment (Figure 6).

**Figure 7:**
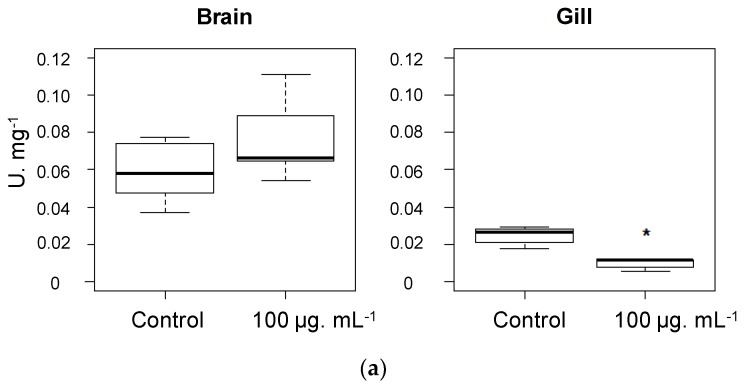

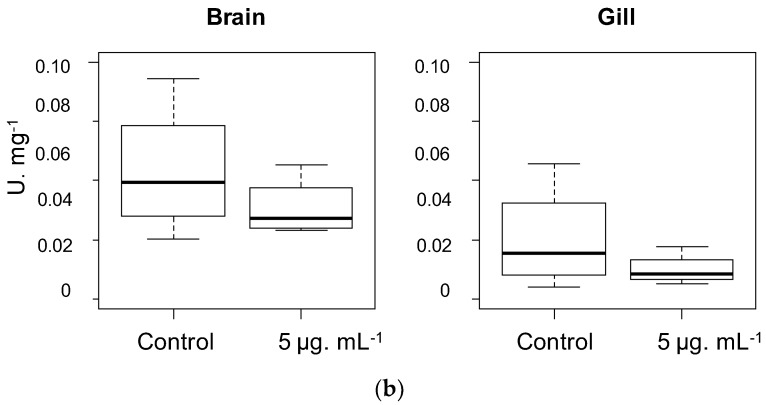
(**a**) AChE activity, U·mg^−1^, after 1 h exposure to 100 µg/mL of Psammalysene D (**1**). Boxplot distribution was made with 5 fish (*N* = 5). * *p* < 0.05; (**b**) AChE activity, U·mg^−1^, after 72 h exposure to 5 µg/mL of crude extract. Boxplot distribution was made with 5 fish (*N* = 5).

Psammaplysene D (**1**) displayed clearly behavioral effects on both guppies and reef fish larvae: they showed a loss of appetite from the first day of experiment. Reef fish juveniles’ behavior did not seem to be impacted by psammaplysene D. It is interesting to note that only *A. triostegus* larvae were impacted by the pure psammaplysene D, suggesting a change of sensibility towards the compound during the metamorphosis. The toxicity test at a high dose (100 μg/mL of crude extract or purified psammaplysene D) on the guppies led to their death after one hour bathing with either the crude extract or the purified psammaplysene D fraction (acute toxicity experiment). Brains and gills of these guppies were extracted and the AChE activity measured on the extracts (Figure 7a,b) [29]. The results were clearer with the purified psammaplysene D, showing a mild but significant decrease of the activity of the enzyme in the gills, suggesting an action on the olfactory organs (peripheral nervous system) that could not be further investigated there. A great number of AChE inhibitors targeting Alzheimer’s disease have been isolated from natural sources [30]. Among them, 6 compounds with activities ranging from IC_50_ 1 to 100 μM were isolated from Verongiida sponges (Table 6). It would be interesting also to further investigate those compounds as chemical anti-predatory defenses through fish antifeedant activity, since the fish model is easier to set up than related bioassays involving sponge predators, such as nudibranchs or crustaceans.

A common side effect of AChE inhibition treatments is anorexia [34,35], which strengthens the importance of natural AChE inhibitors as potential deterrents of predators such as fishes. Furthermore, the olfactory dysfunction in Alzheimer’s disease has also been demonstrated [36]. Psammaplysene D is the major compound of the sponge (0.66% *w*/*w*). It has been shown that tyrosine derived compounds are located in sphaerus cells of the sponge, allowing their release in the aquifer channels and further in the close environment of the sponge [37]. It would be thus interesting to track and quantify the concentration of this compound in the close reef environmental water of the sponges, using for example, solid phase adsorption toxin tracking (SPATT) [38], to allow the on field deterrent or reef protective activity of this compound acting softly on olfactory sense of predators in the marine environment. The importance of AChE inhibitors in the marine environment can be included in neuroecology of chemical defenses, a merging field of chemical ecology, which displays the importance of the links between ecology and evolution, secondary metabolites, behavior, and neurosciences [39].

## 3. Material and Methods

### 3.1. General Procedures

Optical rotations were measured using MCP-300 polarimeter (Anton Paar, Les Ulis, France). IR spectra were recorded on a BX FT-IR spectrometer (Perkin Elmer, Courtaboeuf, France). 1D and 2D NMR spectra were recorded on a Avance 500 and 600 MHz (CNRS-ICSN, Bruker, Wissembourg, France). The chemical shifts are relative to the residual signal solvent (CD_3_OD: δ_H_ 3.31; δ_C_ 49.20; DMF-*d_7_*: δ_H_ 8.03, 2.92, 2.75; δ_C_ 163.2, 34.9, 29.8). High-resolution mass spectra were obtained on a LCT Premier XE spectrometer (Waters, Guyancourt, France) in electrospray ionization mode by direct infusion of the purified compounds. Preparative HPLC was performed using Auto Prep system (Waters 600 controller and Waters 600 pump, equipped with a 996 Photodiode Array Detector, Waters, Guyancourt, France). A Varioskan^®^ Flash microplate reader (Thermo Scientific, Villebon-sur-Yvette, France) was used for the acetylcholinesterase experiments.

### 3.2. Animal Material

The sponge was collected off the coast of Nuku Hiva (8°55′977′ S–140°01′17′ W) between 5 and 42 m deep using SCUBA on the 28 August 2009 [40]. It was identified as *Suberea ianthelliformis* and a reference specimen is deposited at the Queensland Museum (Brisbane, Australia) under the accessing number G331075.

### 3.3. Isolation of Bioactive COMPOUNDS

The freeze-dried sponge sample (960 g) of *Suberea ianthelliformis* was extracted three times at room temperature with a 1:1 mixture of CH_2_Cl_2_/MeOH (1.5 L). The extracts were combined and dried under reduced pressure to afford a brown residue (50 g). Forty-five grams of this residue were partitioned between CH_2_Cl_2_ and H_2_O. The resulting organic layer was collected and dried under reduced pressure to afford a brown material (10.5 g). The aqueous layer was further extracted with *n*-BuOH to afford the butanolic extract (13 g). The remaining aqueous fraction was dried to afford 21.2 g material. The CH_2_Cl_2_ extract was then subjected to normal phase silica-gel MPLC. A gradient composed of CH_2_Cl_2_/MeOH (1:0 to 0:1), followed by a mixture of EtOAc/Acetone/H_2_O/Formic Acid (5/3/0.5/0.5) were used to afford 8 fractions F1 to F8. The *n*-BuOH extract was also subjected to normal phase silica gel MPLC, using the same gradient as before to afford 6 fractions F1 to F6. Promising fractions were selected based on the LC-MS analytical profile, and then each fraction was submitted for purification by preparative reversed phase HPLC (column: Waters Sunfire C_18_ 19 mm × 150 mm, 5 μ, H_2_O + 0.1% formic acid/CH_3_CN + 0.1% formic acid). Some sub-fractions were further purified using semi-preparative reversed phase column (Waters Sunfire C_18_, 10 mm × 150 mm, 5 μ, H_2_O + 0.1% formic acid/CH_3_CN + 0.1% formic acid).

*Psammaplysene D* (**1**): brown oil, (2.6 g); UV (MeOH) λ_max_ (log *ε*) 241 (1.30), 279 (2.90) nm; IR (neat) ν_max_ 2943, 1649, 1598, 1455, 1379, 738 cm^−1^; ^1^H and ^13^C NMR data, *Supporting Materials*; HRESIMS *m*/*z* 783.9673 [M + H]^+^ (calcd for C_28_H_38_N_3_O_3_Br_4_, 783.9606).

*Psammaplysene F* (**2**): pale yellow oil, (18.4 mg); UV (MeOH) λ_max_ (log *ε*) 208 (0.84), 270 (0.20) nm; IR (neat) ν_max_ 3381, 2941, 1636, 1596, 1471, 1456, 1399, 1257, 738 cm^−1^; ^1^H and ^13^C NMR data, Table 1; HRESIMS *m*/*z* 698.8675 [M + H]^+^ (calcd for C_23_H_27_N_2_O_3_^79^Br_2_^81^Br_2_, 698.8714).

*Psammaplysene G* (**3**): white powder, (8.1 mg); UV (MeOH) λ_max_ (log *ε*) 208 (0.24), 270 (0.08) nm; IR (neat) ν_max_ 2938, 1600, 1457, 1398, 1258, 738 cm^−1^; ^1^H and ^13^C NMR data, Table 1; HRESIMS *m*/*z* 698.8723 [M + H]^+^ (calcd for C_23_H_27_N_2_O_3_^79^Br_2_^81^Br_2_, 698.8714).

*Psammaplysene H* (**4**)/*Psammaplysene I* (**5**) mixture: pale yellow oil, (0.7 mg); ^1^H and ^13^C NMR data, Table 2; HRESIMS *m*/*z* 769.9521 [M + H]^+^ (calcd. for C_27_H_36_^79^Br_2_^81^Br_2_N_3_O_3_, 769.9449)/HRESIMS *m*/*z* 755.9309 [M + H]^+^ (calcd. for C_26_H_34_^79^Br_2_^81^Br_2_N_3_O_3_, 755.9293).

*Anomoian C* (**6**): colorless oil, (1.9 mg); [α]${}_{D}^{25}$ +9 (*c* 0.19, MeOH, as formate salt); UV (MeOH) λ_max_ (log *ε*) 211 (0.80), 283 (0.10) nm; IR (neat) ν_max_ 3396, 2938, 1635, 1458, 1258, 1039, 737 cm^−1^; ^1^H and ^13^C NMR data, Table 3; HRESIMS *m*/*z* 743.9386 [M + H]^+^ (calcd for C_25_H_34_N_3_O_3_^79^Br_2_^81^Br_2_, 743.9293).

*Anomoian D* (**7**): pale yellow oil, (1.2 mg); [α]${}_{D}^{25}$ +11 (*c* 0.12, MeOH, as formate salt); UV (MeOH) λ_max_ (log *ε*) 227 (2.00), 283 (0.40) nm; IR (neat) ν_max_ 3356, 2934, 1600, 1598, 1458, 1259, 1041, 738 cm^−1^; ^1^H and ^13^C NMR data, Table 3; HRESIMS *m*/*z* 729.9190 [M + H]^+^ (calcd for C_24_H_32_N_3_O_3_^79^Br_2_^81^Br_2_, 729.9136).

*Anomoian E* (**8**): colorless oil, (3.8 mg); [α]${}_{D}^{25}$ +7 (*c* 0.23, MeOH, as formate salt); UV (MeOH) λ_max_ (log *ε*) 222 (1.60), 276 (0.20) nm; IR (neat) ν_max_ 3382, 2937, 2782, 1635, 1457, 1257, 1040, 738 cm^−1^; ^1^H and ^13^C NMR data, Table 4; HRESIMS *m*/*z* 815.0068 [M + H]^+^ (calcd for C_29_H_43_N_4_O_3_^79^Br_2_^81^Br_2_, 815.0028).

*Anomoian F* (**9**): yellow oil, (3.8 mg); [α]${}_{D}^{25}$ +6 (*c* 0.38, MeOH, as formate salt); UV (MeOH) λ_max_ (log *ε*) 220 (1.30), 277 (0.20) nm; IR (neat) ν_max_ 3402, 2940, 2780, 1641, 1460, 1258, 1041, 737 cm^−1^; ^1^H and ^13^C NMR data, Table 4; HRESIMS *m*/*z* 829.0259 [M + H]^+^ (calcd for C_30_H_45_N_4_O_3_^79^Br_2_^81^Br_2_, 829.0184).

*N, N-Dimethyldibromotyramine* (**10**): yellow oil, (10.5 mg); UV (MeOH) λ_max_ (log *ε*) 222 (1.50), 291 (0.60) nm; IR (neat) ν_max_ 3255, 2957, 1636, 1596, 1283, 737 cm^−1^; ^1^H and ^13^C NMR data, Supplementary Materials; HRESIMS *m*/*z* 323.9388 [M + H]^+^ (calcd for C_10_H_14_NO^79^Br^81^Br, 323.9422).

*5-Hydroxy xanthenuric acid* (**11**): reddish brown amorphous material, (3.2 mg); UV (MeOH) λ_max_ (log *ε*) 210 (0.55), 236 (0.8), 336 (0.20) nm; IR (neat) ν_max_ 3360, 3250, 2980, 1726, 1644, 1590, 1447, 1246, 1051, 757, 683 cm^−1^; ^1^H and ^13^C NMR data, Supplementary Materials; HRESIMS *m*/*z* 222.0441 [M + H]^+^ (calcd for C_10_H_8_NO_5_, 222.0402).

*Xanthurenic acid (***12**): pale green oil, (1.5 mg); UV (MeOH) λ_max_ (log *ε*) 220 (0.35), 248 (0.60), 345 (0.15) nm; ^1^H and ^13^C NMR data, Supplementary Materials; HRESIMS *m*/*z* 206.0511 [M + H]^+^ (calcd for C_10_H_8_NO_4_, 206.0453).

### 3.4. Cell Culture and Cell Proliferation Assay

The human cell line KB originated from the National Cancer Institute (NCI) and was grown in Dulbecco’s Modified Eagle Medium (D-MEM) medium supplemented with 10% foetal calf serum, in the presence of penicillin, streptomycin, and fungizone in a 75 mL flask under 5% CO_2_. A total of 600 cells were plated in 96-well tissue culture plates in 200 L of medium and treated 24 h later with 2 μL of stock solution of compounds dissolved in DMSO using a Biomek 3000 (Beckman-Coulter, Brea, CA, USA). Controls received the same volume of DMSO (1% final volume). After 72 h exposure, cell titer 96 Aqueous One solution (Promega, Madison, WI, USA) was added and incubated for 3 h at 37 °C: the absorbance was monitored at 490 nm, and results were expressed as the inhibition of cell proliferation calculated as the ratio [(1 − (OD490 treated/OD490 control)) × 100] in triplicate experiments.

### 3.5. Acetylcholinesterase Inhibition Assay

The crude extract of the sponge and the purified psammaplysene D (**1**) were dissolved in DMSO at 10 mg/mL, stored at −20 °C and diluted subsequently 100 times in H_2_O prior testing. Determination of AChE-inhibitory activity was measured according the Ellman’s colorimetric method [41], in 96-well tissue culture plates. All reagents, buffers and salts were purchased from Sigma-Aldrich (Saint-Louis, MO, USA). Galantamine was used as a positive control for each analysis. Solvent controls received the same volume of water. After a 30 min incubation at 25 °C in the dark, absorbance was monitored at *λ* = 405 nm. Results were expressed as the percentage of enzyme inhibition calculated as the ratio: 100 × (OD_control_ − OD_treated_)/(OD_control_ − OD_blank_), where the blanks are the wells without AChE. Serial dilutions of the compound or extract were prepared (100, 50, 25, 12.5, 6.25, 3.12, 1.56, and 0.78 μg/mL) and tested according to the same procedure using 10 μL of each solution. IC_50_ values were graphically determined by plotting the percentage of inhibition against the compound concentrations. Each assay was performed in triplicate and results are expressed as mean values ± S.D.

#### 3.5.1. Kinetic Study

The inhibition mode of psammaplysene D on AChE was determined while measuring the enzyme activity in presence of an increasing concentration of ATCI (0.024, 0.049, 0.098, 0.19, 0.39, 0.78, 1.57, and 3.15 μM), in absence or presence of 2 μg/mL final concentration of purified psammaplysene D. The inhibition mode, V_max_, and K_M_ values were determined by double-reciprocal Lineweaver and Burk plot analysis (1934) of the data obtained.

#### 3.5.2. Guppy Bioassay

5 to 8 cm long guppies (*Poecilia reticulata*) were collected at night in a private fresh water pool at Papara (Tahiti, French Polynesia) and immediately acclimated at the laboratory temperature (25 °C). After acclimatization, fishes were parted in two 2 L tanks: one for the control group treated with solvent (*N* = 5). Another experiment was performed using a 1 h treatment with 100 μg/mL of crude extract of the sponge or purified psammaplysene D (**1**) to check their acute toxicity.

#### 3.5.3. Reef Fish Feeding Behavior Experiment

Manini (*Acanthurus triostegus*) were caught during new moon nights on the foreshore puddles at Tema’e public beach [42]. Recruits (fish larvae undergoing metamorphosis) and juveniles (larvae after the recruits stage) were collected and immediately used for the experiments. Natural lagoon sea-water was used and UV-sterilized prior use. Three conditions were tested on three groups: control, solvent, 1 μg/mL sponge extract treatment, with 4 or 5 fishes in each 3 L tank. The fishes were exposed under semi-static system for 72 h, the experimental solutions were renewed every 24 h to maintain clean experimental conditions. The experiments were performed with fish recruits and juveniles. Rubbles were disposed in each aquarium 1 h/day during 3 days. Feeding behavior experiment consists in counting the overall number of bites on those rubbles in each aquarium. Six videos sequences (*N* = 6) of 5 or 10 min per aquarium and per day were analyzed. Results are expressed in number of bites per fish and per hour.

#### 3.5.4. Statistical Analysis

All the values of AChE activity are represented in a boxplot distribution, because of non-normality of variable distribution (*N* = 4 or 5). The significant differences between controls and treatments (*p*-value *p* < 0.05) were analyzed with non-parametric test of Wilcoxon Mann Whitney. In feeding deterrent assay, pairs significant differences between different conditions (*p* < 0.05) were tested with non-parametric Kruskal Wallis test. All statistical assays were executed using R software (R-3.2.0, The R Foundation for Statistical Computing, Vienna, Austria).

## 4. Conclusions

This paper describes the isolation of eight new bromotyrosine secondary metabolites from the Polynesian Sponge *Suberea ianthelliformis*. Their structures are close to those of psammaplysenes and anomoians but all displaying methylated amidic nitrogen and rotameric mixtures. Since flexible syntheses were developed for psammaplysene A [43], and *iso*-anomoian A [28] and other analogues, our molecules present new derivatives for further optimization of biologically interesting molecules. They all exhibited moderate antiproliferative activity against KB cancer cell lines, but psammaplysene D (**1**) displayed substantial cytotoxicity as well as acetylcholinesterase inhibition with IC_50_ values of 0.7 μM and 1.3 μM, respectively. It would be interesting to further investigate the ecological role of the major psammaplysene D as chemical anti-predatory defenses through fish antifeedant activity. The available synthetic strategies would allow the preparation of our metabolites and a rage of their analogues for the screening of the inhibition of various cancer cell lines and SAR studies.

## Figures and Tables

**Table 1:** ^1^H NMR (500 MHz) and ^13^C NMR (125 MHz) data for Psammaplysenes F (**2**) and G (**3**) in CD_3_OD.

Position	Psammaplysene F (2)			Psammaplysene G (3)		
No.	*δ*_C_, Type ^a^	*δ*_H_ mult, (*J* in Hz) ^a^	HMBC	*δ*_C_, Type ^a^	*δ*_H_ mult, (*J* in Hz) ^a^	HMBC
1	154.10, C	-	-	153.29/153.04, C	-	-
2	112.80, C	-	-	112.49, C	-	-
3	133.09/133.01, CH	7.78/7.70, s	1, 2, 5, 6, 7	133.70/133.42, CH	7.53/7.54, s	1, 2, 5, 6, 7
4	130.98, C	-	-	130.70/131.16, C	-	-
5	133.09/133.01, CH	7.78/7.70, s	1, 2, 3, 6, 7	133.70/133.42, CH	7.53/7.54, s	1, 2, 3, 6, 7
6	112.80, C	-	-	112.49, C	-	-
7	140.89/141.19, CH	7.36–7.39/7.35–7.38, d (15.5)	3, 4, 5, 9	132.24/132.49, CH	6.49–6.52/6.56–6.58, d (12.5)	3, 4, 5, 9
8	118.90/118.72, CH	7.11–7.15/6.99–7.02, d (15.5)	4, 7, 9	123.90/124.59, CH	6.13–6.16/6.09–6.12, d (12.5)	4, 7, 9
9	168.96/168.80, C	-	-	171.13/171.01, C	-	-
10	48.20/47.14, CH_2_	3.89/3.74, t (7.2)	9, 11, 12, 21	48.97/45.75, CH_2_	3.65/3.73, t (7.5)	9, 11, 12, 21
11	30.86/29.16, CH_2_	2.22/2.16, m	10, 12	29.32/28.68, CH_2_	2.02/2.18, m	10,12
12	71.94/72.64, CH_2_	4.11/4.05, t (6.4)	10, 11, 13	72.03/72.32, CH_2_	3.78/4.04, t (6.5)	10, 11, 13
13	153.88/153.94, C	-	-	153.55/153.86, C	-	-
14	119.57, C	-	-	119.36/119.59, C	-	-
15	134.63/134.56, CH	7.57, br s	13, 14, 17, 18, 19	134.51/134.57, CH	7.51/7.56, s	13, 14, 17, 18, 19
16	136.81/136.99, C	-	-	136.85, C	-	-
17	134.63/134.56, CH	7.57, br s	13, 14, 15, 18, 19	134.51/134.57, CH	7.51/7.56, s	13, 14, 15, 18, 19
18	119.57, C	-	-	119.36/119.59, C	-	-
19	30.45, CH_2_	3.00, m	15, 16, 17, 20, 22, 23	30.51/30.56, CH_2_	3.00, m	15, 16, 17, 20, 22, 23
20	59.24, CH_2_	3.30, m	16, 19, 22, 23	59.32/59.24, CH_2_	3.34, m	16, 19, 22, 23
21	34.74/36.55, CH_3_	3.09/3.28, s	9, 10	32.59/36.67, CH_3_	3.06/3.03, s	9, 10
22	43.62, CH_3_	2.91, s	20, 23	43.70/43.73, CH_3_	2.91, s	20, 23
23	43.62, CH_3_	2.91, s	20, 22	43.70/43.73, CH_3_	2.91, s	20, 22

^a^ Chemical shifts are given for both rotamers when distinguishable signals were identified.

**Table 2:** ^1^H NMR (500 MHz) and ^13^C NMR (125 MHz) data for Psammaplysenes H (**4**) and I (**5**) in CD_3_OD.

Position	Psammaplysene H (4)			Psammaplysene I (5)		
No	*δ*_C_, Type ^a^	*δ*_H_ mult, (*J* in Hz) ^a^	HMBC	*δ*_C_, Type ^a^	*δ*_H_ mult, (*J* in Hz) ^a^	HMBC
1	154.7, C	-	-	154.7, C	-	-
2	119.6, C	-	-	119.6, C	-	-
3	133.5/133.4, CH	7.92/7.85, d	1, 2, 5, 6, 7	133.5/133.4, CH	7.92/7.85, d	1, 2, 5, 6, 7
4	136.2, C	-	-	136.2, C	-	-
5	133.5/133.4, CH	7.92/7.85, d	1, 2, 3, 6, 7	133.5/133.4, CH	7.92/7.85, d	1, 2, 3, 6, 7
6	119.6, C	-	-	119.6, C	-	-
7	140.2/140.0, CH	7.40–7.44, m	3, 4, 5, 9	140.2/140.0, CH	7.40–7.44, m	3, 4, 5, 9
8	121.6/121.7, CH	7.14–7.27, dd (15.0)	4, 7, 9	121.6/121.7, CH	7.14–7.27, dd (15.0)	4, 7, 9
9	168.5, C ^a^	-	-	168.5, C ^a^	-	-
10	48.4/47.3, CH_2_	3.91/3.76, t (7.0)	9, 11, 12, 21	48.4/47.3, CH_2_	3.91/3.76, t (7.0)	9, 11, 12, 21
11	30.6/29.1, CH_2_	2.23/2.17, m	10, 12	30.6/29.1, CH_2_	2.23/2.17, m	10, 12
12	72.6/71.9, CH_2_	4.12/4.07, m	10, 11, 13	72.6/71.9, CH_2_	4.12/4.07, m	10, 11, 13
13	154.7, C	-	-	154.7, C	-	-
14	119.6, C	-	-	119.6, C	-	-
15	134.5, CH	7.56/7.53, m	13, 14, 17, 18, 19	134.5, CH	7.56/7.53, m	13, 14, 17, 18, 19
16	137.8, C ^b^	-	-	137.8, C ^b^	-	-
17	134.5, CH	7.56/7.53, m	13, 14, 15, 18, 19	134.5, CH	7.56/7.53, m	13, 14, 15, 18, 19
18	119.6, C	-	-	119.6, C	-	-
19	32.2, CH_2_	2.93, t (8.0)	16, 20, 22	32.2, CH_2_	2.93, t (8.0)	16, 20, 22
20	51.1, CH_2_	3.21, m	16, 19, 22	51.1, CH_2_	3.21, m	16, 19, 22
21	34.7/36.6, CH_3_	3.10/3.29, s	9, 10	34.7/36.6, CH_3_	3.10/3.29, s	9, 10
22	34.0, CH_3_	2.70, s	20	34.0, CH_3_	2.70, s	20
1′	57.2, CH_2_	3.27, m	1, 2′, 3′	48.7, CH_2_	3.35, m	1, 2′, 3′
2′	27.6, CH_2_	2.23, m	1′, 3′	30.9 CH_2_	2.23, m	1′, 3′
3′	71.9, CH_2_	4.18, m	1′, 2′, 4′, 5′	71.9, CH_2_	4.18, m	1′, 2′, 4′
4′	44.3, CH_3_	2.78/2.74, s	3′, 5′	34.0, CH_3_	2.77/2.76, s	3′
5′	44.3, CH_3_	2.79/2.74, s	3′, 4′	-	-	-

^a^ Chemical shifts are given for both rotamers when distinguishable signals were identified; ^b^ Detected by HMBC correlations.

**Table 3:** ^1^H NMR (500 MHz) and ^13^C NMR (125 MHz) data for Anomoians C (**6**) and D (**7**) in CD_3_OD.

Position	Anomoian C (6)			Anomoian D (7)		
No	*δ*_C_, Type ^a^	*δ*_H_ mult, (*J* in Hz) ^a^	HMBC	*δ*_C_, Type ^a^	*δ*_H_ mult, (*J* in Hz) ^a^	HMBC
1	151.65/151.47, C	-	-	152.7, C	-	-
2	112.36, C	-	-	112.69, C	-	-
3	134.47/134.41, CH	7.34/7.32, s	1, 2, 4, 5, 6, 7	134.40, CH	7.37/7.35, s	1, 2, 4, 5, 6, 7
4	132.77/133.49, C	-	-	130.53/129.90, C	-	-
5	134.47/134.41, CH	7.34/7.32, s	1, 2, 3, 4, 6, 7	134.40, CH	7.37/7.35, s	1, 2, 3, 4, 6, 7
6	112.36, C	-	-	112.69, C	-	-
7	33.14/32.14, CH_2_	2.90/2.83–2.99, m	3, 4, 5, 8, 9	37.40/38.02, CH_2_	2.83–3.04/2.92, m	3, 4, 5, 8, 9
8	66.86, CH	3.82, m	7, 9, 24, 25	60.79/61.29, CH	4.26/4.17, m	7, 9, 24
9	172.36/172.63, C	-	-	171.40/172.00, C	-	-
10	47.14/48.24, CH_2_	3.40–3.72, m	9, 11, 21	47.67/47.78, CH_2_	3.48–3.80/3.26–3.51, m	9, 11, 21
11	29.09/30.45, CH_2_	1.92–2.00/1.73–1.92, m	10, 12	29.15/30.50, CH_2_	1.95–2.05/1.95, m	10, 12
12	72.31/71.72, CH_2_	3.85–3.93/3.88, m	11, 13	72.35/71.61, CH_2_	3.97/3.99, m	11, 13
13	153.61/153.30, C	-	-	153.72, C	-	-
14	119.43, C	-	-	119.47, C	-	-
15	134.47/134.50, CH	7.52/7.53, s	13, 14, 16, 17, 18, 19	134.51/134.53, CH	7.54/7.55, s	13, 14, 16, 17, 18, 19
16	137.97/138.24, C	-	-	137.72, C	-	-
17	134.47/134.50, CH	7.52/7.53, s	13, 14, 15, 16, 18, 19	134.51/134.53, CH	7.54/7.55, s	13, 14, 15, 16, 18, 19
18	119.43, C	-	-	119.47, C	-	-
19	31.40/31.46, CH_2_	2.92, m	15, 16, 17, 20	31.22, CH_2_	2.93, m	15, 16, 17, 20
20	60.12/60.14, CH_2_	3.08, m	16, 19, 22, 23	59.96, CH_2_	3.21, m	16, 19, 22, 23
21	36.64/34.26, CH_3_	2.92/2.93, s	9, 10	36.25/34.29, CH_3_	2.50/2.47, s	9, 10
22	44.30/44.34, CH_3_	2.72/2.71, s	20, 23	44.18/44.21, CH_3_	2.74/2.74, s	20, 23
23	44.30/44.34, CH_3_	2.72/2.71, s	20, 22	44.18/44.21, CH_3_	2.74/2.74, s	20, 22
24	42.31/41.19, CH_3_	2.45/2.42, s	8, 25	33.26/33.68, CH_3_	2.82/3.00, s	8
25	42.31/41.19, CH_3_	2.45/2.42, s	8, 24	-	-	-

^a^Chemical shifts are given for both rotamers when distinguishable signals were identified.

**Table 4:** ^1^H NMR (500 MHz) and ^13^C NMR (125 MHz) data for Anomoians E (**8**) and F (**9**) in CD_3_OD.

Position	Anomoian E (8)			Anomoian F (9)		
No	*δ*_C_, Type ^a^	*δ*_H_ mult, (*J* in Hz) ^a^	HMBC	*δ*_C_, Type ^a^	*δ*_H_ mult, (*J* in Hz) ^a^	HMBC
1	153.29/153.15, C	-	-	152.76/152.57, C	-	-
2	119.08.119.14, C	-	-	118.92, C	-	-
3	135.35/135.19, CH	7.51/7.48, s	1, 2, 5, 6, 7	135.32/135.22, CH	7.51/7.48, s	1, 2, 5, 6, 7
4	137.14/138.15, C	-	-	138.66/137.72, C	-	-
5	135.35/135.19, CH	7.51/7.48, s	1, 2, 3, 6, 7	135.32/135.22, CH	7.51/7.48, s	1, 2, 3, 6, 7
6	119.08.119.14, C	-	-	118.92, C	-	-
7	38.03/38.65, CH_2_	2.84–3.03/2.93, m	3, 4, 5, 8, 9	33.05/31.91, CH_2_	3.00/2.92–3.07, m	3, 4, 5, 8, 9
8	60.72/61.23, CH	4.15/4.06, m	4, 7, 9, 24	66.58/66.79, CH	3.97/3.89, m	4, 7, 9, 24, 25
9	172.73/173.46, C	-	-	172.10/171.65, C	-	-
10	47.57/47.82, CH_2_	3.51–3.76/3.26–3.60, m	9, 11, 12, 21	48.26/47.36, CH_2_	3.41–3.74/3.51–3.64, m	9, 11, 12, 21
11	29.16/30.64, CH_2_	1.95–2.03/1.95, m	10, 12	30.71/29.08, CH_2_	1.69–1.98/1.90–1.98, m	10, 12
12	72.36/71.71, CH_2_	3.99, m	10, 11, 13	71.82/72.40, CH_2_	3.94/3.95, m	10, 11, 13
13	153.54, C	-	-	153.77/153.51, C	-	-
14	119.42, C	-	-	119.52, C	-	-
15	134.54/134.52, CH	7.55/7.56, s	13, 14, 17, 18, 19	134.57/134.60, CH	7.57/7.58, s	13, 14, 17, 18, 19
16	137.74/138.52, C	-	-	137.28, C	-	-
17	134.54/134.52, CH	7.55/7.56, s	13, 14, 15, 18, 19	134.57/134.60, CH	7.57/7.58, s	13, 14, 15, 18, 19
18	119.42, C	-	-	119.52, C	-	-
19	31.31/31.41, CH_2_	2.93, m	15, 16, 17, 20	30.67/30.59, CH_2_	2.99, m	15, 16, 17, 20
20	60.01/60.08, CH_2_	3.09, m	16, 19, 22, 23	59.43, CH_2_	3.26, m	16, 19, 22, 23
21	36.24/34.33, CH_3_	2.88/2.99, s	9, 10	34.31/36.64, CH_3_	2.92/2.94, s	9, 10
22	44.19/44.24, CH_3_	2.73/2.71, s	20, 23	43.75, CH_3_	2.85/2.85, s	20, 23
23	44.19/44.24, CH_3_	2.73/2.71, s	20, 22	43.75, CH_3_	2.85/2.85, s	20, 22
24	33.71/34.11, CH_3_	2.42/2.39, s	4, 8	42.23/42.07, CH_3_	2.51/2.45, s	4, 8, 25
25	-	-	-	42.23/42.07, CH_3_	2.51/2.45, s	4, 8, 24
1′	71.66/71.62, CH_2_	4.09, m	1, 2′, 3′	71.44, CH_2_	4.01, t (5.5)	1, 2′, 3′
2′	26.95, CH_2_	2.25, m	1′, 3′	26.62, CH_2_	2.27, m	1′, 3′
3′	57.15, CH_2_	3.36, m	1′, 2′, 4′, 5′	57.07, CH_2_	3.45, m	1′, 2′, 4′, 5′
4′	44.05, CH_3_	2.85, s	3′, 5′	43.79, CH_3_	2.92/2.91, s	3′, 5′
5′	44.05, CH_3_	2.85, s	3′, 4′	43.79, CH_3_	2.92/2.91, s	3′, 4′

^a^ Chemical shifts are given for both rotamers when distinguishable signals were identified.

**Table 5:** Cytotoxic activities in vitro (KB Cell Line) for compounds **1**–**3**, **6**–**11**.

Compounds	10 μM ^a^	1 μM ^a^
Psammaplysene D (**1**)	100 ± 0.2	95 ± 0.5
Psammaplysene F (**2**)	73 ± 2	20 ± 0.2
Psammaplysene G (**3**)	75 ± 0.4	17 ± 1
Anomoian C (**6**)	28 ± 5	15 ± 3
Anomoian D (**7**)	29 ± 2	17 ± 2
Anomoian E (**8**)	82 ± 1	6 ± 2
Anomoian F (**9**)	100 ± 0.5	20 ± 0.6
*N*,*N*-dimethyldibromotyramine (**10**)	100 ± 1	89 ± 1
5-hydroxy xanthenuric acid (**11**)	25 ± 1	6 ± 0.5

^a^ Cell proliferation was measured with Celltiter 96 Aqueous One solution reagent (Promega), and results are expressed as the percentage of inhibition of cellular proliferation of KB cells treated for 72 h with compounds compared to cells treated with DMSO only (mean ± SE of triplicate).

**Table 6:** AChE inhibition of bromotyrosine compounds isolated from Verongiida sponges.

Sponge/Compounds	AChE Origin Activities		Ref.
*Pseudoceratina* purpurea Purealidine Q Isoanomoian A Aplysanzine A	Eel IC_50_ = 1.2 µM (NCI ^a^) IC_50_ = 70 µM (NCI) IC_50_ = 104 µM (NCI)		Olatunji et al. 2014 [31]
*Acanthodendrilla* sp. Homoaerothionin Fistularin 1	Human recombinant AchE IC_50_ = 4.5 µM (CI ^b^) IC_50_ = 47.5 µM (ND ^c^)		Sirimangkalakitti et al. 2015 [32]
Unidentified *Verongida* Aplysamine-4	Eel K_I_ = 16 µM (NCI)	Insect recombinant K_I_ = 2 µM (NCI)	Sepcic et al. 2001 [33]

^a^ NCI: non competitive inhibition; ^b^ CI: competitive inhibition; ^c^ ND: non described.

## Supplementary Materials

The following are available online at http://www.mdpi.com/1660-3397/16/5/146/s1, Spectroscopic Data (HRMS, ^1^H NMR, ^13^C NMR, COSY, HSQC and HMBC) for compounds **1**–**12**.
