# Supplementary material for: Bioactive Bromotyrosine-Derived Alkaloids from the Polynesian Sponge Suberea ianthelliformis
Source: Mar Drugs. 2018 Apr 27;16(5):146. doi: 10.3390/md16050146 (PMC5983277; doi:10.3390/md16050146)
Supplement: Supplementary file 1 [file marinedrugs-16-00146-s001.pdf]

Supporting Information

Bioactive Bromotyrosine-Derived Alkaloids from the Polynesian Sponge *Suberea ianthelliformis*

Amr El-Demerdash,[1,2] Céline Moriou,[1] Jordan Toullec,[3] Marc Besson,[4,5] Stéphanie Soulet,[6] Nelly Schmitt,[6] Sylvain Petek,[3] David Lecchini,[4] Cécile Debitus*[3] Ali Al-Mourabit [1*]

1. Institut de Chimie des Substances Naturelles, CNRS UPR 2301, Univ. Paris-Sud, University of Paris-Saclay, 1, Avenue de la Terrasse, 91198 Gif-Sur-Yvette, France email: eldemerdash555@gmail.com, Celine.Moriou@cnrs.fr, Ali.ALMOURABIT@cnrs.fr
2. Organic Chemistry Division, Chemistry Department, Faculty of Science, Mansoura University, Mansoura-35516, Egypt
3. LEMAR, IRD, UBO, CNRS, IFREMER, IUEM, Plouzané, France, email : Jordan.Toullec@univ-brest.fr, sylvain.petek@ird.fr, cecile.debitus@ird.fr
4. CRIOBE, CNRS, EPHE, UPVD, PSL Research University, Moorea, French Polynesia, email: bessonmarcluc@gmail.com, david.lecchini@ephe.sorbonne.fr
5. Observatoire Océanologique de Banyuls-sur-Mer, Université Pierre et Marie Curie Paris, Banyuls-sur-Mer, France
6. EIO, UPF, ILM, IFREMER, IRD, Faa'a, Tahiti, French Polynesia, email: stephanie.soulet@upf.pf, nelly.schmitt@upf.pf

❖ Correspondance: Ali Al-Mourabit ; Ali.ALMOURABIT@cnrs.fr

Table of Contents

Figure S1. ^{1}H NMR spectrum of Psammaplysene D (**1**) in MeOD (500 MHz) 4
Figure S2. ^{13}C NMR spectrum of Psammaplysene D (**1**) in MeOD (500 MHz) 5
Figure S3. HR-ESI mass spectrum of Psammaplysene D (**1**) 6
Figure S10. ^{1}H NMR spectrum of Psammaplysene F (**2**) in MeOD (500 MHz) 7
Figure S11. ^{13}C NMR spectrum of Psammaplysene F (**2**) in MeOD (500 MHz) 8
Figure S12. HSQC NMR spectrum of Psammaplysene F (**2**) in MeOD (500 MHz) 9
Figure S13. ^{1}H–^{1}H COSY NMR spectrum of Psammaplysene F (**2**) in MeOD (500 MHz) 10
Figure S14. ^{1}H-^{13}C HMBC NMR spectrum of Psammaplysene F (**2**) in MeOD (500 MHz) 11
Figure S15. HR-ESI mass spectrum of Psammaplysene F (**2**) 12
Figure S16. ^{1}H NMR spectrum of Psammaplysene G (**3**) in MeOD (500 MHz) 13
Figure S17. ^{13}C NMR spectrum of Psammaplysene G (**3**) in MeOD (500 MHz) 14
Figure S18. HSQC NMR spectrum of Psammaplysene G (**3**) in MeOD (500 MHz) 15
Figure S19. ^{1}H–^{1}H COSY NMR spectrum of Psammaplysene G (**3**) in MeOD (500 MHz) 16
Figure S20. ^{1}H-^{13}C HMBC NMR spectrum of Psammaplysene G (**3**) in MeOD (500 MHz) 17
Figure S21. HR-ESI mass spectrum of Psammaplysene G (**3**) 18
Figure S4. ^{1}H NMR spectrum of Psammaplysene H (**4**) et I (**5**) in MeOD (500 MHz) 19
Figure S5. ^{13}C NMR spectrum of Psammaplysene H (**4**) et I (**5**) in MeOD (500 MHz) 20
Figure S6. HSQC NMR spectrum of Psammaplysene H (**4**) et I (**5**) in MeOD (500 MHz) 21
Figure S7. ^{1}H–^{1}H COSY NMR spectrum of Psammaplysene H (**4**) et I (**5**) in MeOD (500 MHz) 22
Figure S8. ^{1}H-^{13}C HMBC NMR spectrum of Psammaplysene H (**4**) et I (**5**) in MeOD (500 MHz) 23
Figure S9. HR-ESI mass spectrum of Psammaplysene H (**4**) et I (**5**) 24
Figure S22. ^{1}H NMR spectrum of Anomoian C (**6**) in MeOD (500 MHz) 25
Figure S23. ^{13}C NMR spectrum of Anomoian C (**6**) in MeOD (500 MHz) 26
Figure S24. HSQC NMR spectrum of Anomoian C (**6**) in MeOD (500 MHz) 27
Figure S25. ^{1}H–^{1}H COSY NMR spectrum of Anomoian C (**6**) in MeOD (500 MHz) 28
Figure S26. ^{1}H-^{13}C HMBC NMR spectrum of Anomoian C (**6**) in MeOD (500 MHz) 29
Figure S27. HR-ESI mass spectrum of Anomoian C (**6**) 30
Figure S28. ^{1}H NMR spectrum of Anomoian D (**7**) in MeOD (500 MHz) 31
Figure S29. ^{13}C NMR spectrum of Anomoian D (**7**) in MeOD (500 MHz) 32
Figure S30. HSQC NMR spectrum of Anomoian D (**7**) in MeOD (500 MHz) 33
Figure S31. ^{1}H–^{1}H COSY NMR spectrum of Anomoian D (**7**) in MeOD (500 MHz) 34
Figure S32. ^{1}H-^{13}C HMBC NMR spectrum of Anomoian D (**7**) in MeOD (500 MHz) 35
Figure S33. HR-ESI mass spectrum of Anomoian D (**7**) 36
Figure S34. ^{1}H NMR spectrum of Anomoian E (**8**) in MeOD (500 MHz) 37
Figure S35. ^{13}C NMR spectrum of Anomoian E (**8**) in MeOD (500 MHz) 38
Figure S36. HSQC NMR spectrum of Anomoian E (**8**) in MeOD (500 MHz) 39
Figure S37. ^{1}H–^{1}H COSY NMR spectrum of Anomoian E (**8**) in MeOD (500 MHz) 40
Figure S38. ^{1}H-^{13}C HMBC NMR spectrum of Anomoian E (**8**) in MeOD (500 MHz) 41
Figure S39. HR-ESI mass spectrum of Anomoian E (**8**) 42
Figure S40. ^{1}H NMR spectrum of Anomoian F (**9**) in MeOD (500 MHz) 43
Figure S41. ^{13}C NMR spectrum of Anomoian F (**9**) in MeOD (500 MHz) 44
Figure S42. HSQC NMR spectrum of Anomoian F (**9**) in MeOD (500 MHz) 45
Figure S43. ^{1}H–^{1}H COSY NMR spectrum of Anomoian F (**9**) in MeOD (500 MHz) 46
Figure S44. ^{1}H-^{13}C HMBC NMR spectrum of Anomoian F (**9**) in MeOD (500 MHz) 47
Figure S45. HR-ESI mass spectrum of Anomoian F (**9**) 48
Figure S46. ^{1}H NMR spectrum of N, N-dimethyldibromotyramine (**10**) in MeOD (500 MHz) 49
Figure S47. ^{13}C NMR spectrum of N, N-dimethyldibromotyramine (**10**) in MeOD (500 MHz) 50
Figure S48. HR-ESI mass spectrum of N, N-dimethyldibromotyramine (**10**) 51
Figure S49. ^{1}H NMR spectrum of 4,5,8-trihydroxyquinoline-2-carboxylic acid (**11**) in MeOD (500 MHz) 52
Figure S50. ^{13}C NMR spectrum of 4,5,8-trihydroxyquinoline-2-carboxylic acid (**11**) in MeOD (500 MHz) 53

Figure S51. ^{1}H NMR spectrum of 4,5,8-trihydroxyquinoline-2-carboxylic acid (**11**) in DMF-d_7 (600 MHz)................54
Figure S52. ^{1}H NMR spectrum of 4,5,8-trihydroxyquinoline-2-carboxylic acid (**11**) in DMSO (500 MHz)..................55
Figure S53. HR-ESI mass spectrum of 4,5,8-trihydroxyquinoline-2-carboxylic acid (**11**)..56
Figure S54. ^{1}H NMR spectrum of Xantherunic acid (**12**) in MeOD (600 MHz)...57
Figure S55. ^{13}C NMR spectrum of Xantherunic acid (**12**) in MeOD (600 MHz)...58
Figure S56. HR-ESI mass spectrum of Xantherunic acid (**12**)..59

Figure S1. ^{1}H NMR spectrum of Psammaplysene D (**1**) in MeOD (500 MHz)

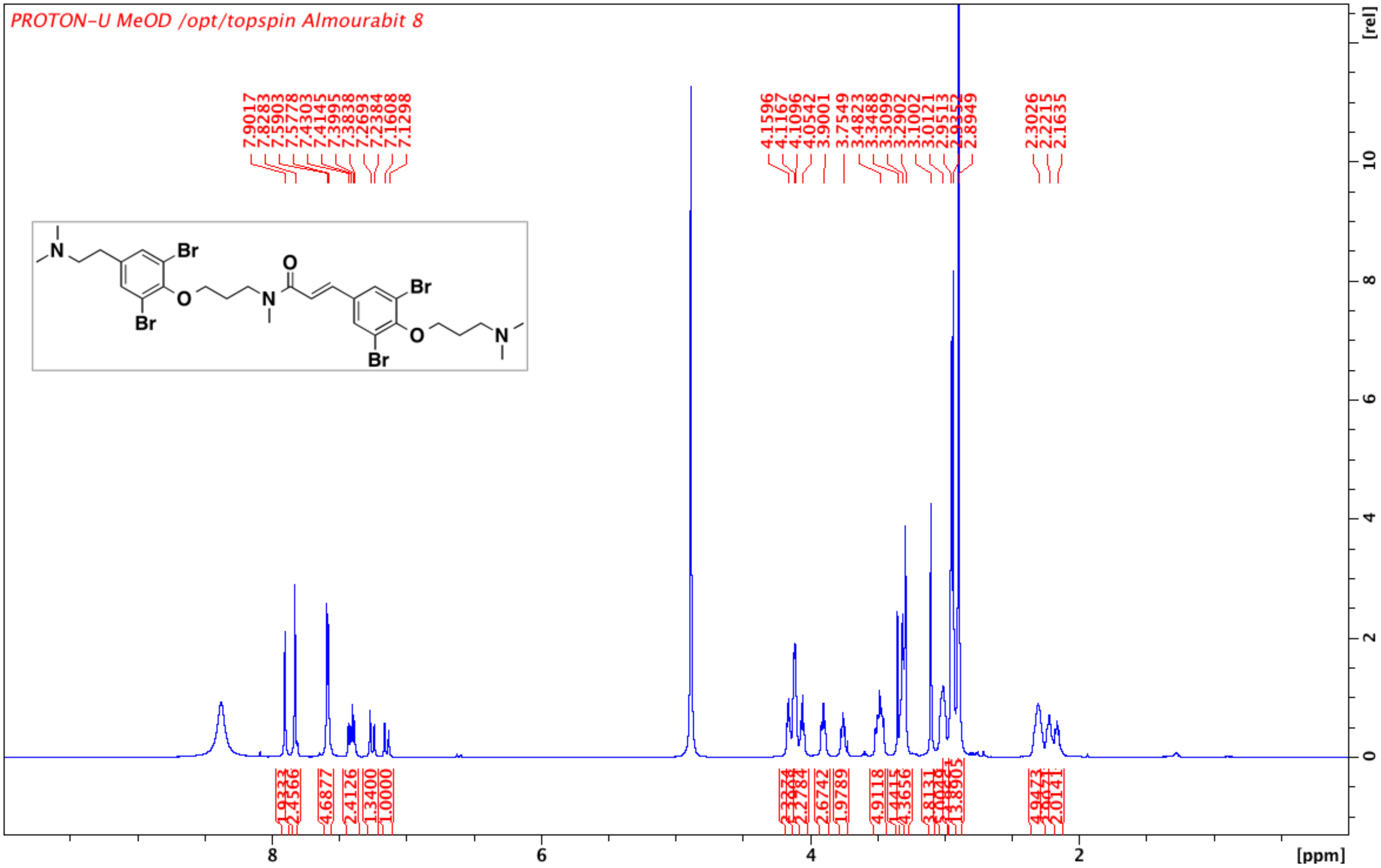

Figure S2. ^{13}C NMR spectrum of Psammaplysene D (**1**) in MeOD (500 MHz)

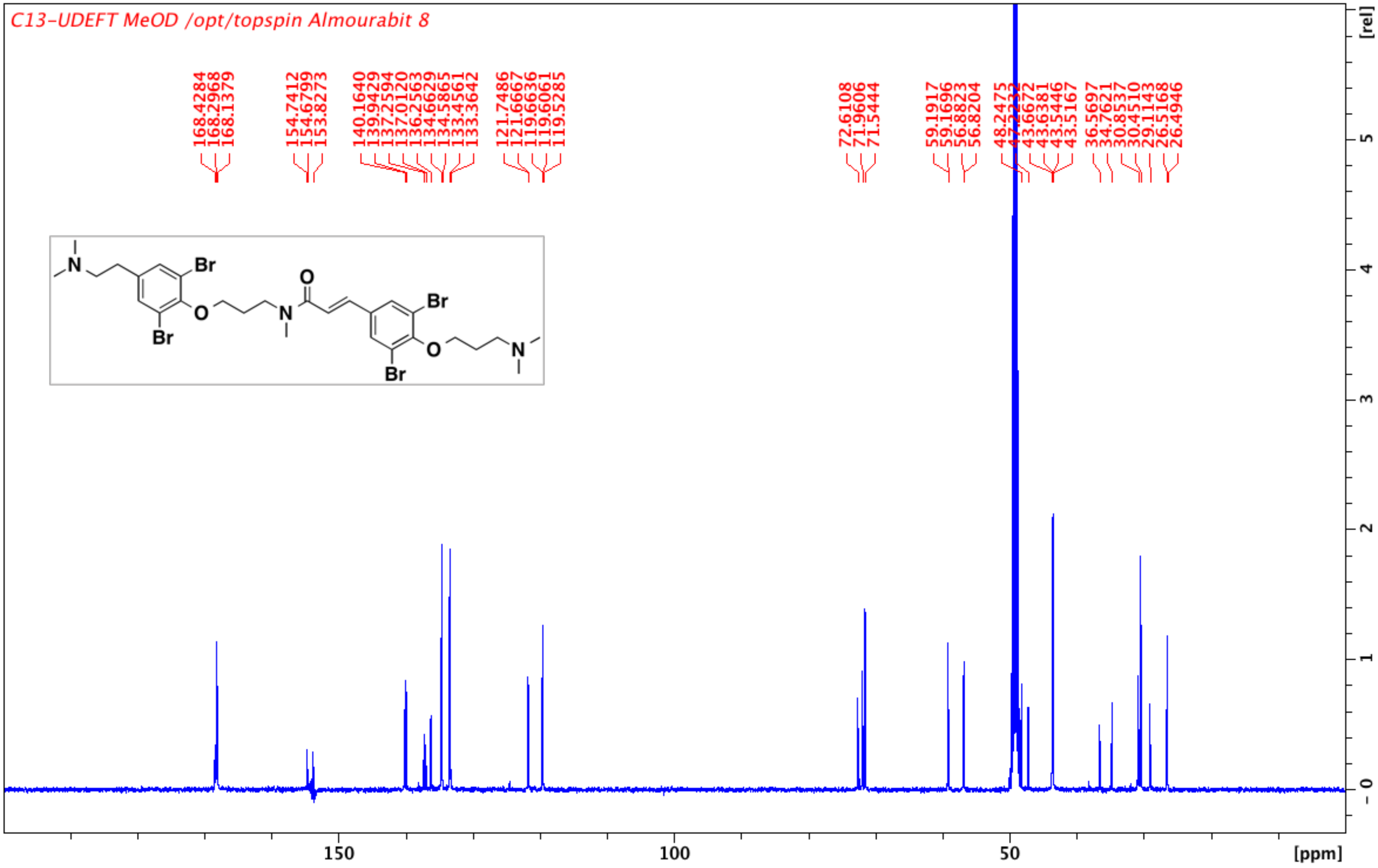

Figure S3. HR-ESI mass spectrum of Psammaplysene D (**1**)

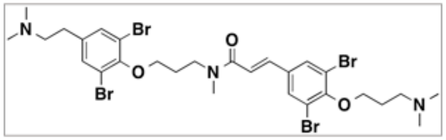

Elemental Composition Report

Single Mass Analysis

Tolerance = 9.0 PPM / DBE: min = -1.5, max = 100.0
Element prediction: Off
Number of isotope peaks used for i-FIT = 9

Monoisotopic Mass, Even Electron Ions
579 formula(e) evaluated with 7 results within limits (up to 50 closest results for each mass)
Elements Used:
C: 0-50 H: 0-100 N: 0-10 O: 0-10 79Br: 2-2 81Br: 2-2

ALMOURABIT_ahmed148-1 329 (1.506) Cm (327:329)

1: TOF MS ES+
2.55e+005

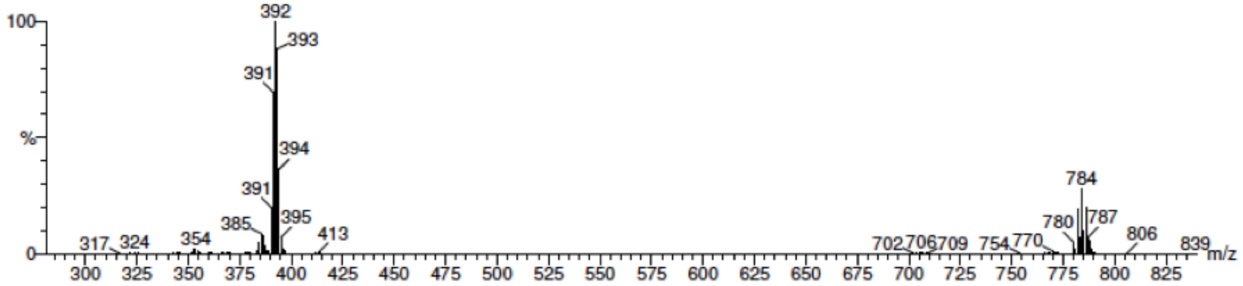

Minimum:				-1.5			
Maximum:		200.0	9.0	100.0			
Mass	Calc. Mass	mDa	PPM	DBE	i-FIT	i-FIT (Norm)	Formula
783.9673	783.9678	-0.5	-0.6	5.5	104.3	1.9	C22 H38 N7 O4 79Br2 81Br2
	783.9664	0.9	1.1	0.5	104.4	2.0	C21 H42 N3 O8 79Br2 81Br2
	783.9646	2.7	3.4	13.5	104.4	2.0	C33 H38 N O 79Br2 81Br2
	783.9705	-3.2	-4.1	4.5	104.3	1.9	C26 H42 N O6 79Br2 81Br2
	783.9638	3.5	4.5	1.5	104.4	2.0	C17 H38 N9 O6 79Br2 81Br2
	783.9718	-4.5	-5.7	9.5	104.4	2.0	C27 H38 N5 O2 79Br2 81Br2
	783.9606	6.7	8.5	9.5	104.3	1.9	C28 H38 N3 O3 79Br2 81Br2

Figure S10. ^{1}H NMR spectrum of Psammaplysene F (**2**) in MeOD (500 MHz)

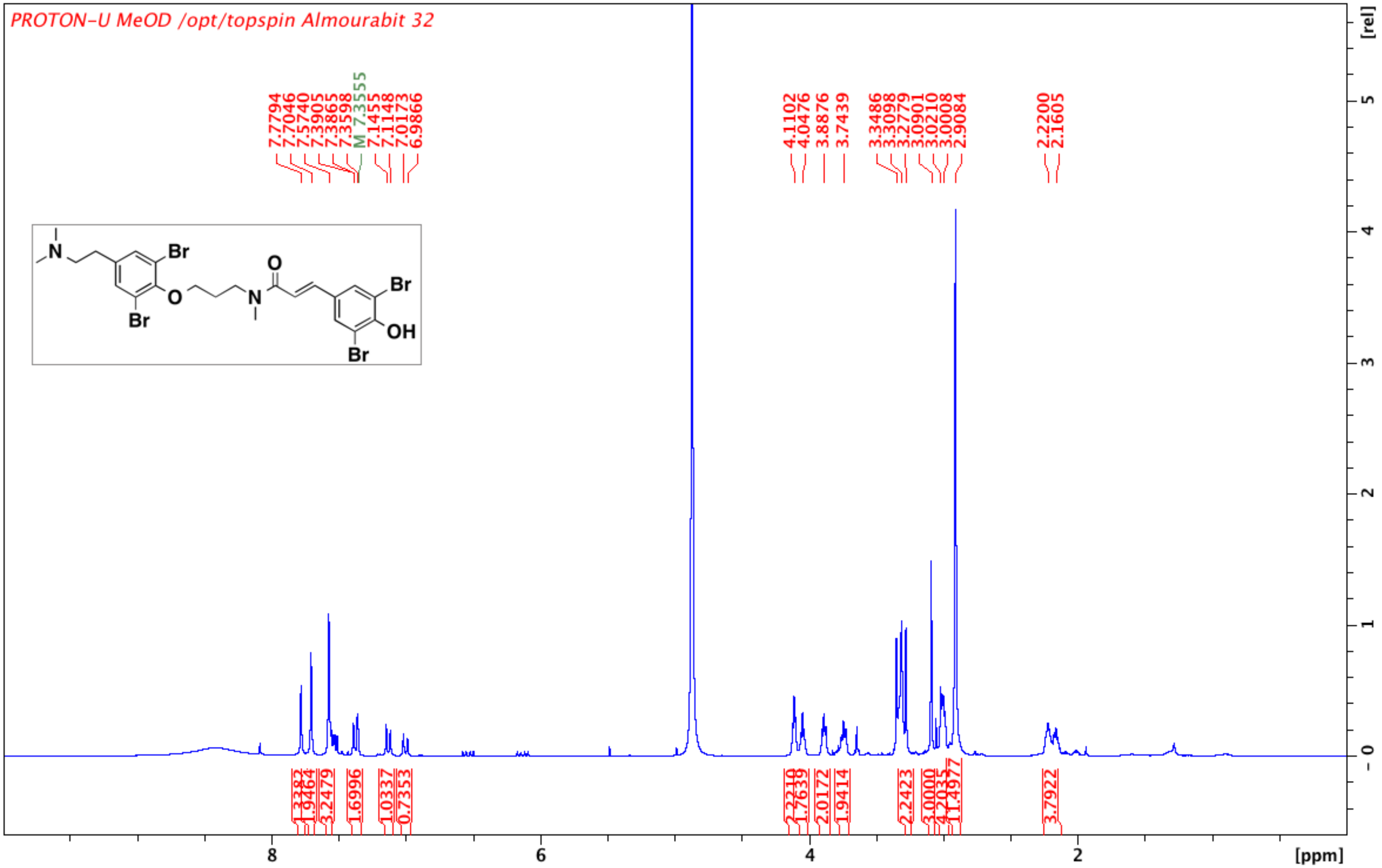

Figure S11. ^{13}C NMR spectrum of Psammaplysene F (**2**) in MeOD (500 MHz)

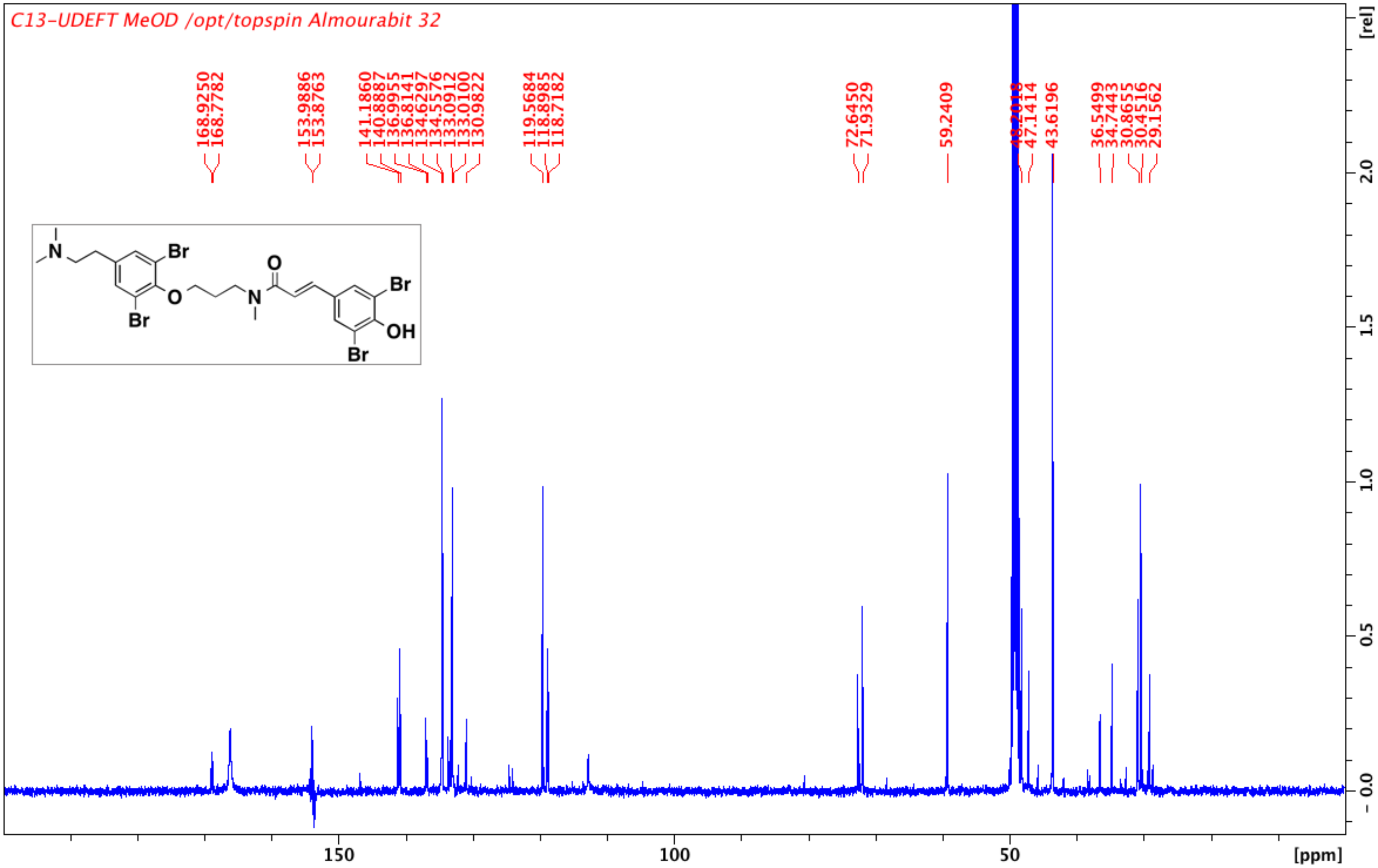

Figure S12. HSQC NMR spectrum of Psammaplysene F (**2**) in MeOD (500 MHz)

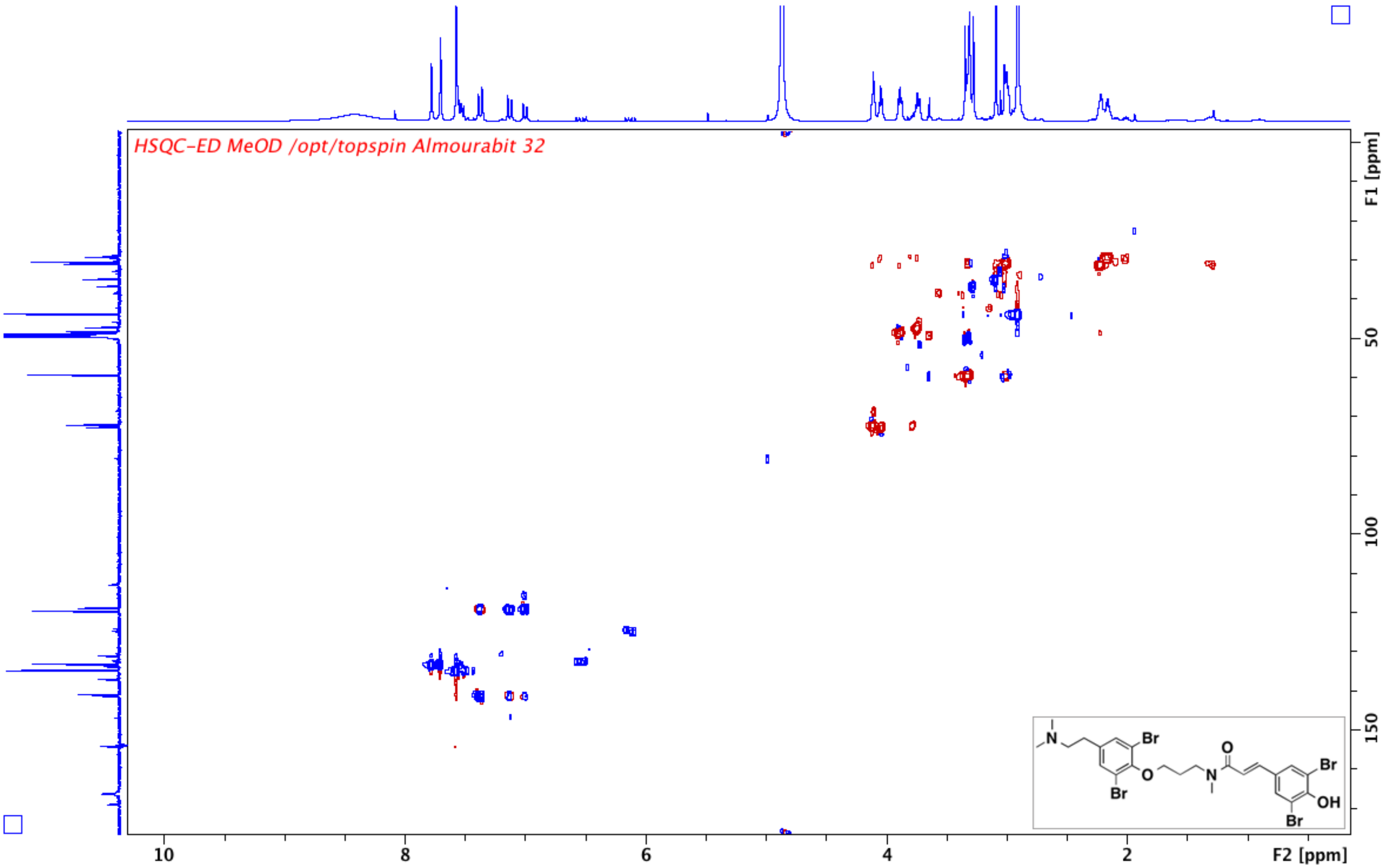

Figure S13. ^{1}H–^{1}H COSY NMR spectrum of Psammaplysene F (**2**) in MeOD (500 MHz)

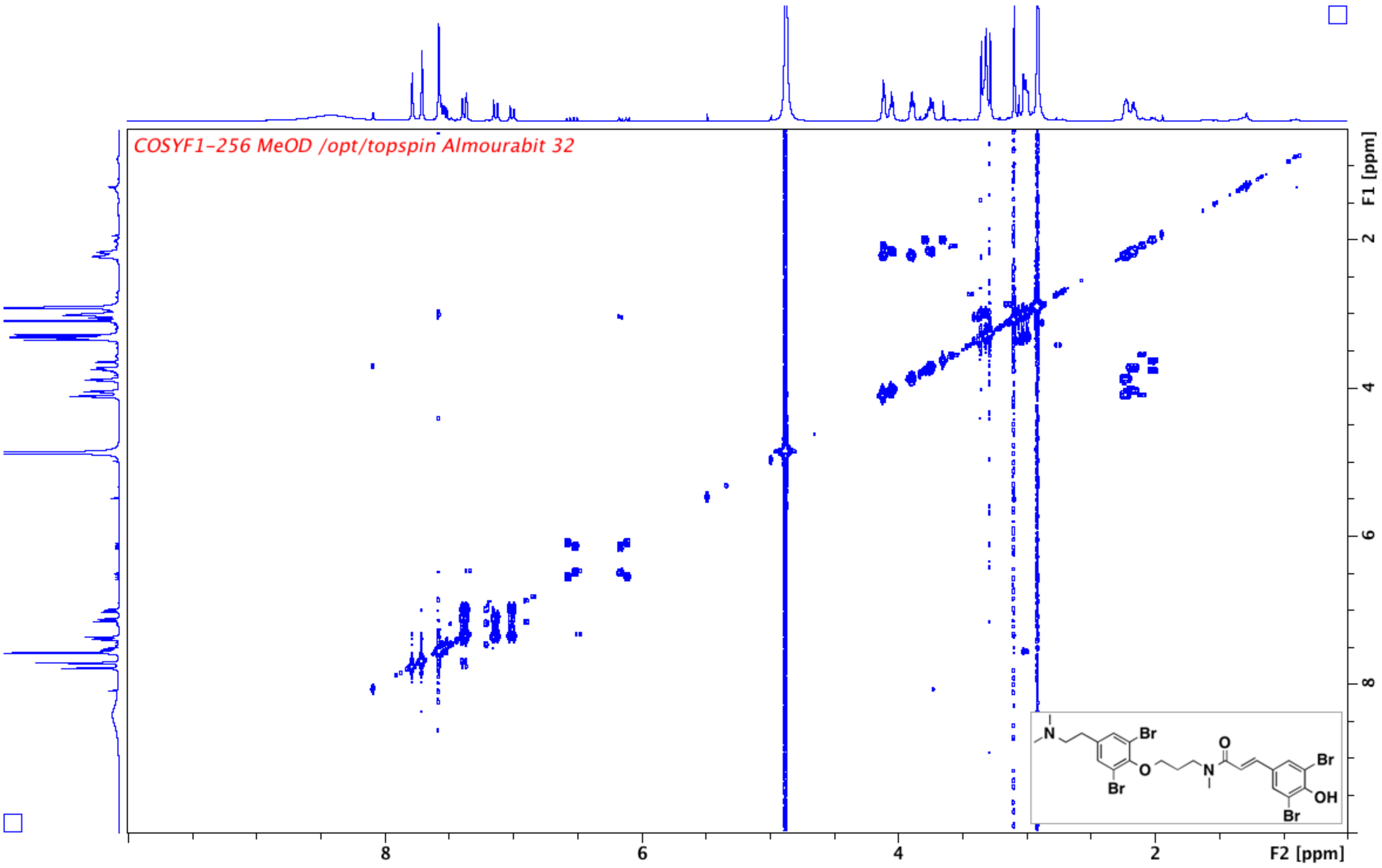

Figure S14. ^{1}H-^{13}C HMBC NMR spectrum of Psammaplysene F (**2**) in MeOD (500 MHz)

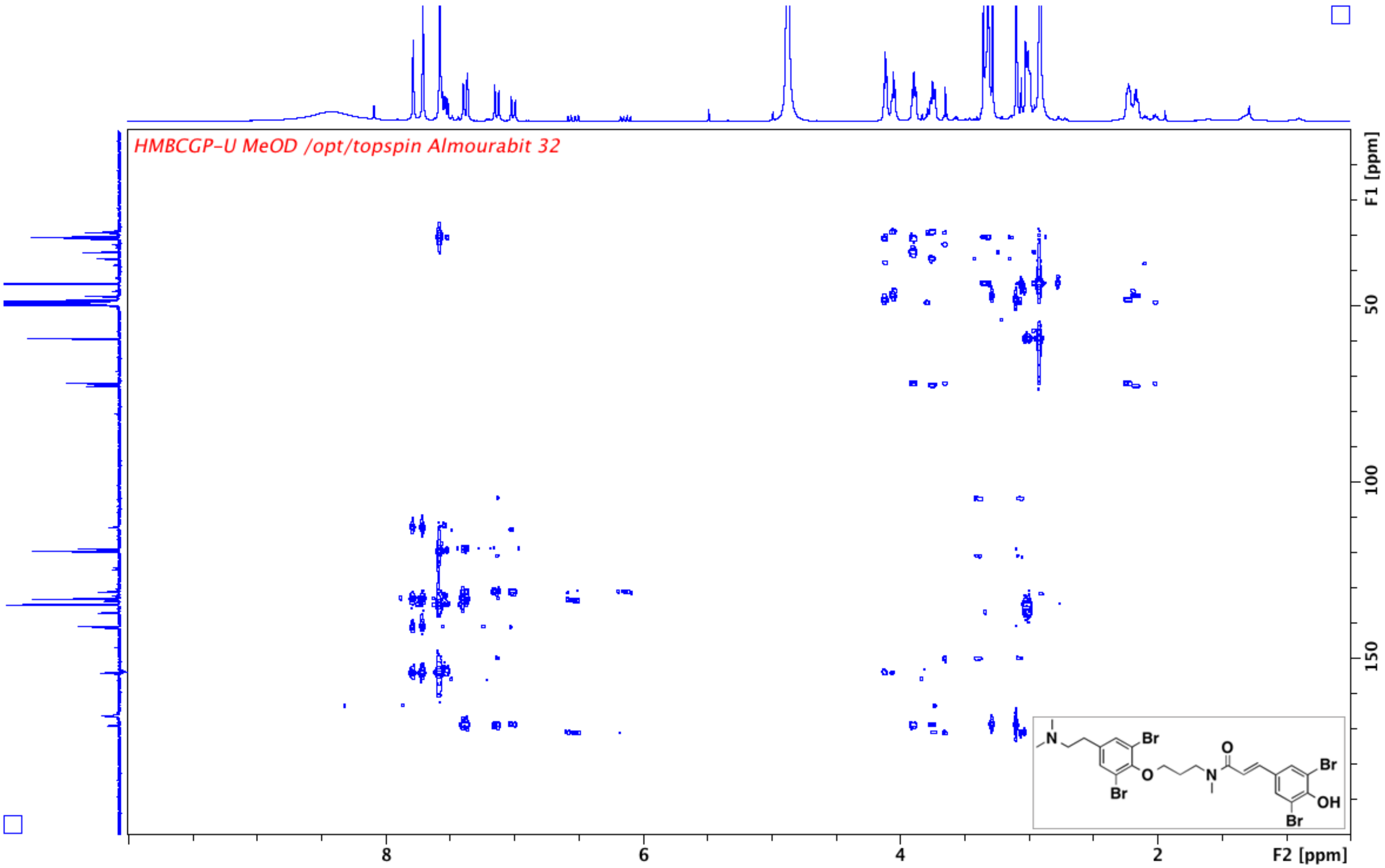

Figure S15. HR-ESI mass spectrum of Psammaplysene F (**2**)

Elemental Composition Report

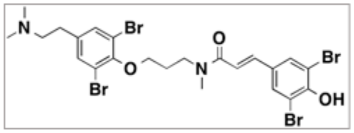

Single Mass Analysis

Tolerance = 10.0 PPM / DBE: min = -1.5, max = 100.0
Element prediction: Off
Number of isotope peaks used for i-FIT = 9

Monoisotopic Mass, Even Electron Ions
468 formula(e) evaluated with 7 results within limits (up to 50 best isotopic matches for each mass)
Elements Used:
C: 0-50 H: 0-100 N: 0-10 O: 0-10 79Br: 2-2 81Br: 2-2

ALMOURABIT_ahmed132-1 423 (1.899) Cm (421:428)

1: TOF MS ES+
4.69e+005

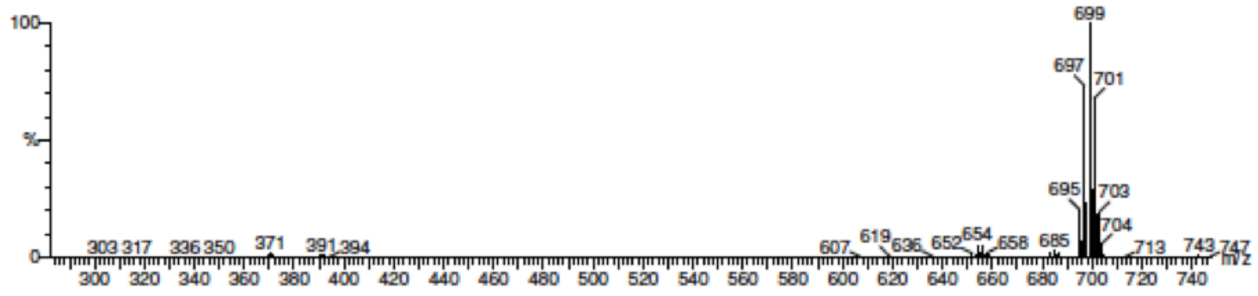

Minimum:				-1.5			
Maximum:		200.0	10.0	100.0			
Mass	Calc. Mass	mDa	PPM	DBE	i-FIT	i-FIT (Norm)	Formula
698.8675	698.8615	6.0	8.6	14.5	134.6	1.7	C25 H23 N4 79Br2 81Br2
	698.8714	-3.9	-5.6	9.5	134.7	1.8	C23 H27 N2 O3 79Br2 81Br2
	698.8674	0.1	0.1	5.5	134.9	2.0	C18 H27 N4 O5 79Br2 81Br2
	698.8661	1.4	2.0	0.5	134.9	2.0	C17 H31 O9 79Br2 81Br2
	698.8687	-1.2	-1.7	10.5	134.9	2.0	C19 H23 N8 O 79Br2 81Br2
	698.8634	4.1	5.9	1.5	135.1	2.2	C13 H27 N6 O7 79Br2 81Br2
	698.8647	2.8	4.0	6.5	135.1	2.2	C14 H23 N10 O3 79Br2 81Br2

Figure S16. ^{1}H NMR spectrum of Psammaplysene G (**3**) in MeOD (500 MHz)

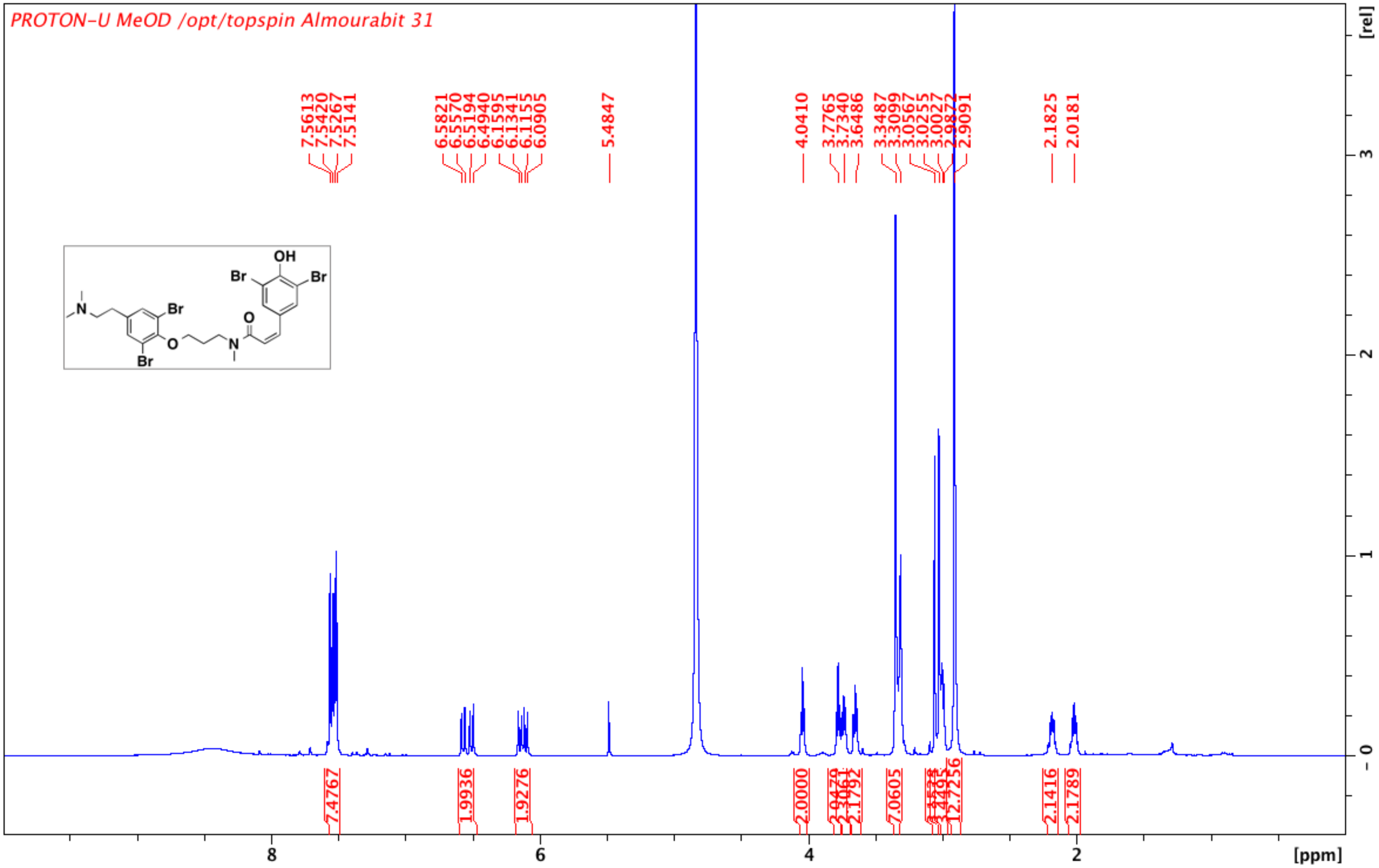

Figure S17. ^{13}C NMR spectrum of Psammaplysene G (**3**) in MeOD (500 MHz)

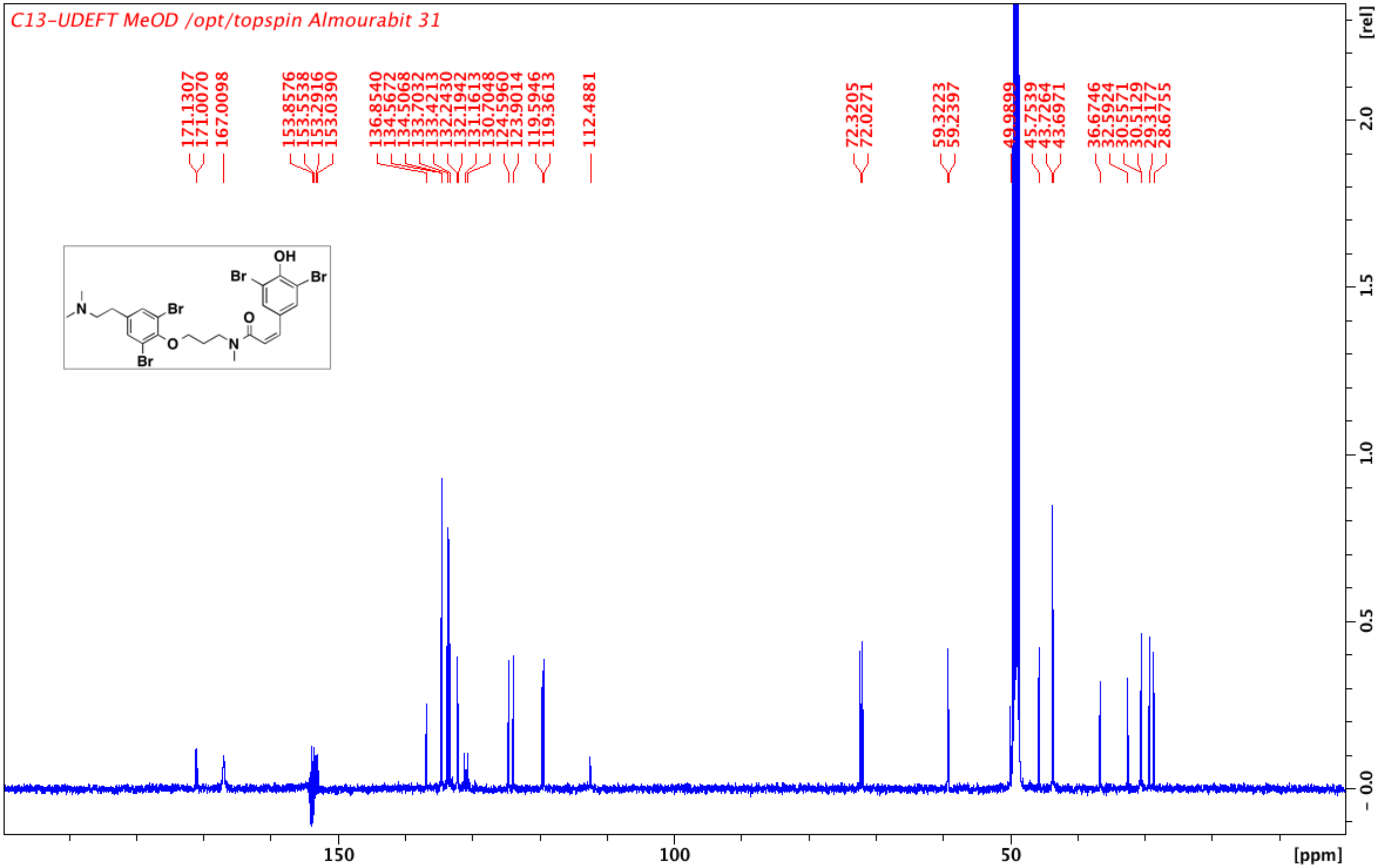

Figure S18. HSQC NMR spectrum of Psammaplysene G (**3**) in MeOD (500 MHz)

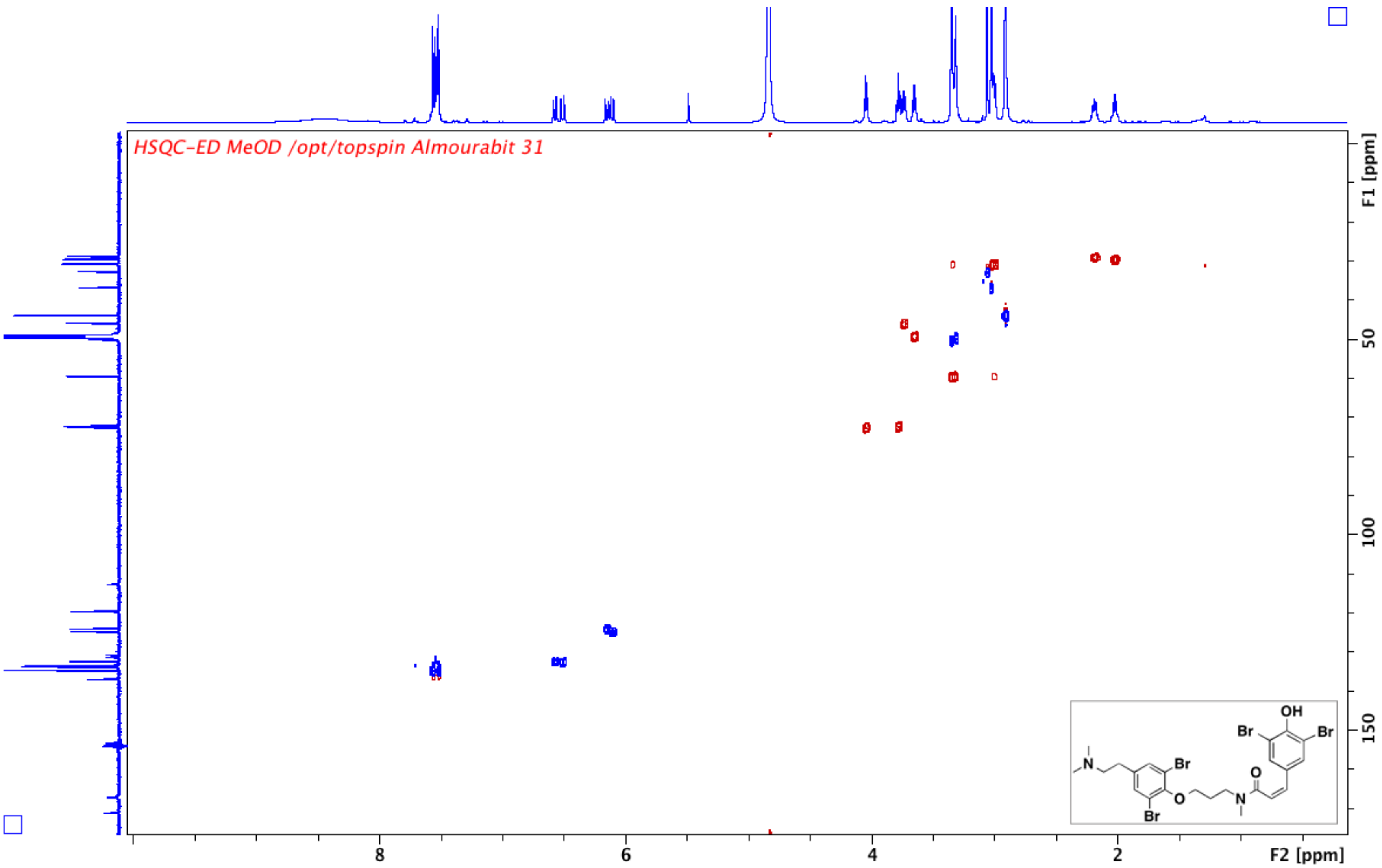

Figure S19. ^{1}H–^{1}H COSY NMR spectrum of Psammaplysene G (**3**) in MeOD (500 MHz)

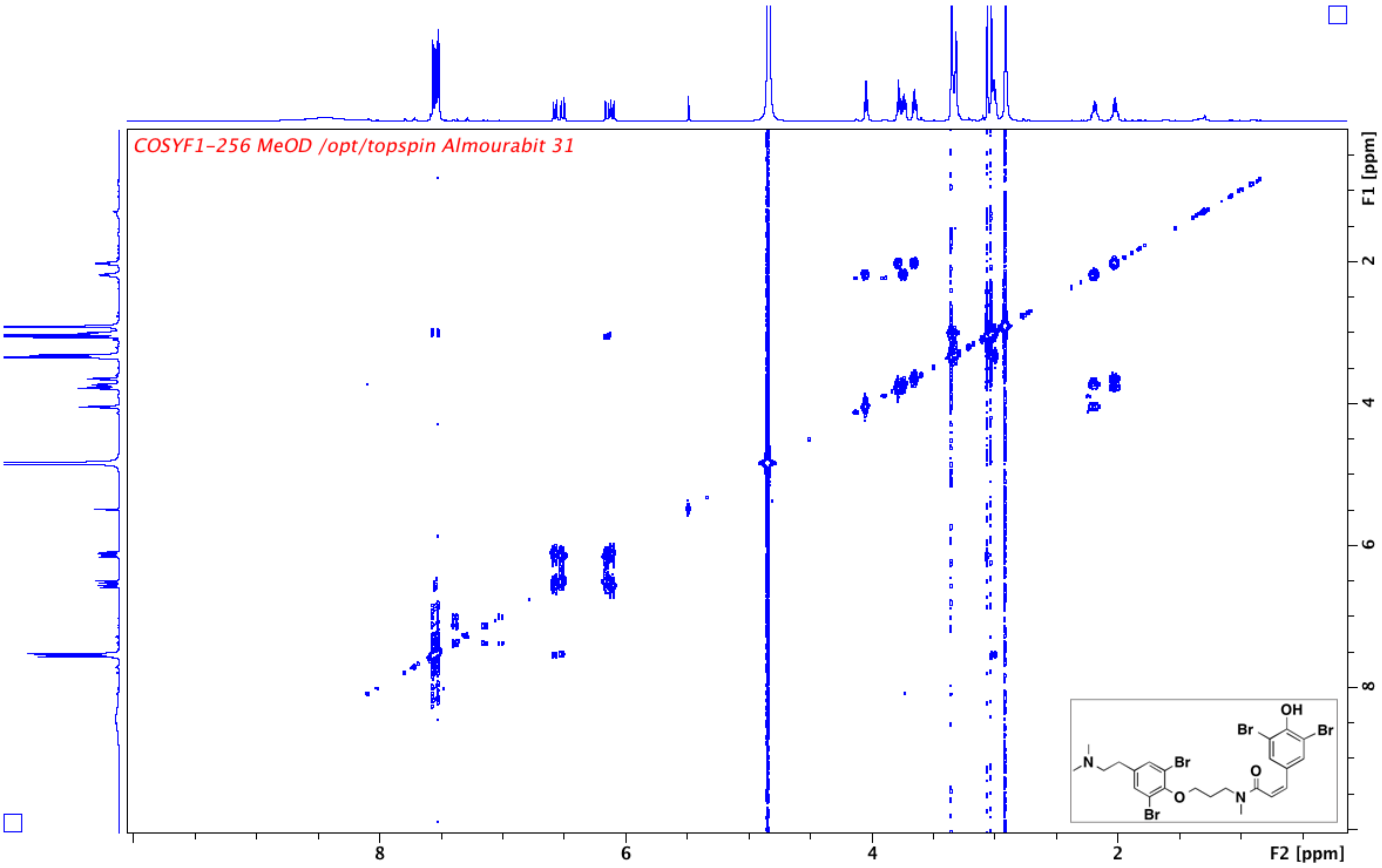

Figure S20. ^{1}H-^{13}C HMBC NMR spectrum of Psammaplysene G (**3**) in MeOD (500 MHz)

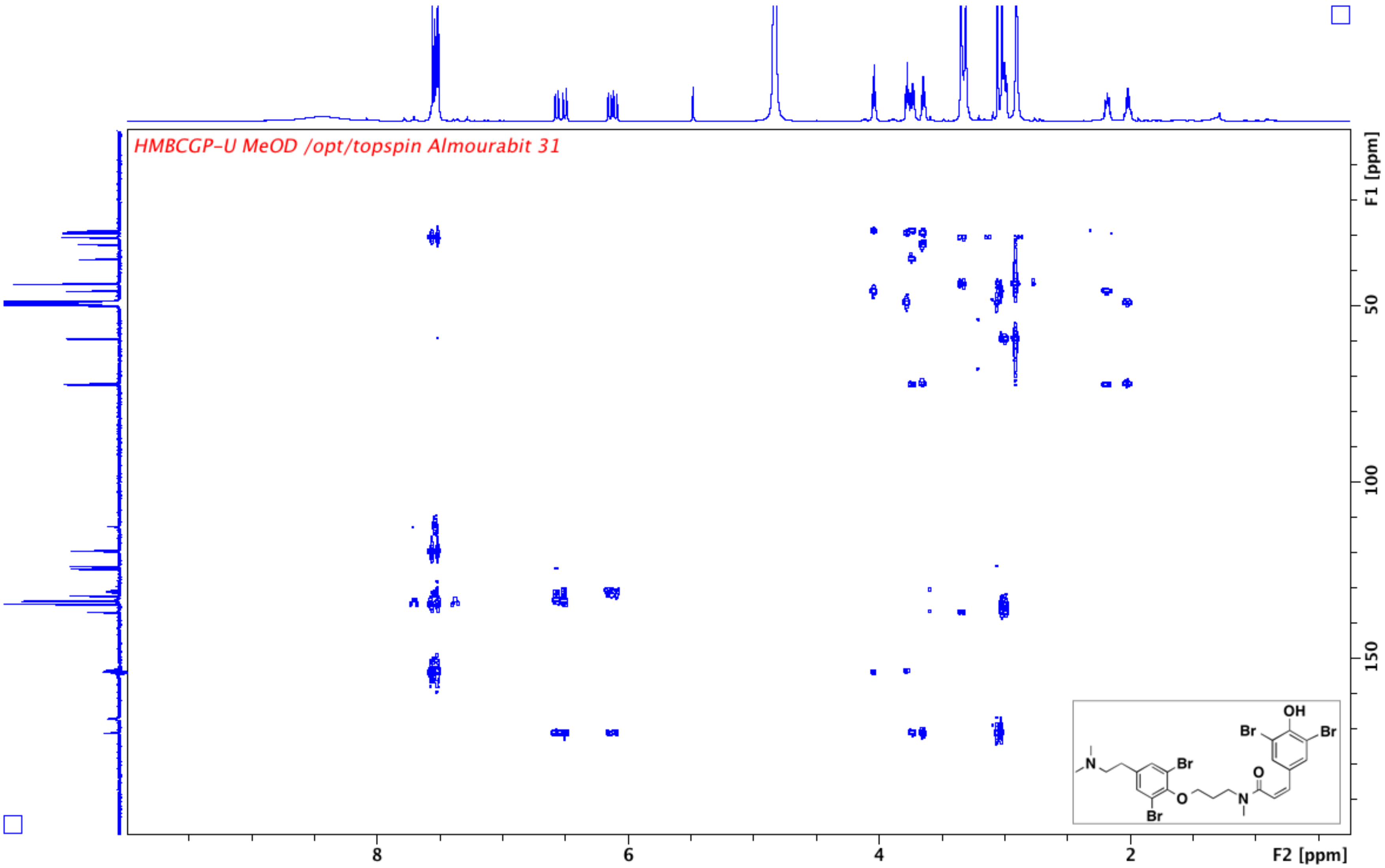

Figure S21. HR-ESI mass spectrum of Psammaplysene G (**3**)

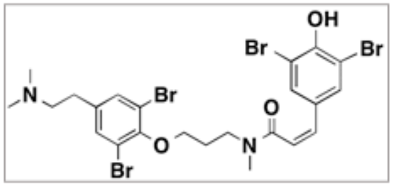

Elemental Composition Report

Single Mass Analysis

Tolerance = 5.0 PPM / DBE: min = -1.5, max = 100.0
Element prediction: Off
Number of isotope peaks used for i-FIT = 9

Monoisotopic Mass, Even Electron Ions
468 formula(e) evaluated with 3 results within limits (up to 50 best isotopic matches for each mass)
Elements Used:
C: 0-50 H: 0-100 N: 0-10 O: 0-10 79Br: 2-2 81Br: 2-2

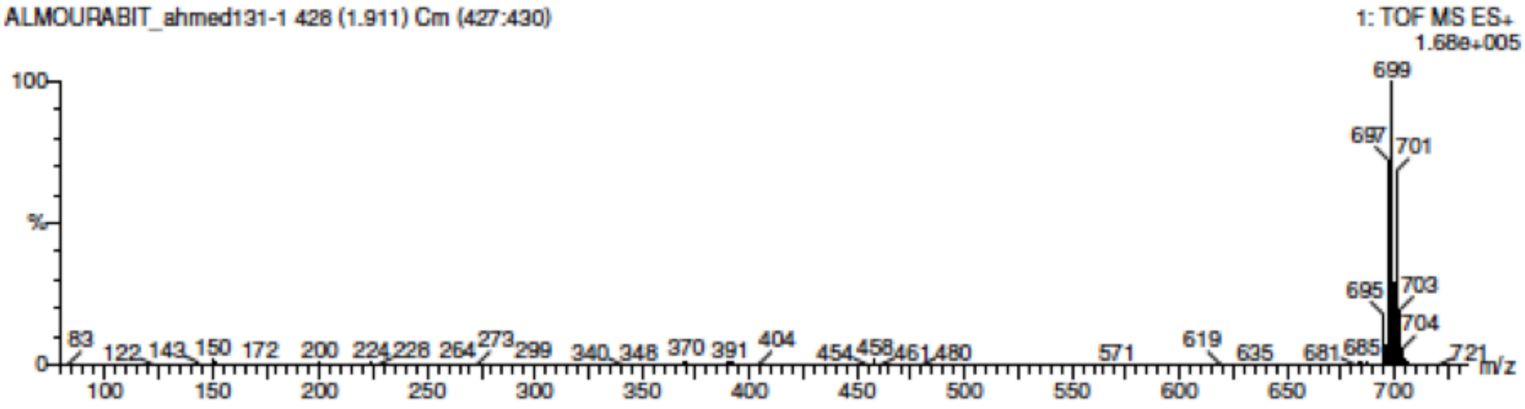

Minimum:				-1.5			
Maximum:		200.0	5.0	100.0			
Mass	Calc. Mass	mDa	PPM	DBE	i-FIT	i-FIT (Norm)	Formula
698.8723	698.8714	0.9	1.3	9.5	109.1	1.0	C23 H27 N2 O3 79Br2 81Br2
	698.8746	-2.3	-3.3	1.5	109.1	1.1	C12 H27 N8 O6 79Br2 81Br2
	698.8754	-3.1	-4.4	13.5	109.2	1.2	C28 H27 O 79Br2 81Br2

Figure S4. ^{1}H NMR spectrum of Psammaplysenes H (**4**) and I (**5**) in MeOD (500 MHz)

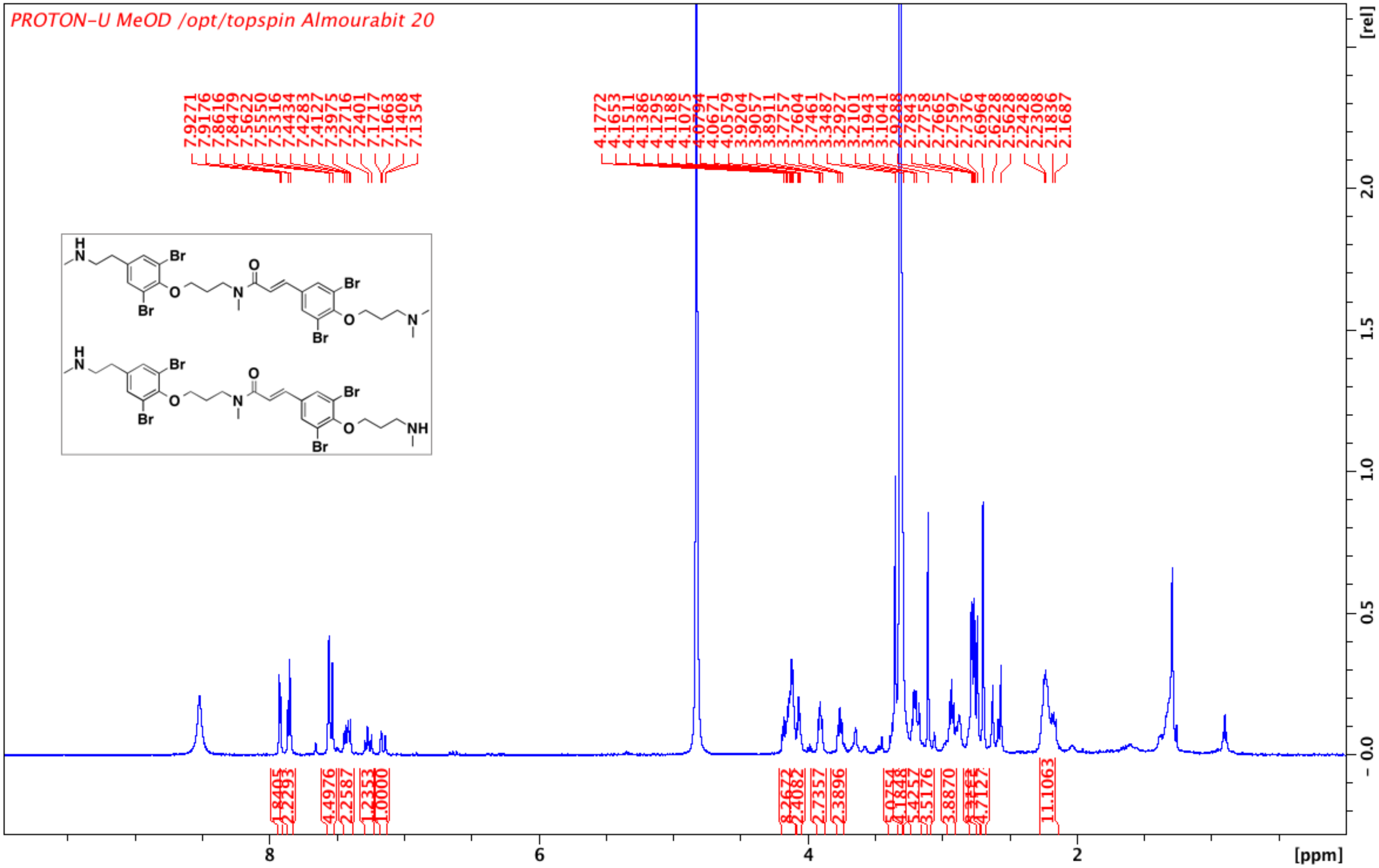

Figure S5. ^{13}C NMR spectrum of Psammaplysenes H (**4**) et I (**5**) in MeOD (500 MHz)

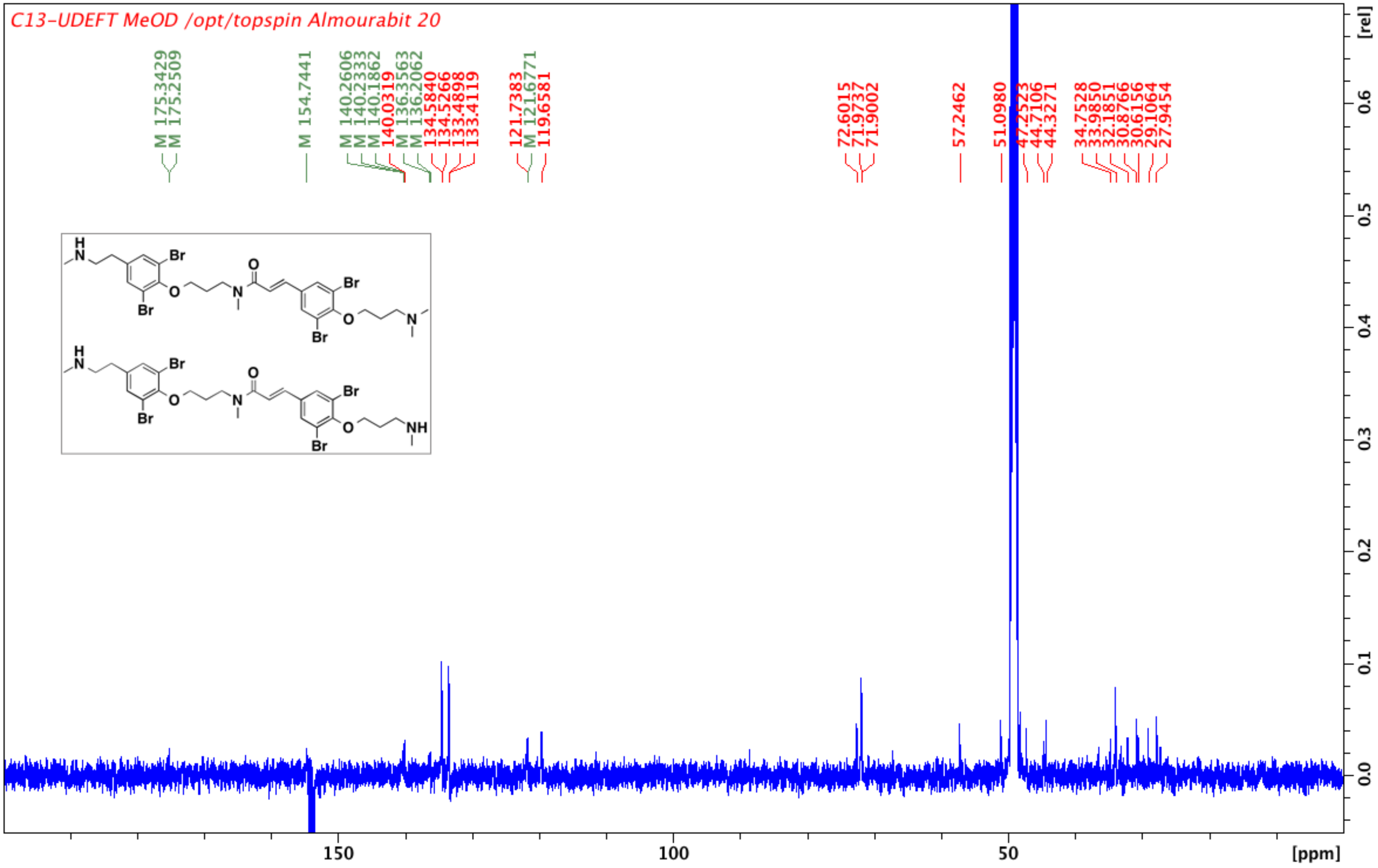

Figure S6. HSQC NMR spectrum of Psammaplysenes H (**4**) and I (**5**) in MeOD (500 MHz)

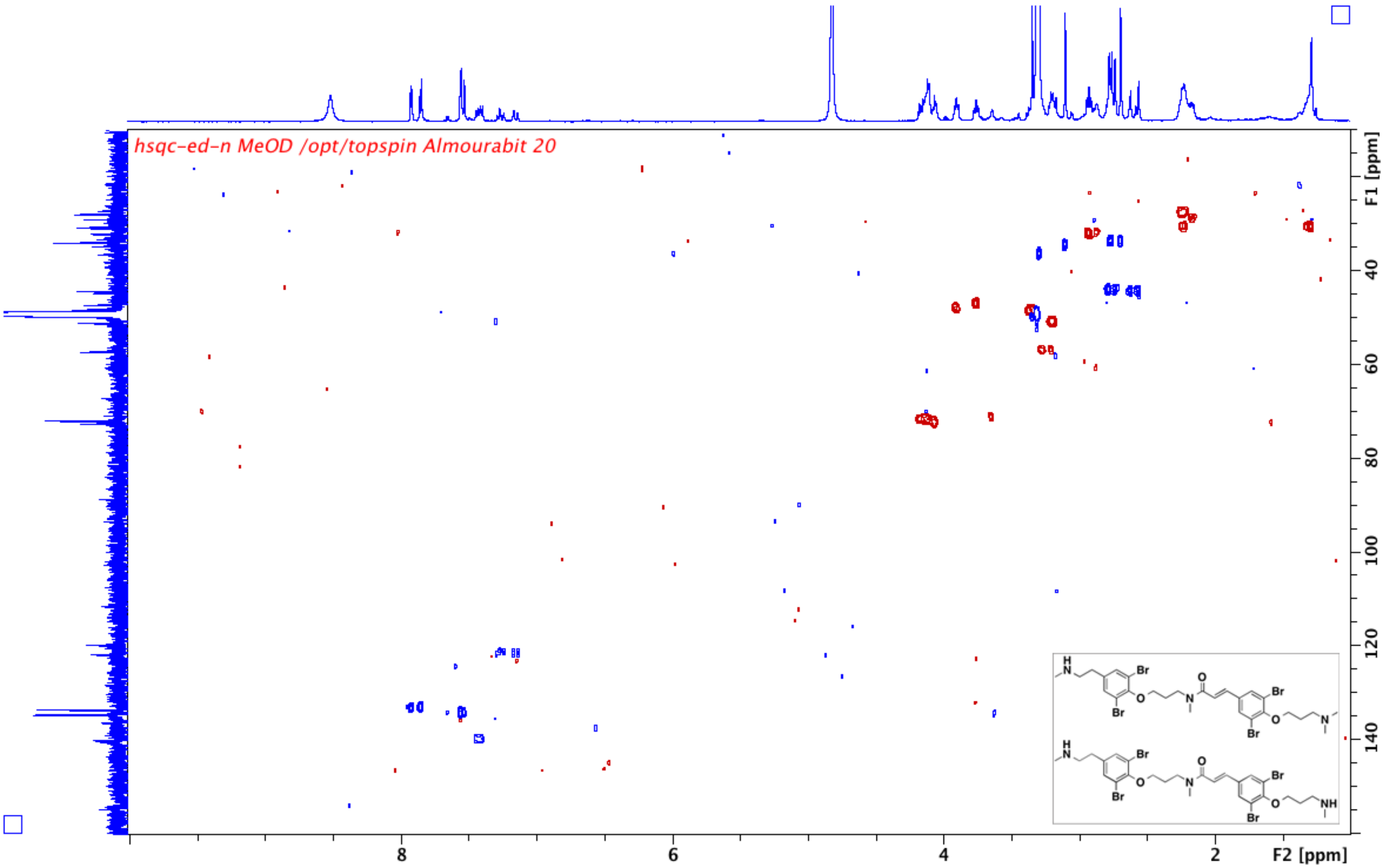

Figure S7. ^1H–^1H COSY NMR spectrum of Psammaplysenes H (**4**) and I (**5**) in MeOD (500 MHz)

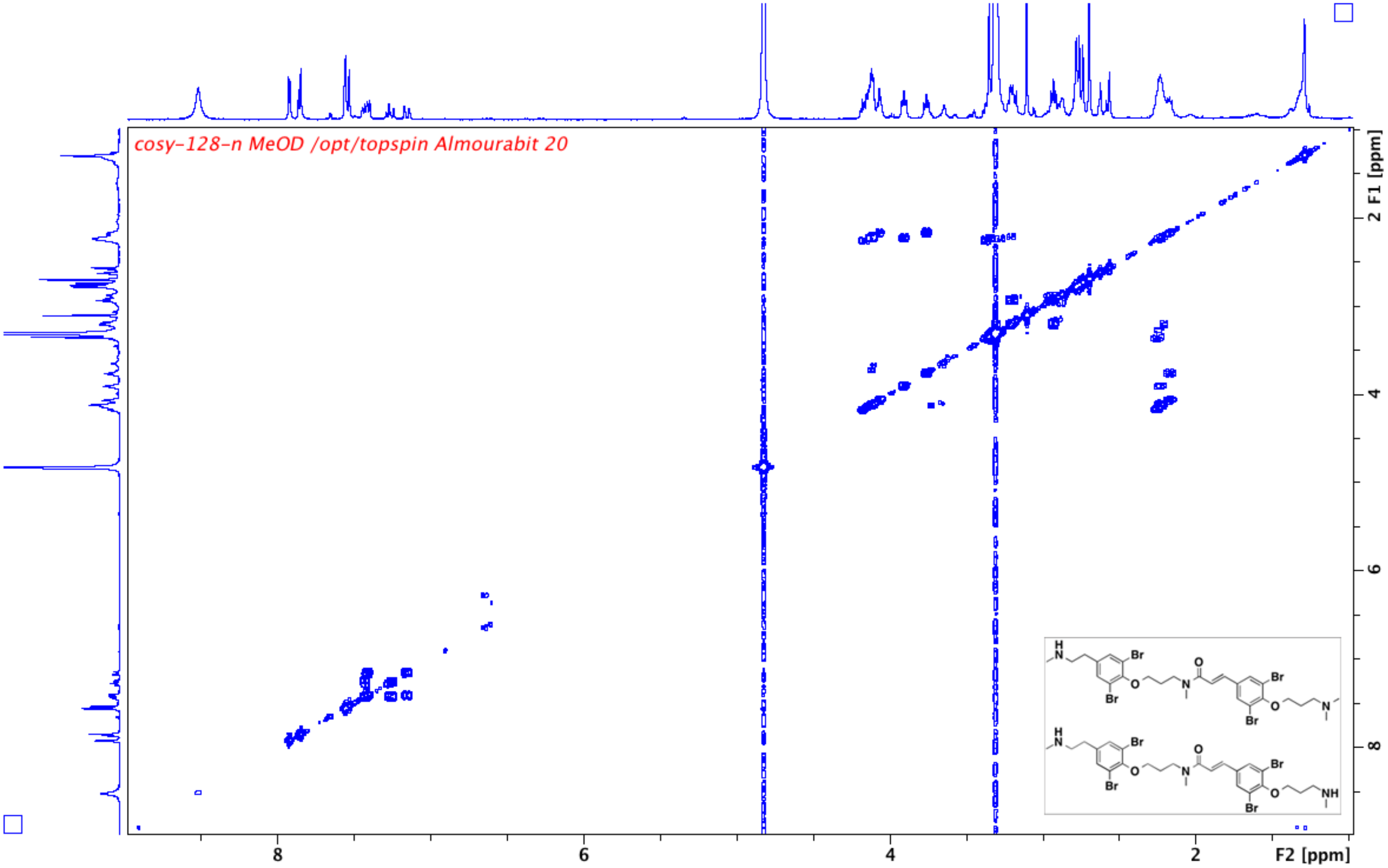

Figure S8. ^{1}H-^{13}C HMBC NMR spectrum of Psammaplysenes H (**4**) and I (**5**) in MeOD (500 MHz)

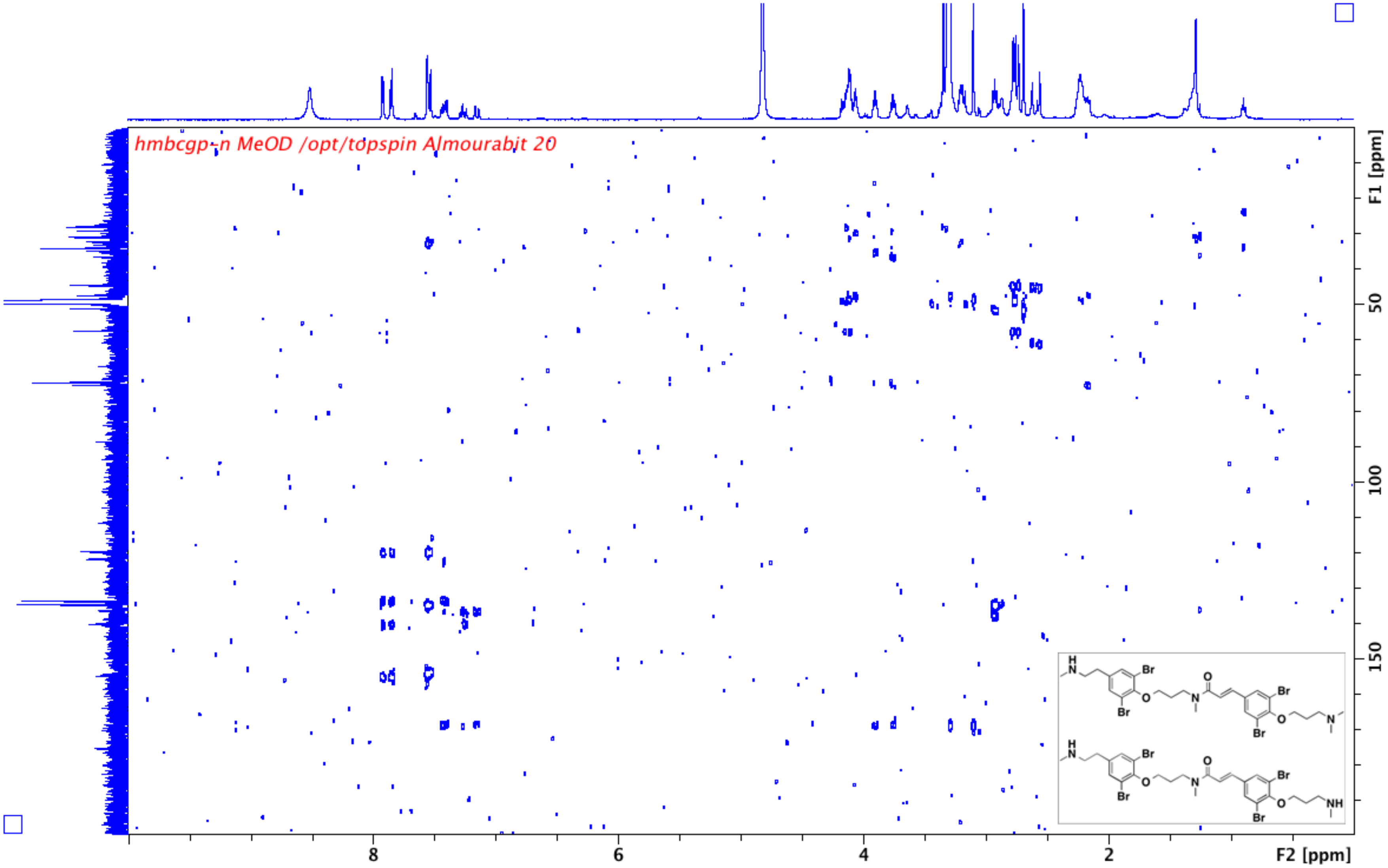

Figure S9. HR-ESI mass spectrum of Psammaplysenes H (**4**) and 12 I (**5**)

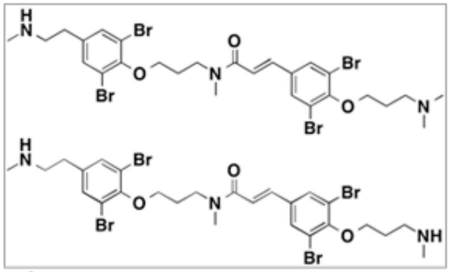

Elemental Composition Report

Single Mass Analysis
Tolerance = 10.0 PPM / DBE: min = -1.5, max = 100.0
Element prediction: Off
Number of isotope peaks used for i-FIT = 9

Monoisotopic Mass, Even Electron Ions
557 formula(e) evaluated with 7 results within limits (up to 50 best isotopic matches for each mass)
Elements Used:
C: 0-50 H: 0-100 N: 0-10 O: 0-10 79Br: 2-2 81Br: 2-2

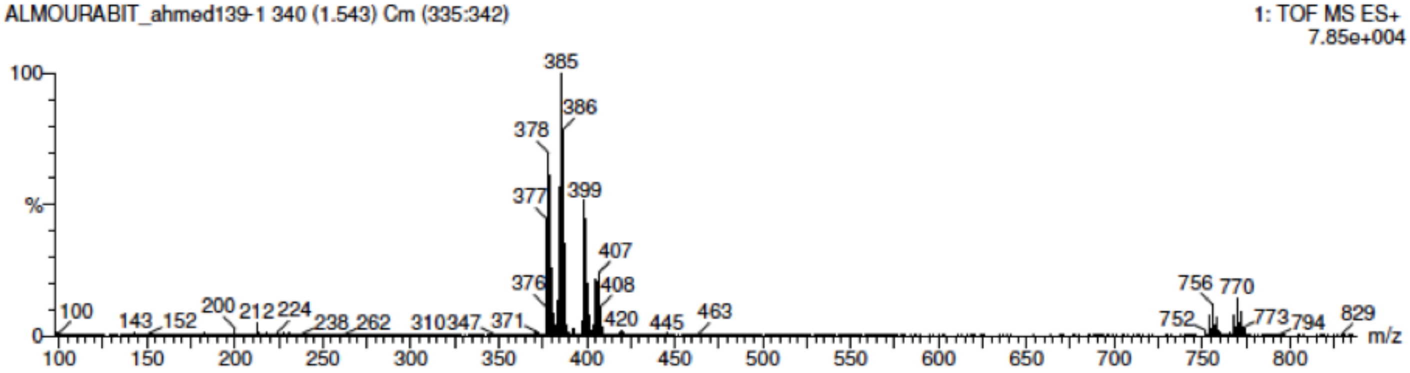

Minimum:				-1.5			
Maximum:		200.0	10.0	100.0			
Mass	Calc. Mass	mDa	PPM	DBE	i-FIT	i-FIT (Norm)	Formula
769.9521	769.9489	3.2	4.2	13.5	83.3	1.8	C32 H36 N O 79Br2 81Br2
	769.9548	-2.7	-3.5	4.5	83.3	1.9	C25 H40 N O6 79Br2 81Br2
	769.9449	7.2	9.4	9.5	83.4	1.9	C27 H36 N3 O3 79Br2 81Br2
	769.9562	-4.1	-5.3	9.5	83.4	1.9	C26 H36 N5 O2 79Br2 81Br2

Figure S22. ^{1}H NMR spectrum of Anomoian C (**6**) in MeOD (500 MHz)

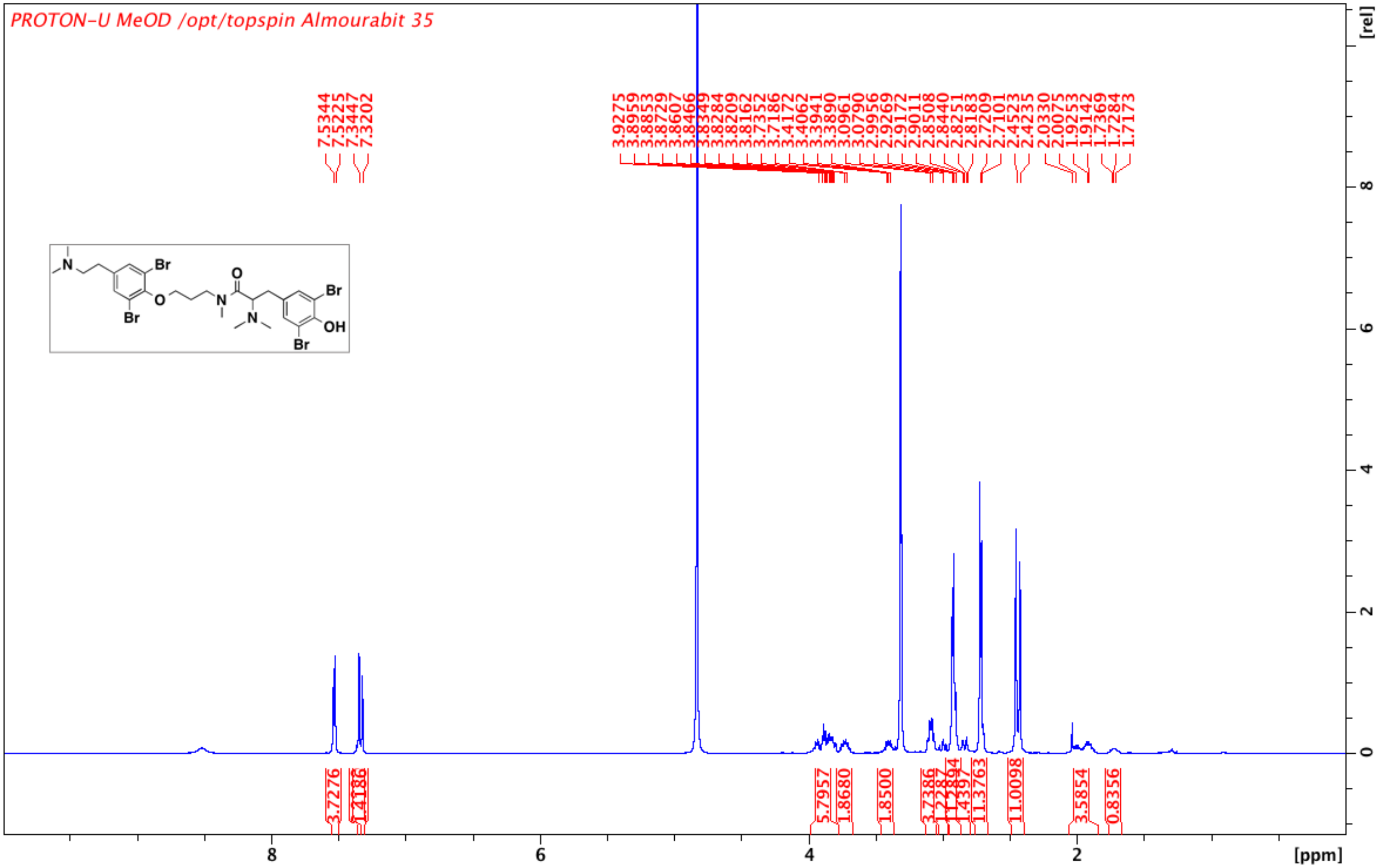

Figure S23. ^{13}C NMR spectrum of Anomoian C (**6**) in MeOD (500 MHz)

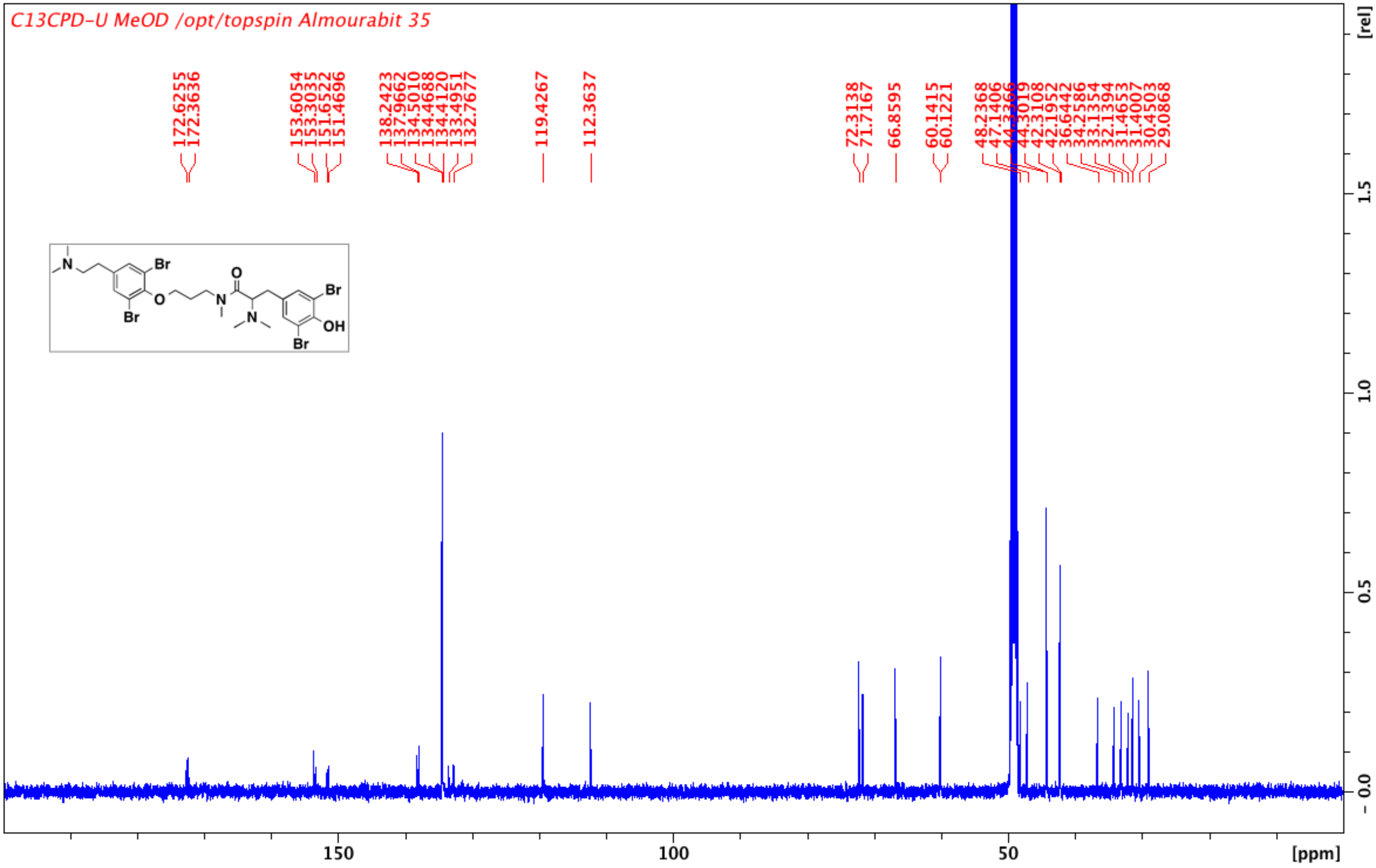

Figure S24. HSQC NMR spectrum of Anomoian C (**6**) in MeOD (500 MHz)

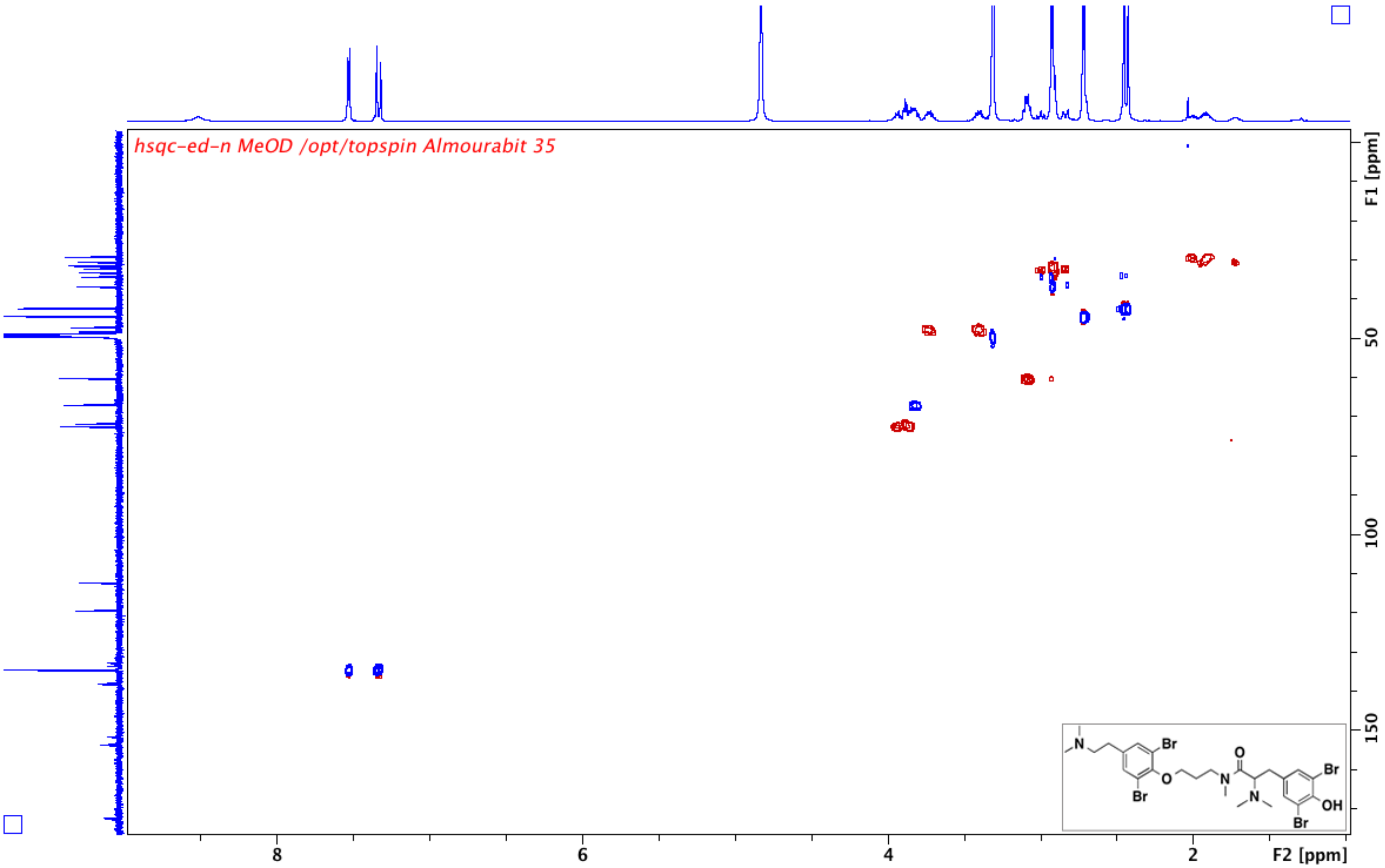

Figure S25. ¹H–¹H COSY NMR spectrum of Anomoian C (**6**) in MeOD (500 MHz)

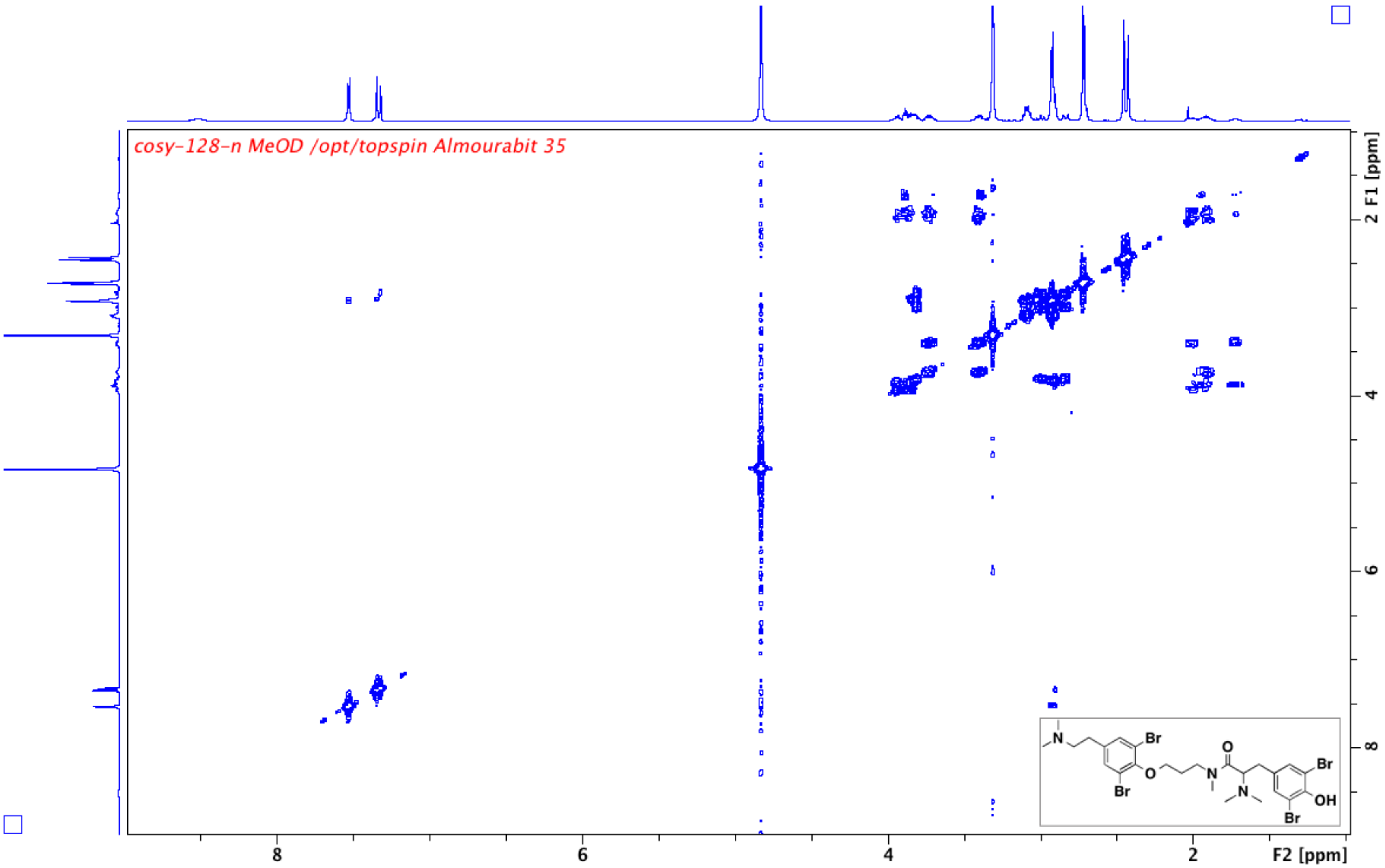

Figure S26. ^{1}H-^{13}C HMBC NMR spectrum of Anomoian C (**6**) in MeOD (500 MHz)

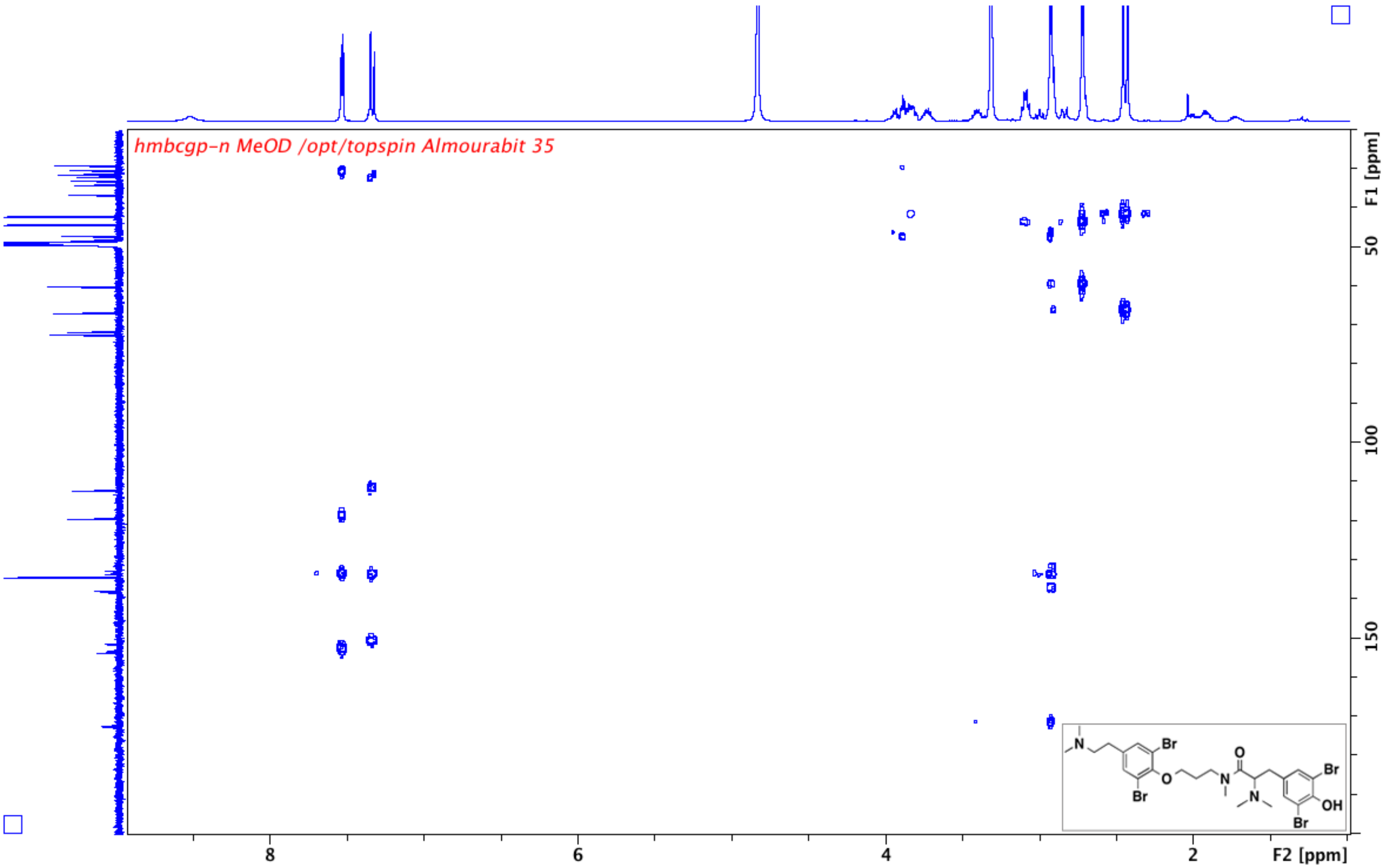

Figure S27. HR-ESI mass spectrum of Anomoian C (**6**)

Elemental Composition Report

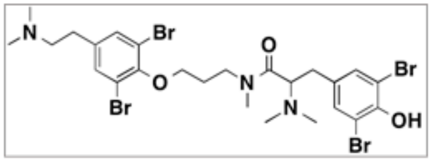

Single Mass Analysis

Tolerance = 13.0 PPM / DBE: min = -1.5, max = 100.0
Element prediction: Off
Number of isotope peaks used for i-FIT = 9

Monoisotopic Mass, Even Electron Ions
517 formula(e) evaluated with 10 results within limits (up to 50 best isotopic matches for each mass)
Elements Used:
C: 0-50 H: 0-100 N: 0-10 O: 0-10 79Br: 2-2 81Br: 2-2

ALMOURABIT_ahmed147-1 287 (1.322) Cm (285:290)

1: TOF MS ES+
1.82e+005

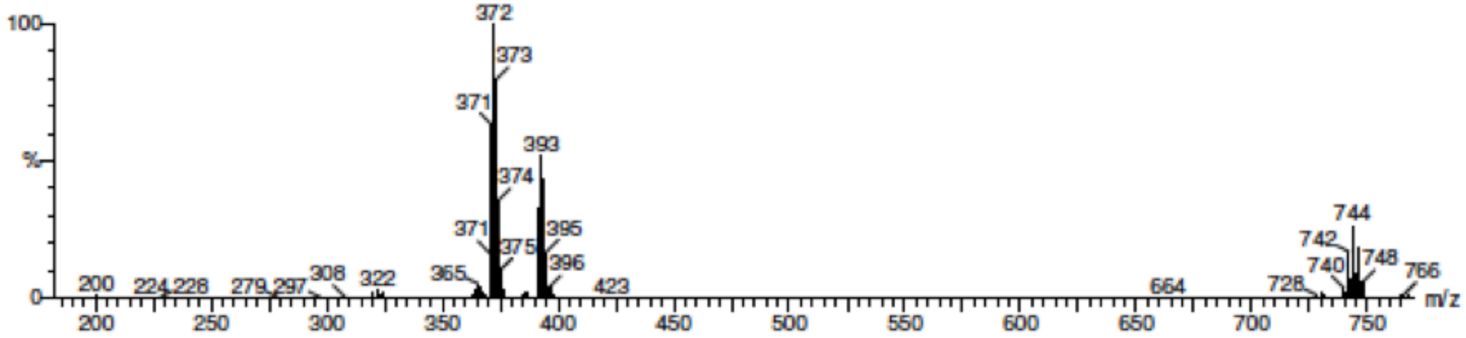

Minimum:				-1.5			
Maximum:		200.0	13.0	100.0			
Mass	Calc. Mass	mDa	PPM	DBE	i-FIT	i-FIT (Norm)	Formula
743.9386	743.9293	9.3	12.5	8.5	116.5	2.2	C25 H34 N3 O3 79Br2 81Br2
	743.9392	-0.6	-0.8	3.5	116.6	2.3	C23 H38 N O6 79Br2 81Br2
	743.9405	-1.9	-2.6	8.5	116.6	2.3	C24 H34 N5 O2 79Br2 81Br2

Figure S28. ^{1}H NMR spectrum of Anomoian D (**7**) in MeOD (500 MHz)

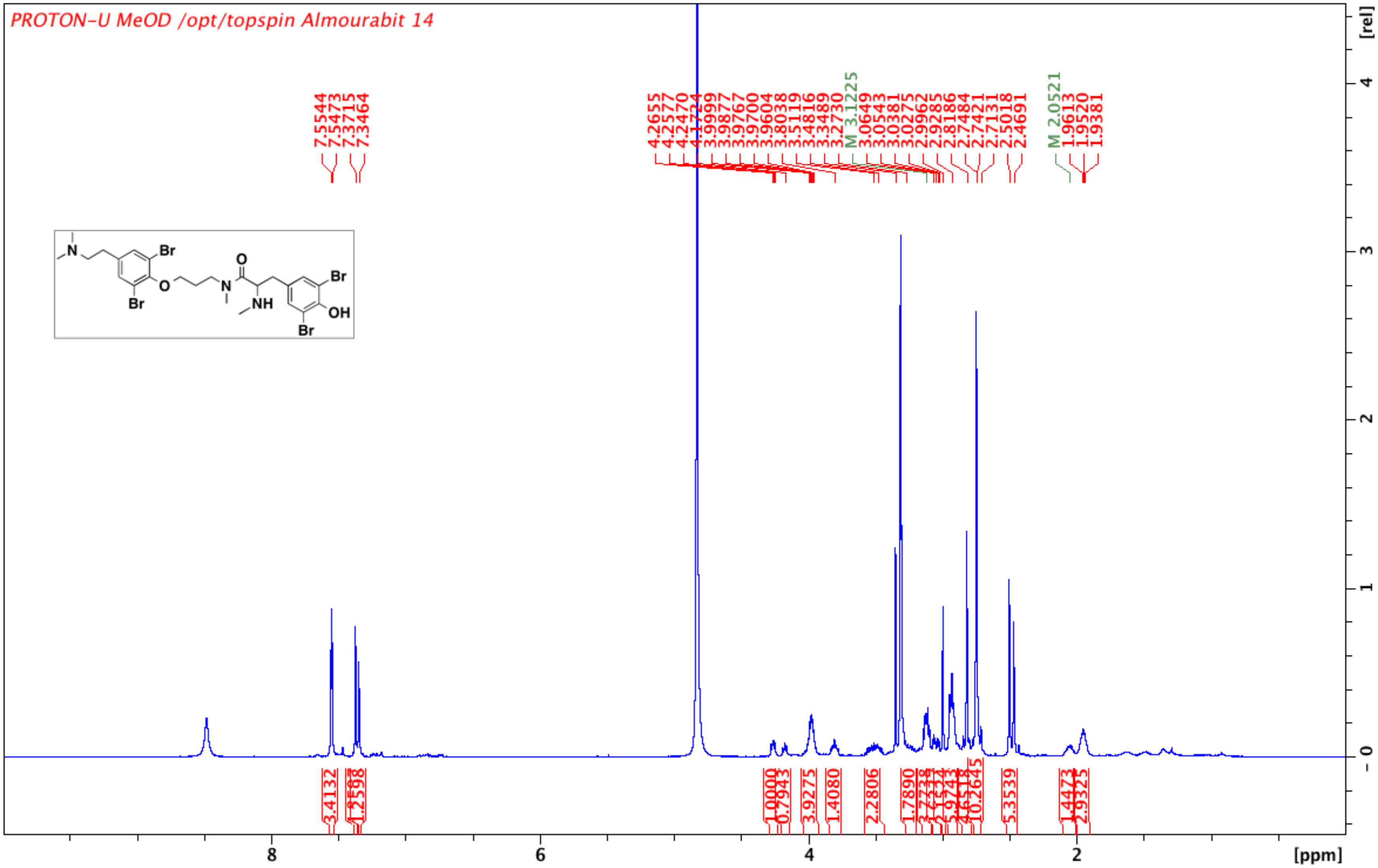

Figure S29. ^{13}C NMR spectrum of Anomoian D (**7**) in MeOD (500 MHz)

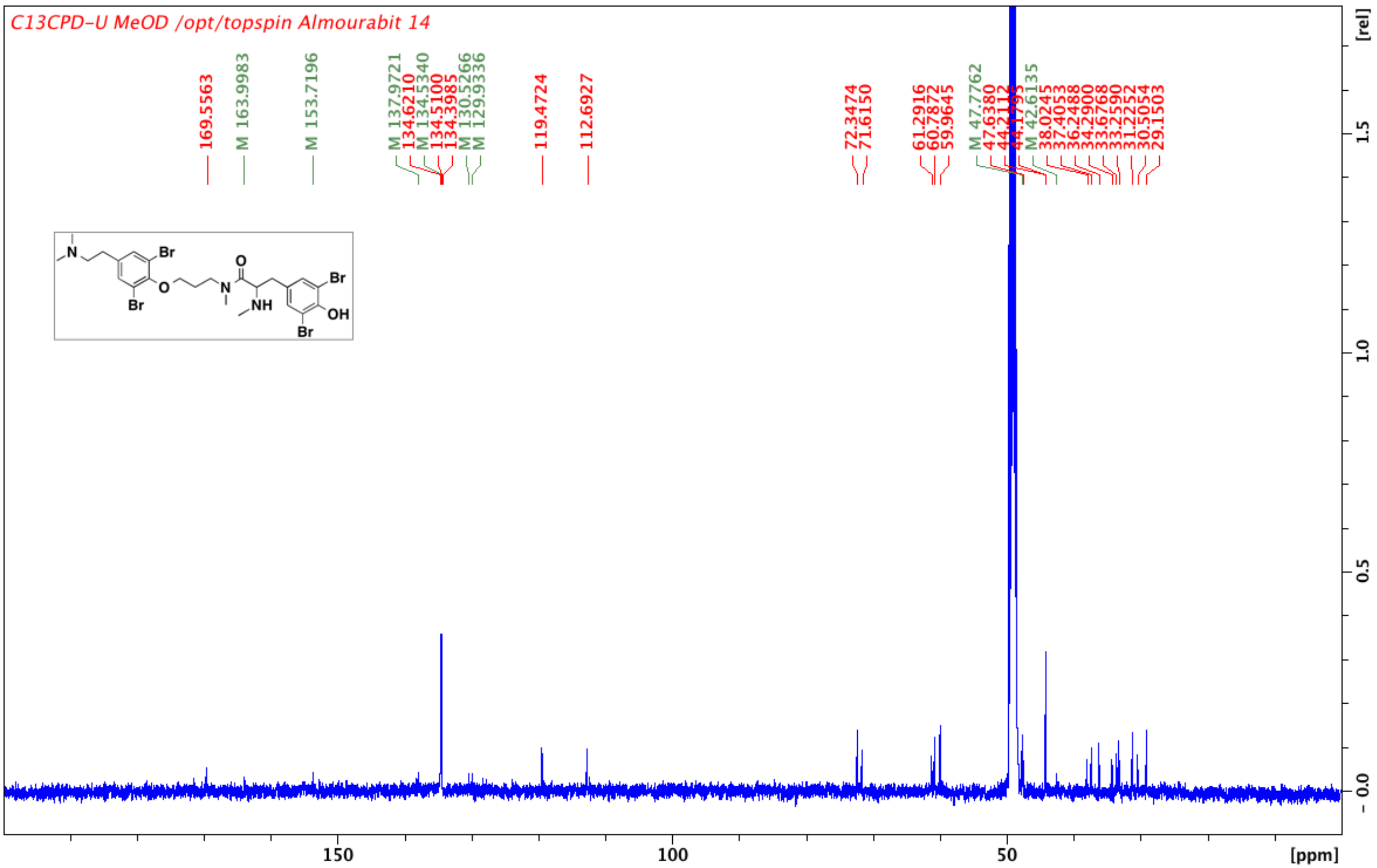

Figure S30. HSQC NMR spectrum of Anomoian D (**7**) in MeOD (500 MHz)

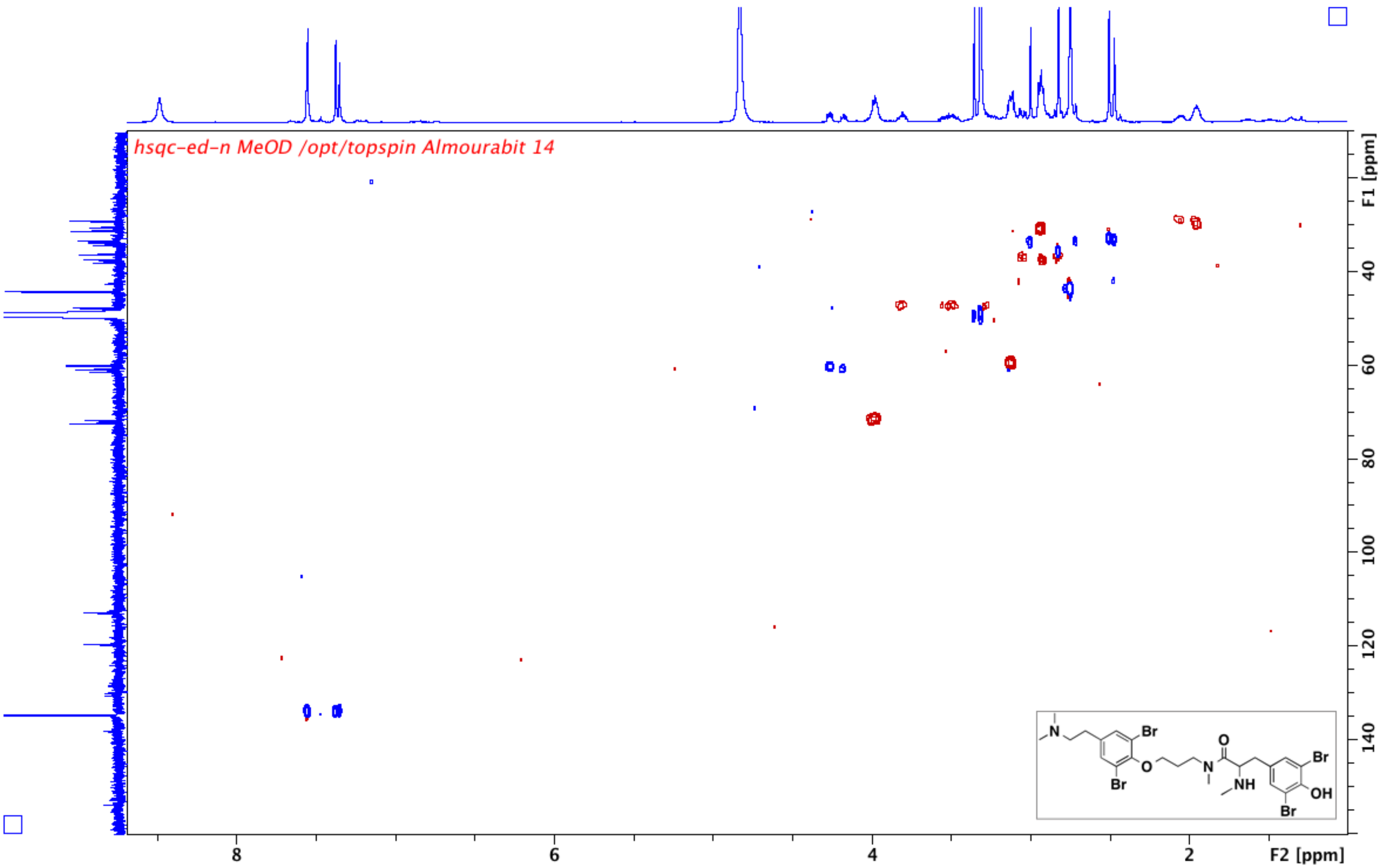

Figure S31. ¹H–¹H COSY NMR spectrum of Anomoian D (**7**) in MeOD (500 MHz)

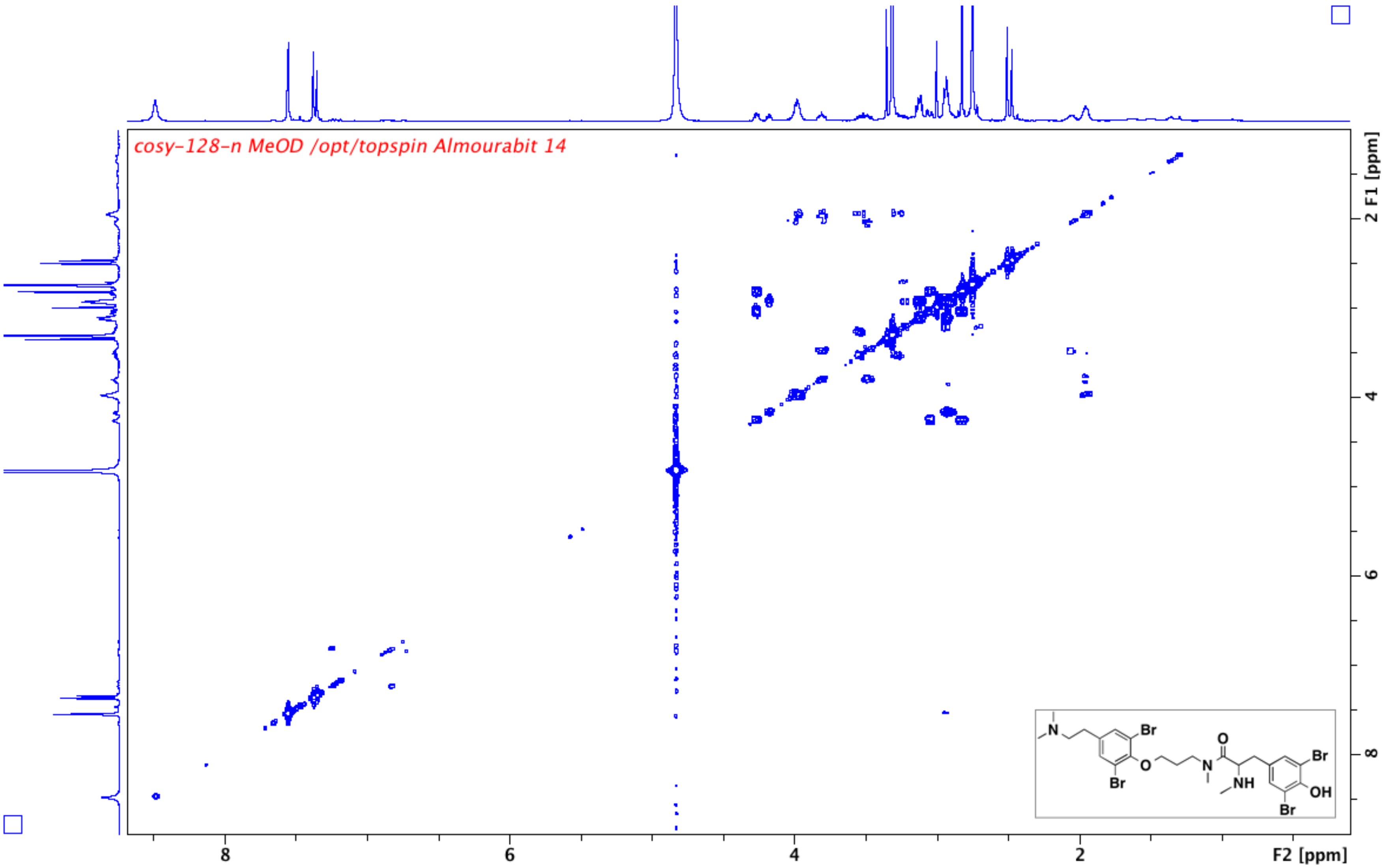

Figure S32. ^{1}H-^{13}C HMBC NMR spectrum of Anomoian D (**7**) in MeOD (500 MHz)

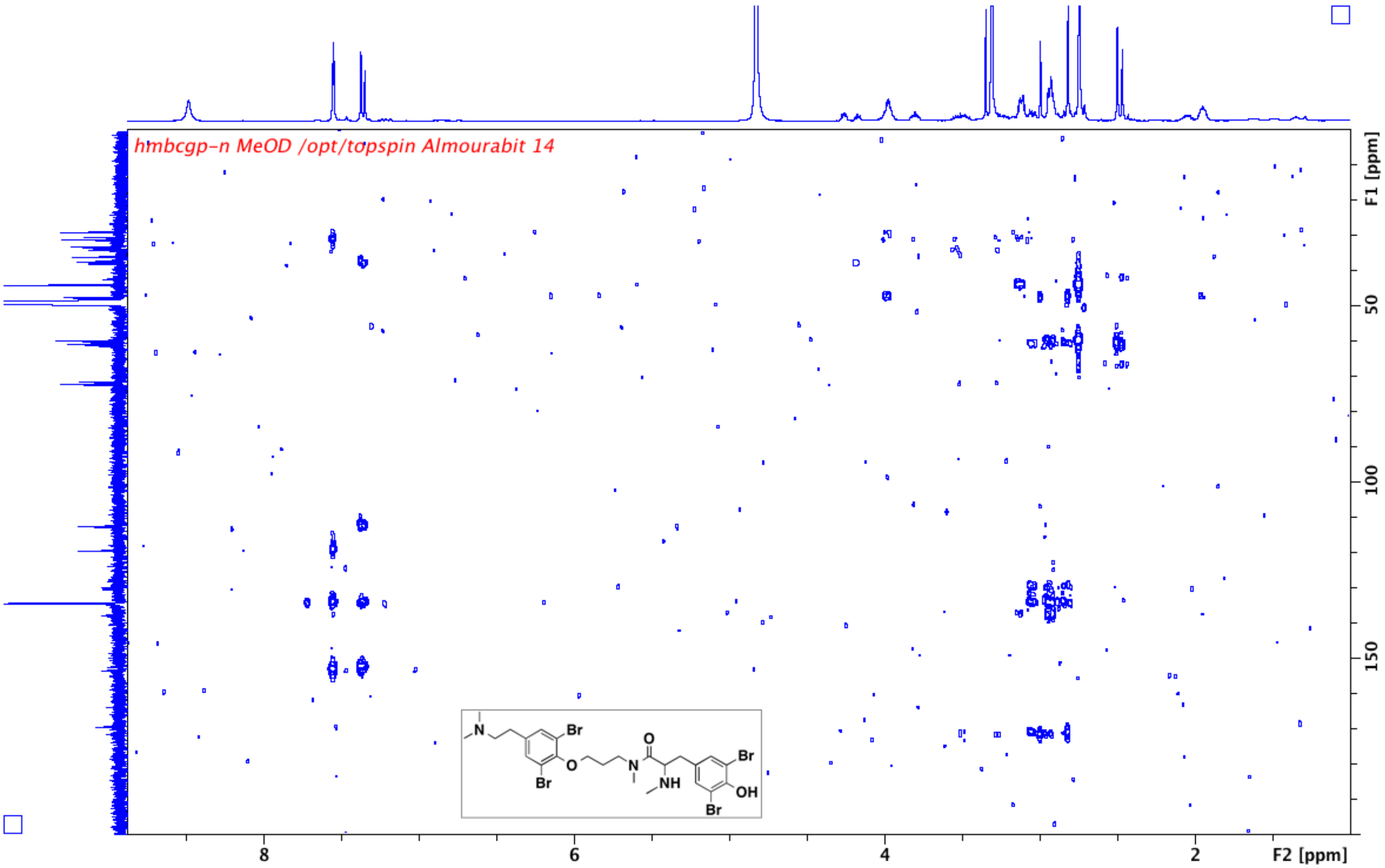

Figure S33. HR-ESI mass spectrum of Anomoian D (**7**)

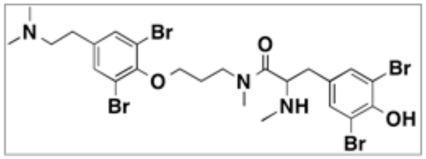

Elemental Composition Report

Single Mass Analysis

Tolerance = 8.0 PPM / DBE: min = -1.5, max = 100.0
Element prediction: Off
Number of isotope peaks used for i-FIT = 9

Monoisotopic Mass, Even Electron Ions
495 formula(e) evaluated with 6 results within limits (up to 50 best isotopic matches for each mass)
Elements Used:
C: 0-50 H: 0-100 N: 0-10 O: 0-10 79Br: 2-2 81Br: 2-2

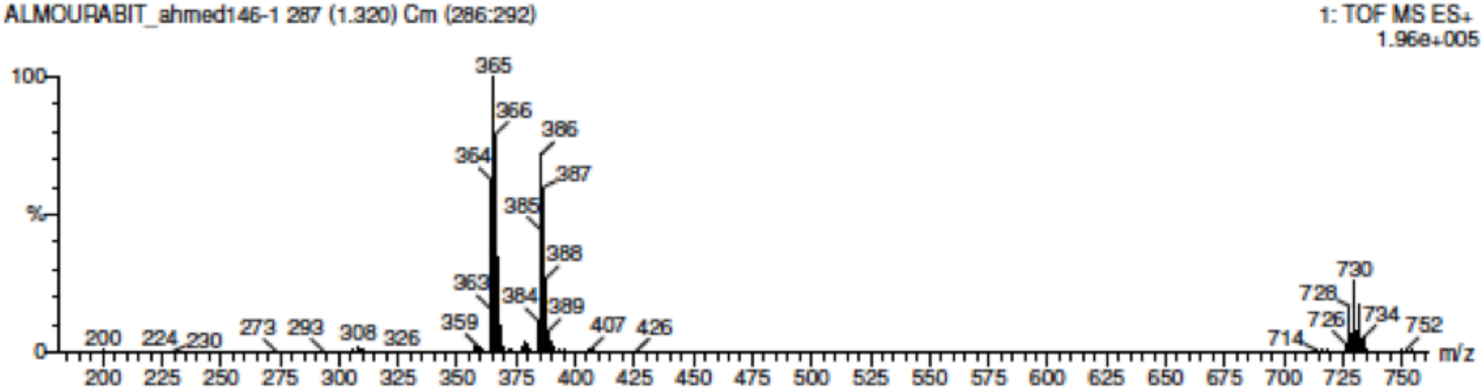

Minimum:				-1.5			
Maximum:		200.0	8.0	100.0			
Mass	Calc. Mass	mDa	PPM	DBE	i-FIT	i-FIT (Norm)	Formula
729.9190	729.9136	5.4	7.4	8.5	124.1	1.7	C24 H32 N3 O3 79Br2 81Br2
	729.9208	-1.8	-2.5	4.5	124.2	1.8	C18 H32 N7 O4 79Br2 81Br2
	729.9235	-4.5	-6.2	3.5	124.2	1.8	C22 H36 N O6 79Br2 81Br2

Figure S34. ^{1}H NMR spectrum of Anomoian E (**8**) in MeOD (500 MHz)

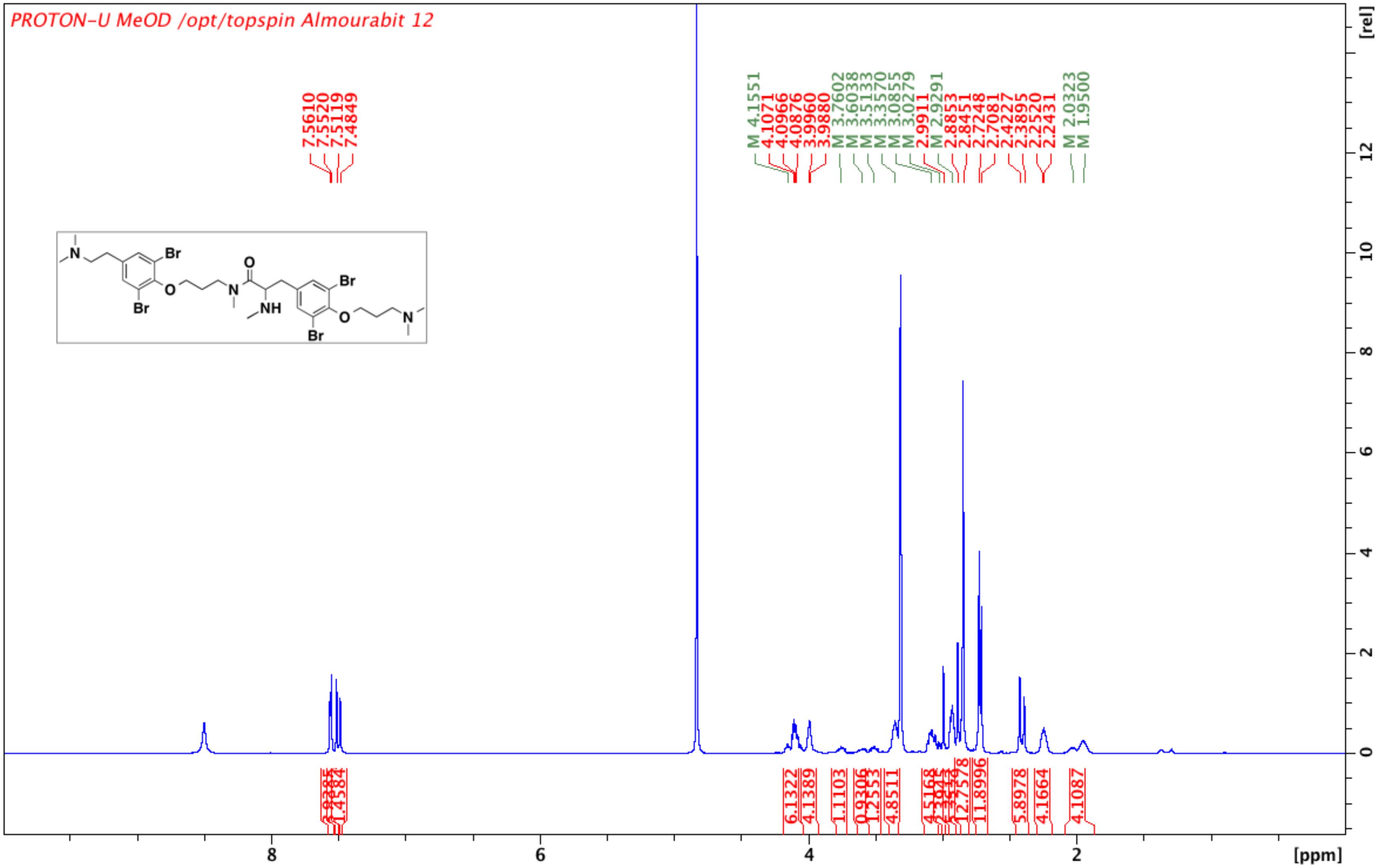

Figure S35. ^{13}C NMR spectrum of Anomoian E (**8**) in MeOD (500 MHz)

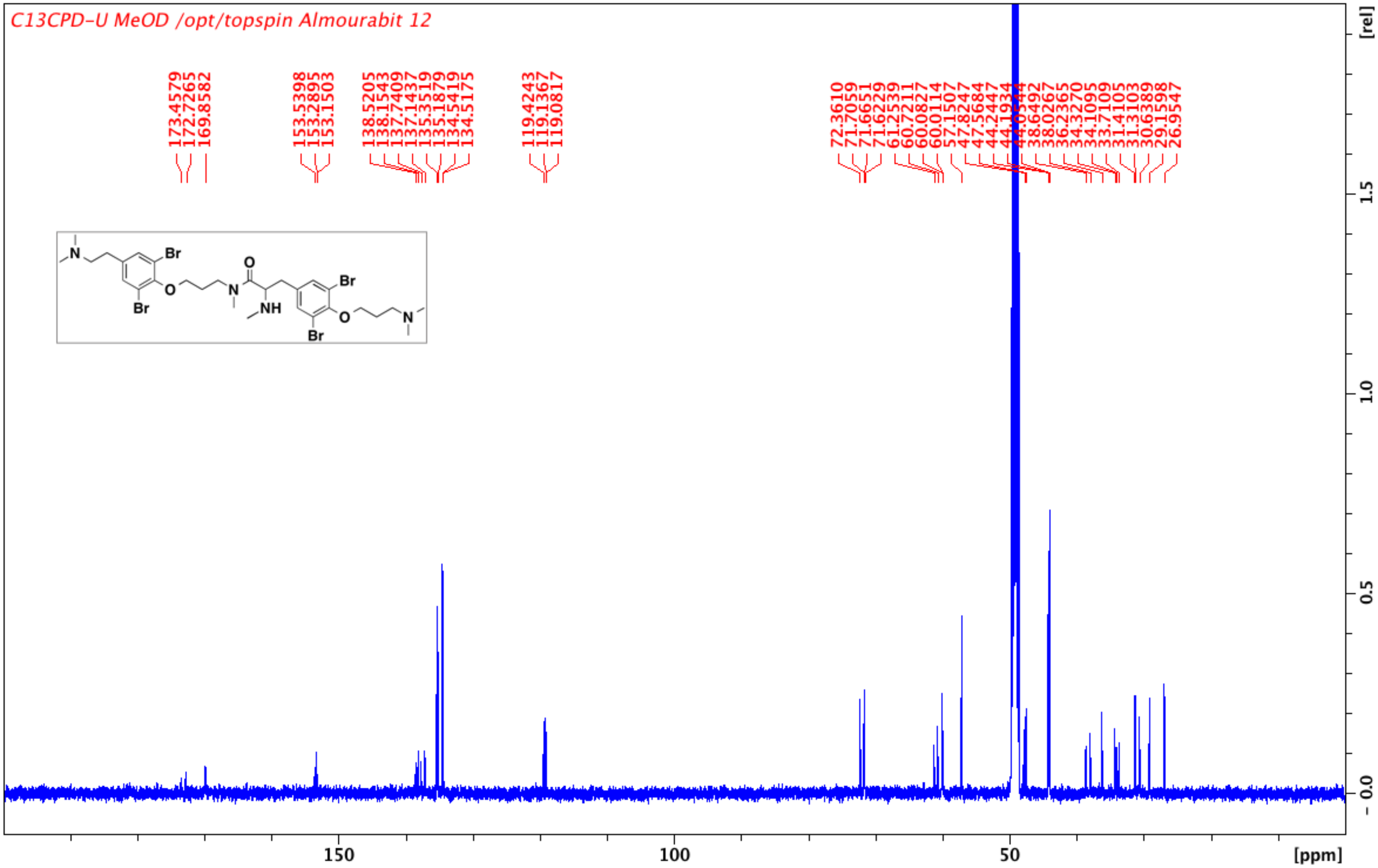

Figure S36. HSQC NMR spectrum of Anomoian E (**8**) in MeOD (500 MHz)

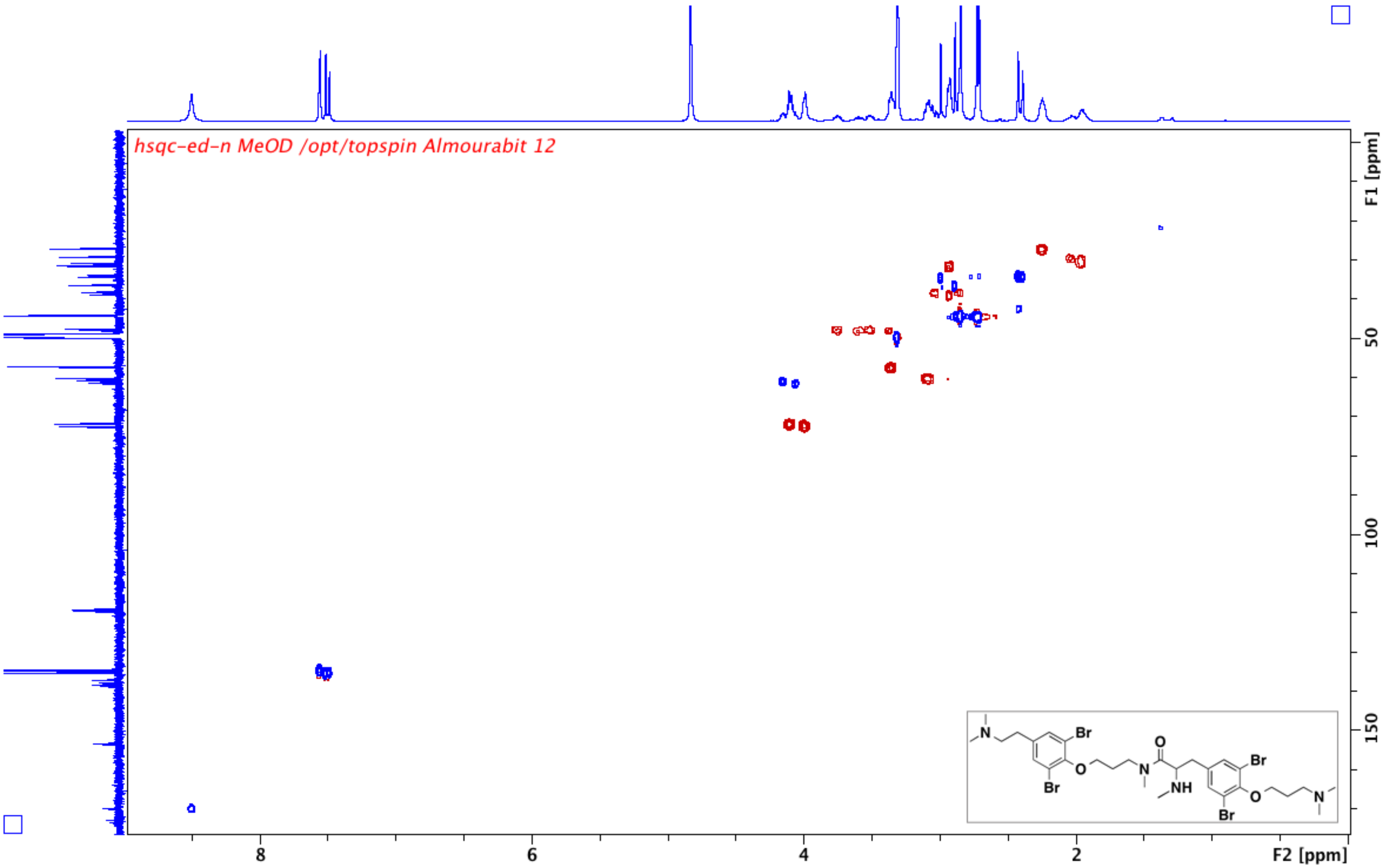

Figure S37. ^{1}H–^{1}H COSY NMR spectrum of Anomoian E (**8**) in MeOD (500 MHz)

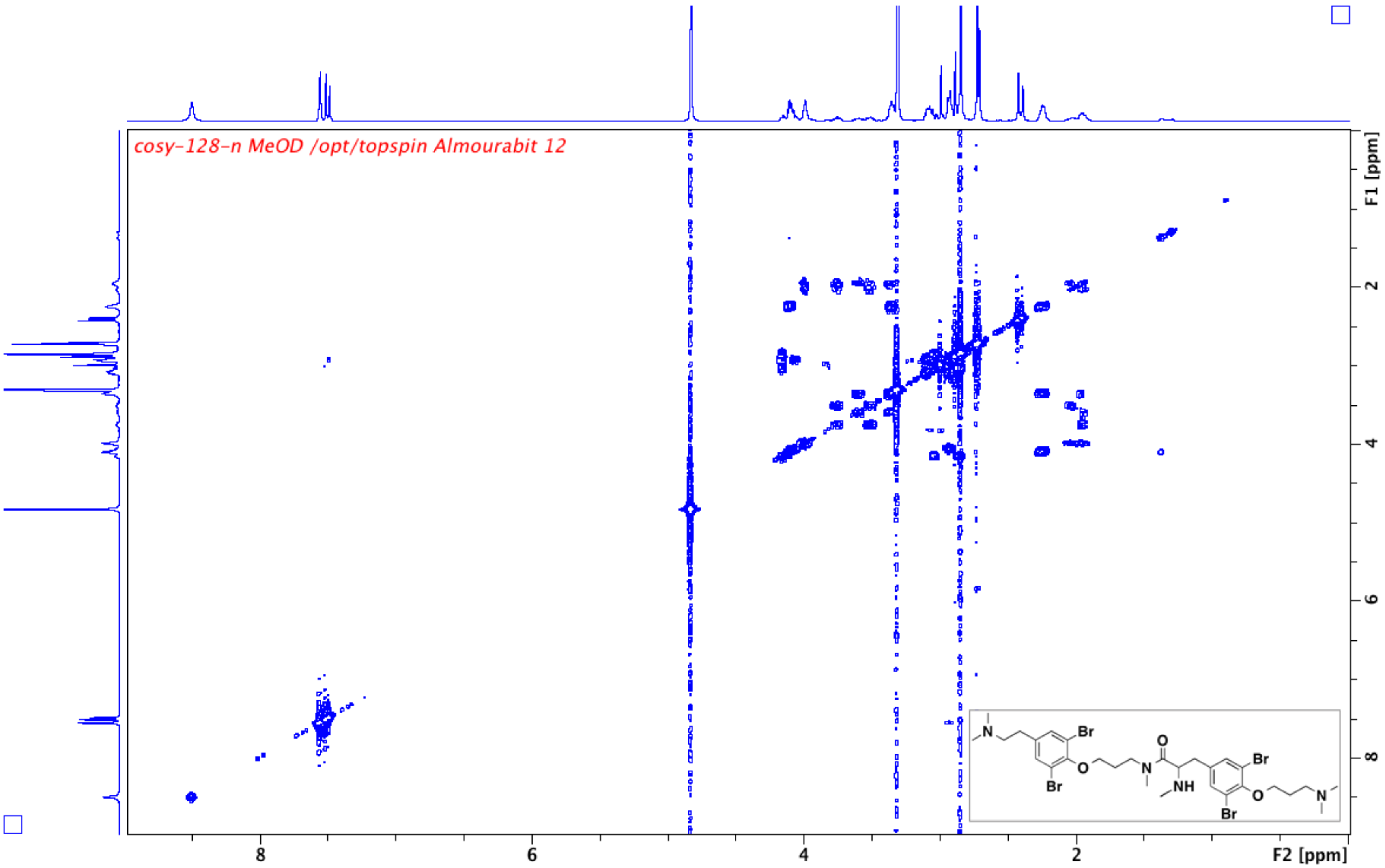

Figure S38. ^{1}H-^{13}C HMBC NMR spectrum of Anomoian E (**8**) in MeOD (500 MHz)

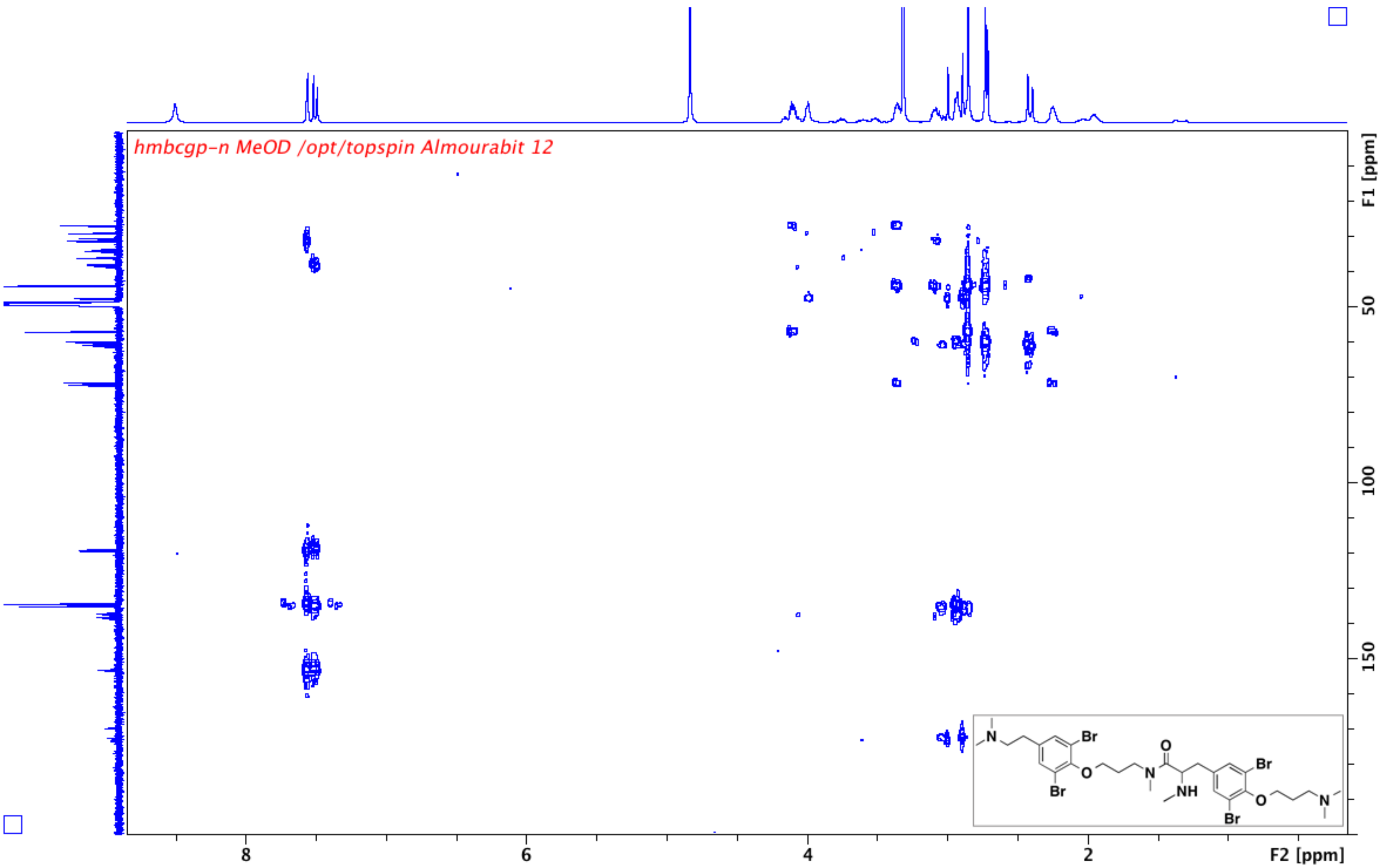

Figure S39. HR-ESI mass spectrum of Anomoian E (**8**)

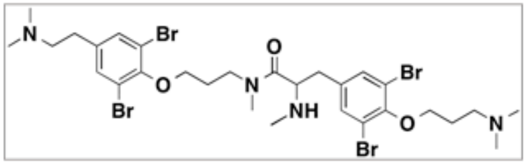

Elemental Composition Report

Single Mass Analysis

Tolerance = 5.0 PPM / DBE: min = -1.5, max = 100.0
Element prediction: Off
Number of isotope peaks used for i-FIT = 9

Monoisotopic Mass, Even Electron Ions
629 formula(e) evaluated with 5 results within limits (up to 50 best isotopic matches for each mass)
Elements Used:
C: 0-50 H: 0-100 N: 0-10 O: 0-10 79Br: 2-2 81Br: 2-2

ALMOURABIT_ahmed143-1 226 (1.056) Cm (225:228) 1: TOF MS ES+ 9.65e+004

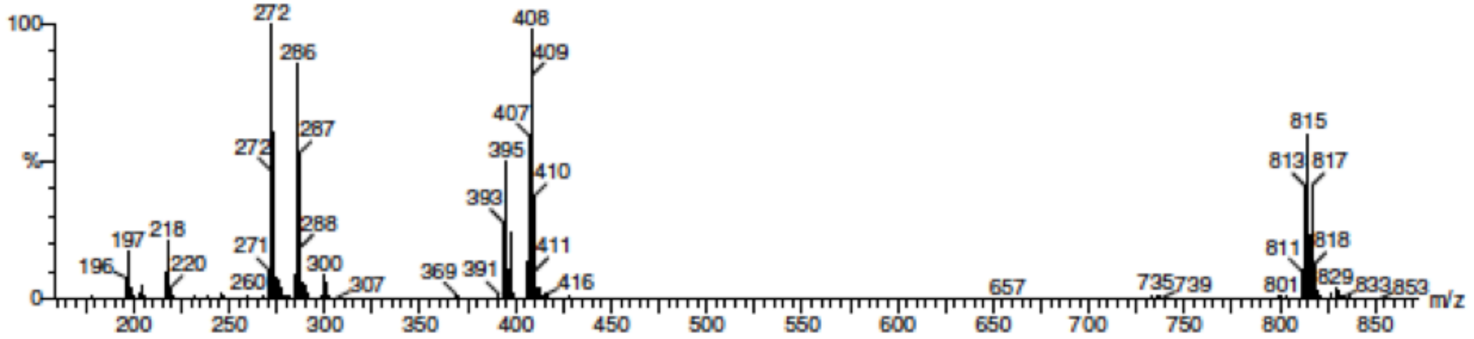

Minimum:				-1.5			
Maximum:		200.0	5.0	100.0			
Mass	Calc. Mass	mDa	PPM	DBE	i-FIT	i-FIT (Norm)	Formula
815.0068	815.0028	4.0	4.9	8.5	97.0	1.5	C29 H43 N4 O3 79Br2 81Br2
	815.0060	0.8	1.0	0.5	97.1	1.6	C18 H43 N10 O6 79Br2 81Br2
	815.0068	0.0	0.0	12.5	97.1	1.6	C34 H43 N2 O 79Br2 81Br2

Figure S40. ^{1}H NMR spectrum of Anomoian F (**9**) in MeOD (500 MHz)

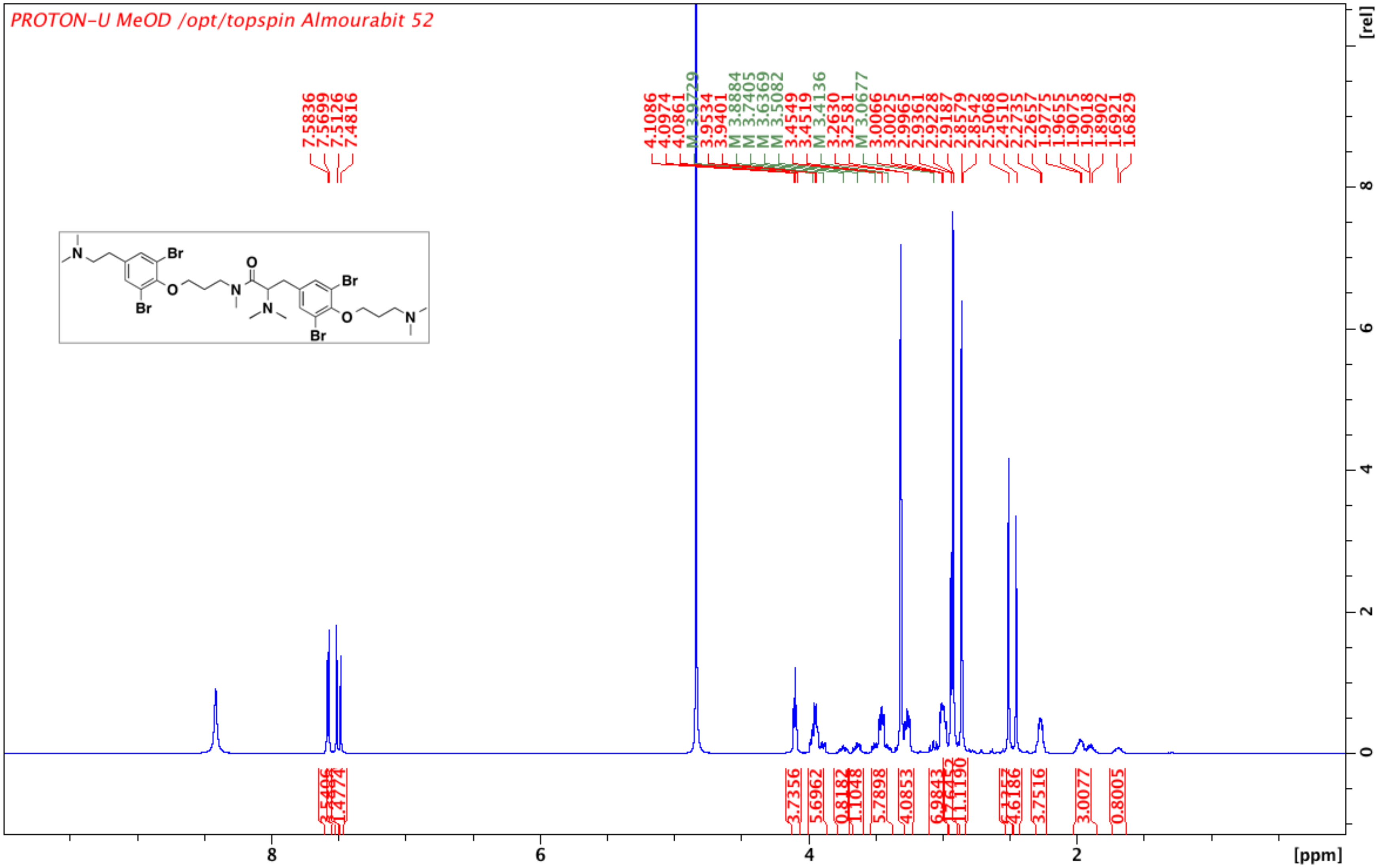

Figure S41. ^{13}C NMR spectrum of Anomoian F (**9**) in MeOD (500 MHz)

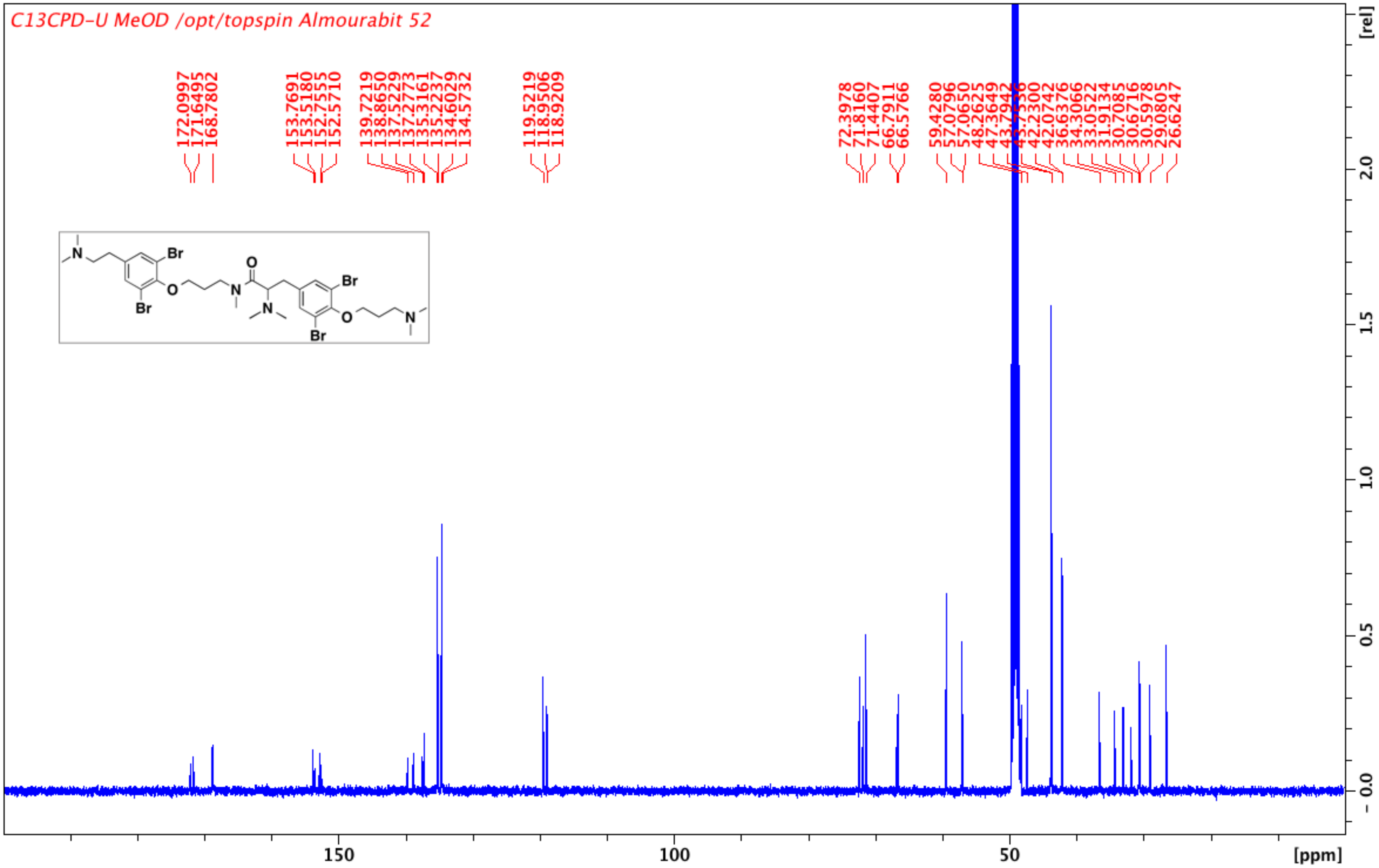

Figure S42. HSQC NMR spectrum of Anomoian F (**9**) in MeOD (500 MHz)

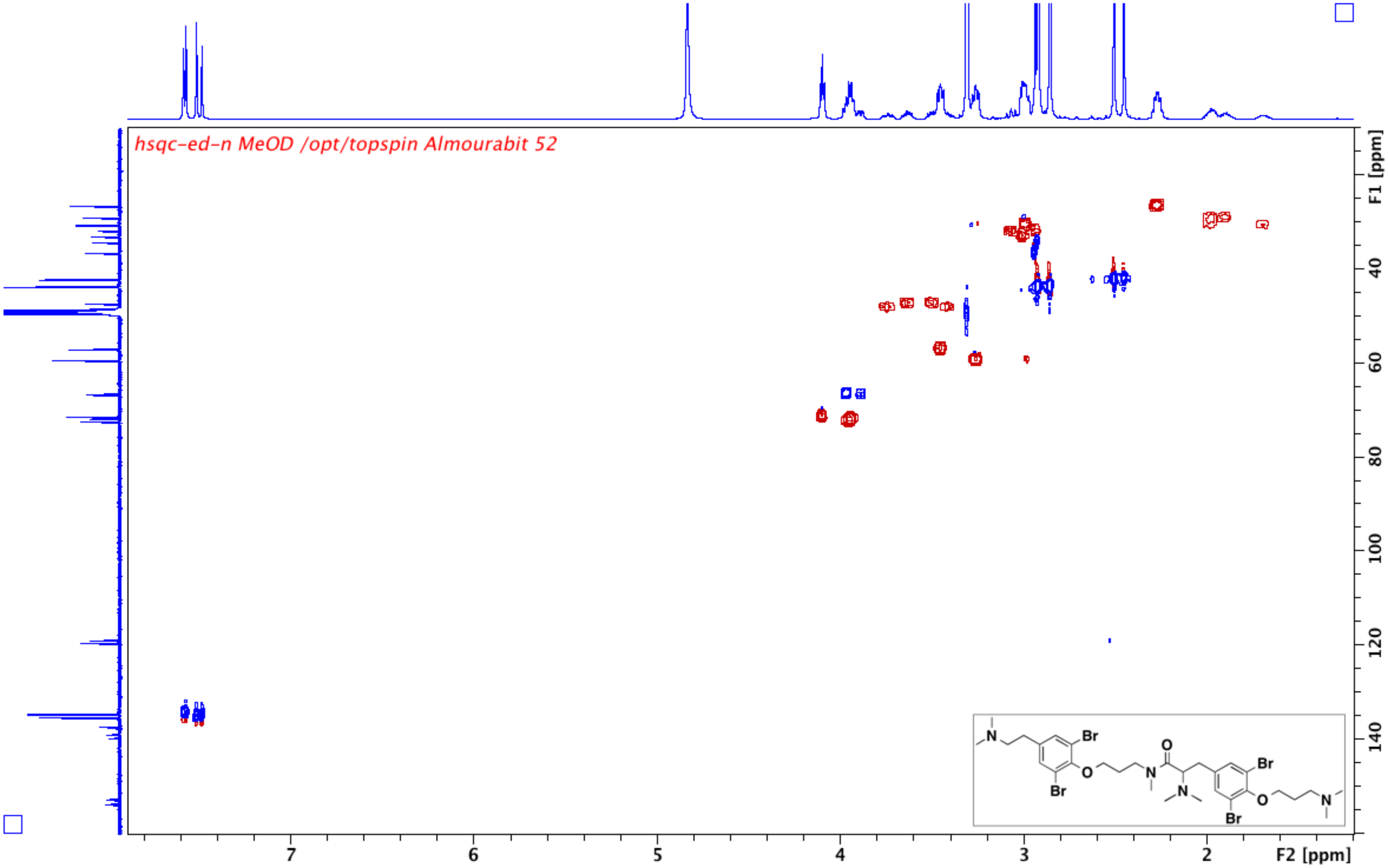

Figure S43. ^{1}H–^{1}H COSY NMR spectrum of Anomoian F (**9**) in MeOD (500 MHz)

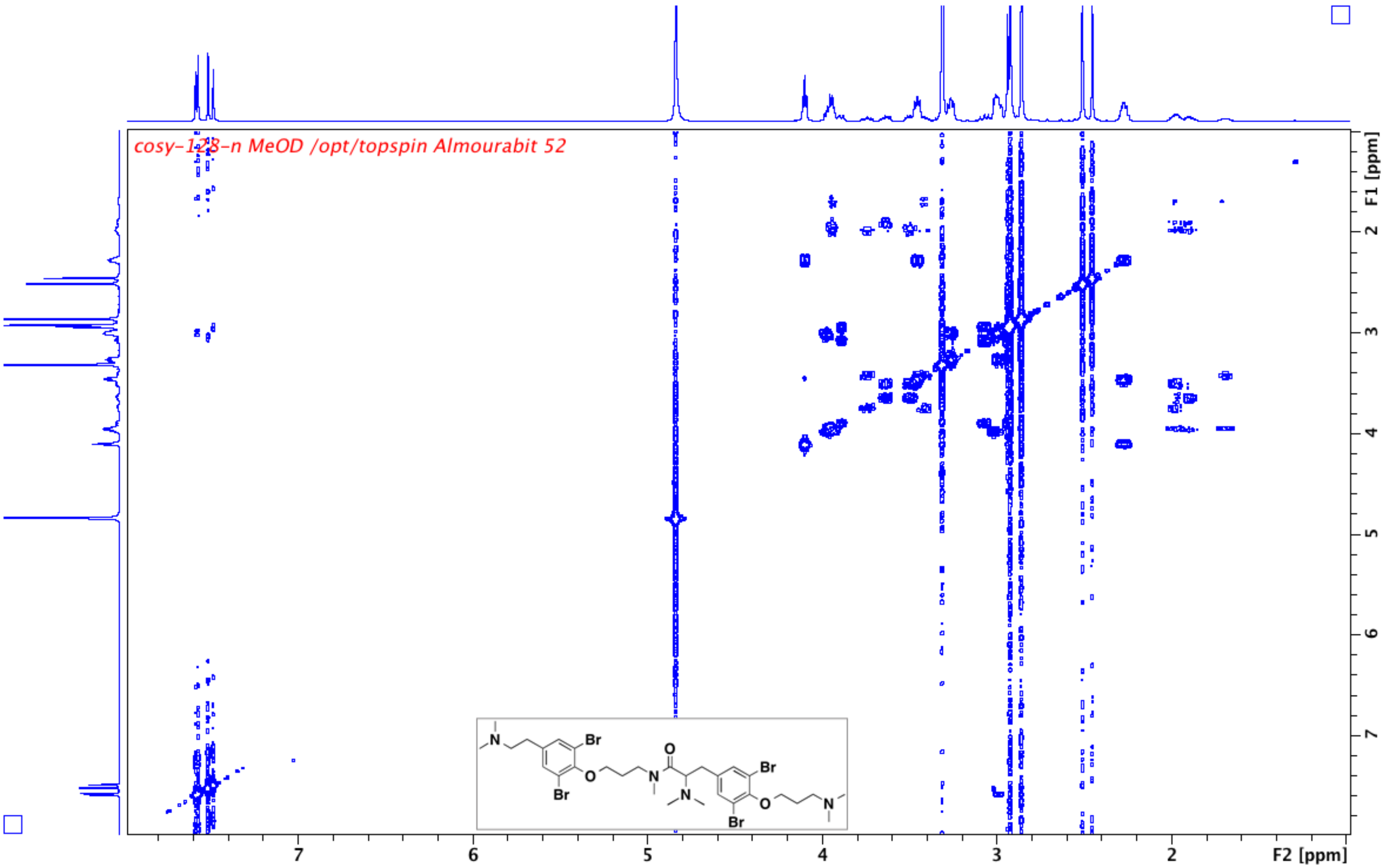

Figure S44. ^{1}H-^{13}C HMBC NMR spectrum of Anomoian F (**9**) in MeOD (500 MHz)

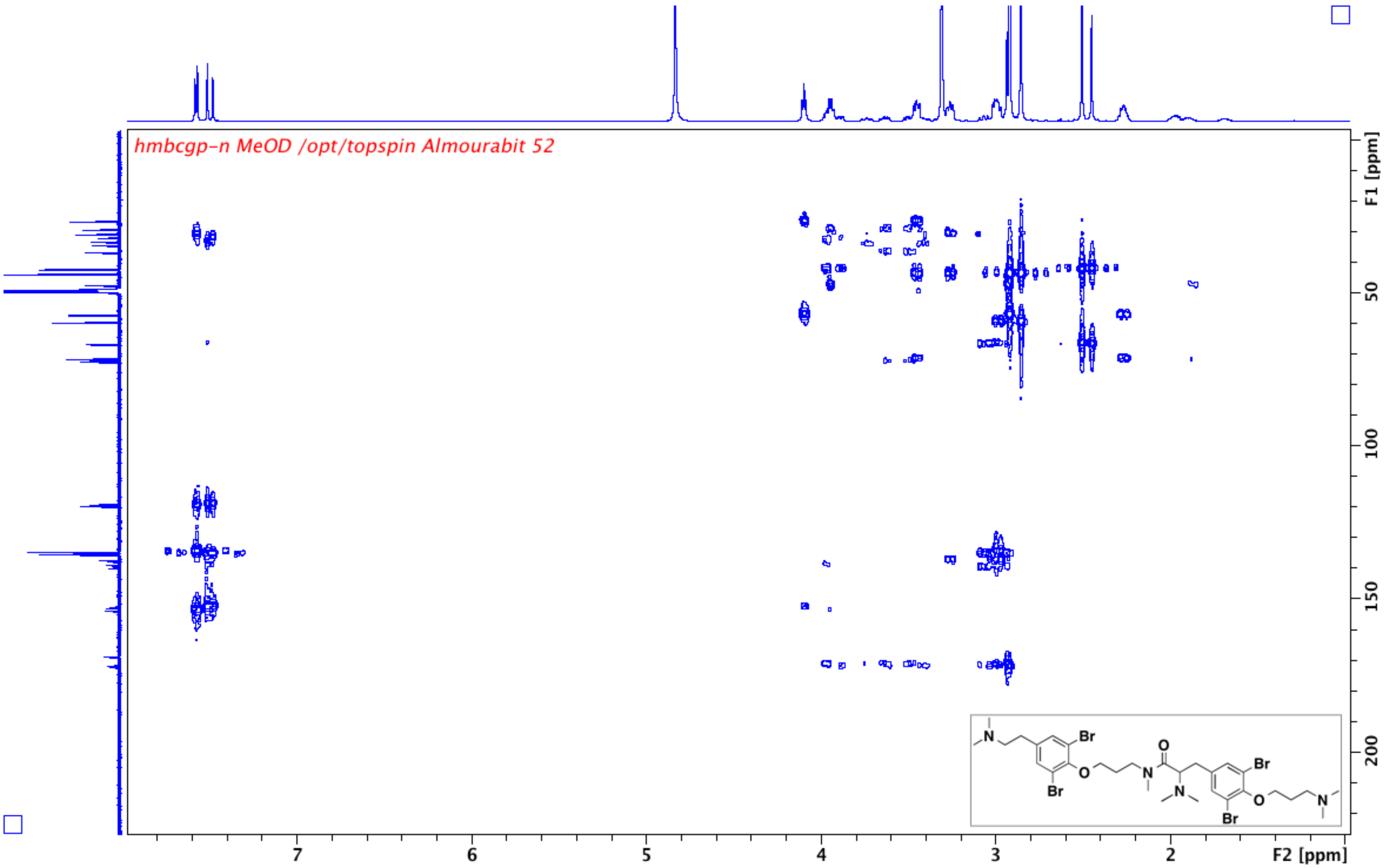

Figure S45. HR-ESI mass spectrum of Anomoian F (**9**)

Elemental Composition Report

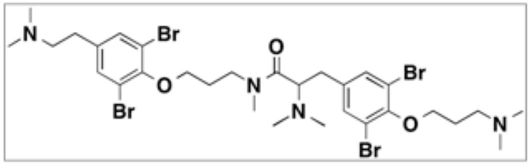

Single Mass Analysis
Tolerance = 10.0 PPM / DBE: min = -1.5, max = 100.0
Element prediction: Off
Number of isotope peaks used for i-FIT = 9

Monoisotopic Mass, Even Electron Ions
648 formula(e) evaluated with 9 results within limits (up to 50 best isotopic matches for each mass)
Elements Used:
C: 0-50 H: 0-100 N: 0-10 O: 0-10 79Br: 2-2 81Br: 2-2

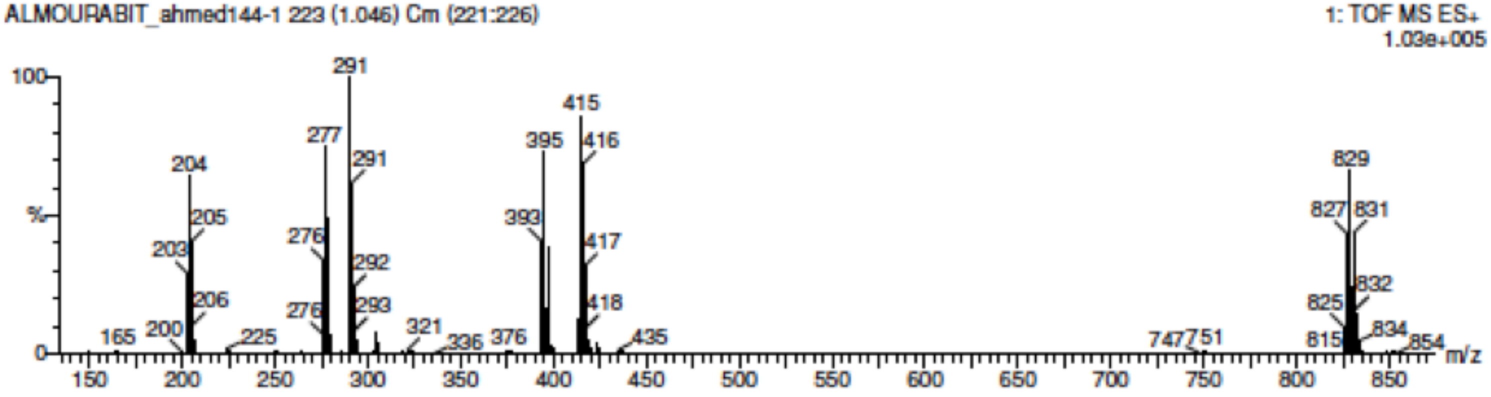

Minimum:				-1.5			
Maximum:		200.0	10.0	100.0			
Mass	Calc. Mass	mDa	PPM	DBE	i-FIT	i-FIT (Norm)	Formula
829.0259	829.0283	-2.4	-2.9	3.5	113.8	2.1	C28 H49 N2 O6 79Br2 81Br2
	829.0184	7.5	9.0	8.5	113.8	2.2	C30 H45 N4 O3 79Br2 81Br2
	829.0323	-6.4	-7.7	7.5	113.8	2.2	C33 H49 O4 79Br2 81Br2

Figure S46. ^{1}H NMR spectrum of N, N-dimethyldibromotyramine (**10**) in MeOD (500 MHz)

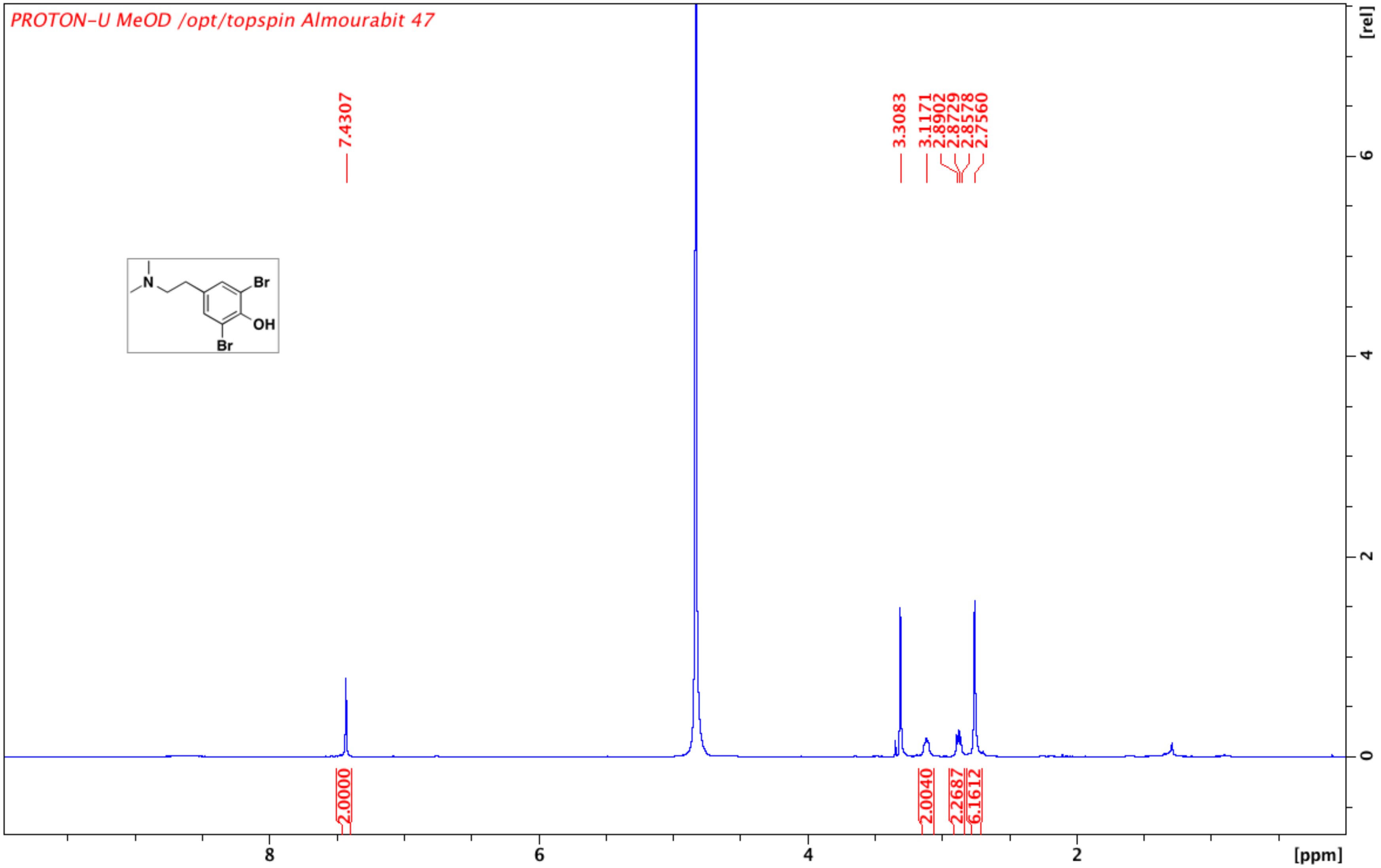

Figure S47. ^{13}C NMR spectrum of N, N-dimethyldibromotyramine (**10**) in MeOD (500 MHz)

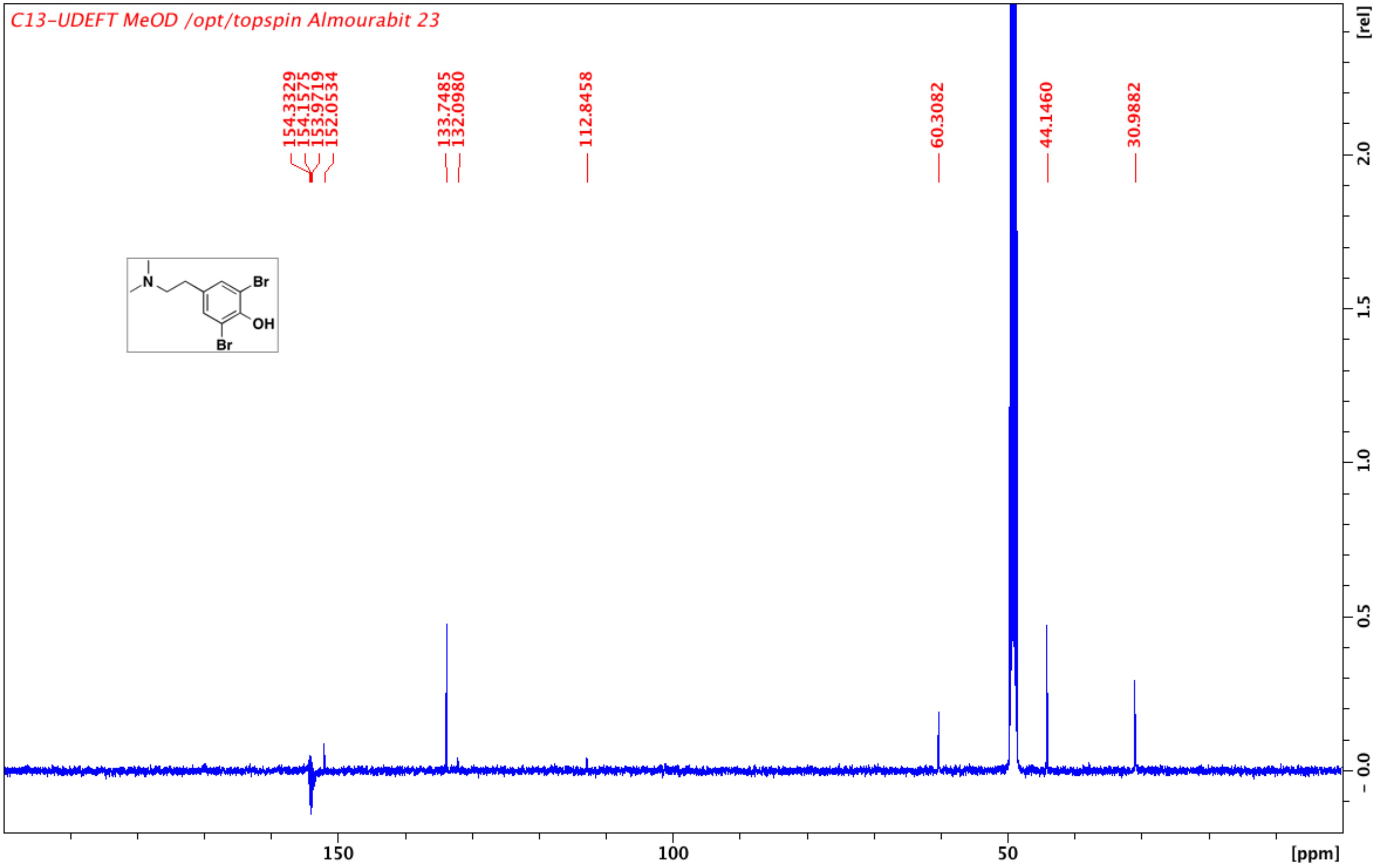

Figure S48. HR-ESI mass spectrum of N, N-dimethyldibromotyramine (**10**)

Elemental Composition Report

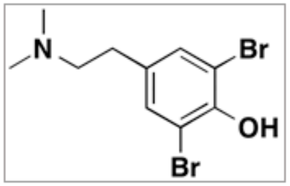

Single Mass Analysis

Tolerance = 20.0 PPM / DBE: min = -1.5, max = 100.0
Element prediction: Off
Number of isotope peaks used for i-FIT = 9

Monoisotopic Mass, Even Electron Ions
122 formula(e) evaluated with 3 results within limits (up to 50 best isotopic matches for each mass)
Elements Used:
C: 0-100 H: 0-120 N: 0-10 O: 0-10 79Br: 1-1 81Br: 1-1

ALMOURABIT_ahmed130-1 225 (1.054) Cm (220:239)

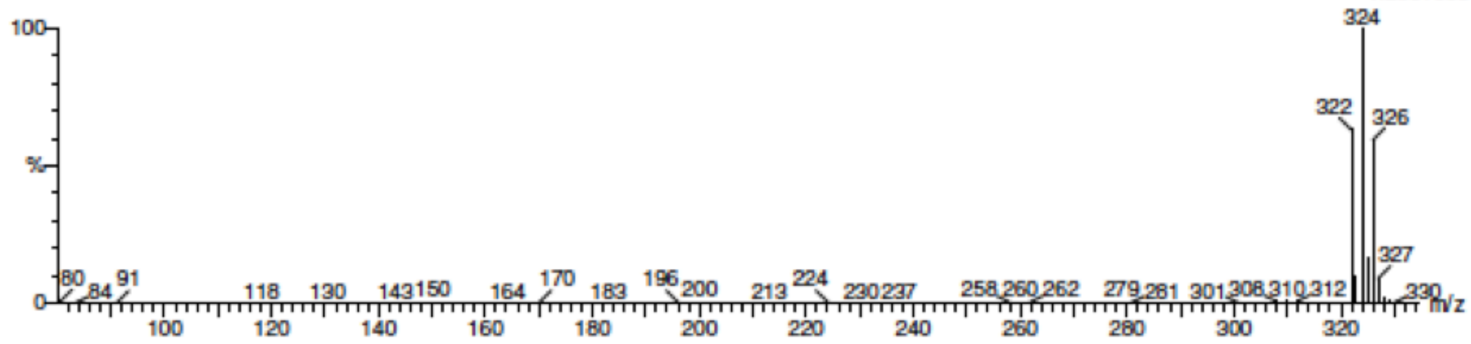

Minimum:				-1.5			
Maximum:		200.0	20.0	100.0			
Mass	Calc. Mass	mDa	PPM	DBE	i-FIT	i-FIT (Norm)	Formula
323.9388	323.9355	3.3	10.2	0.5	152.0	0.4	C H10 N9 O 79Br 81Br
	323.9381	0.7	2.2	-0.5	152.7	1.1	C5 H14 N3 O3 79Br 81Br
	323.9422	-3.4	-10.5	3.5	161.7	10.1	C10 H14 N O 79Br 81Br

Figure S49. ^{1}H NMR spectrum of 4,5,8-trihydroxyquinoline-2-carboxylic acid (**11**) in MeOD (500 MHz)

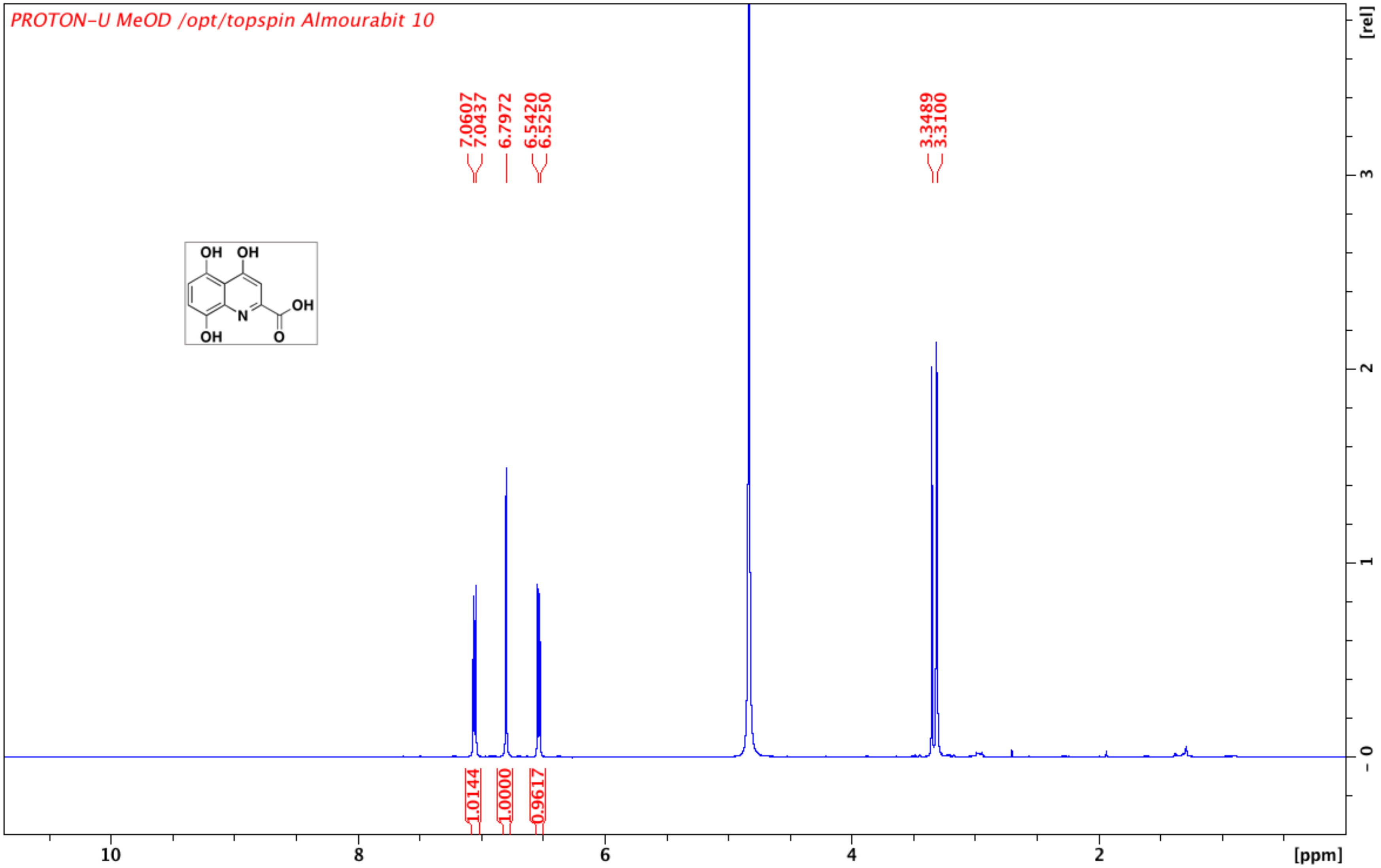

Figure S50. ^{13}C NMR spectrum of 4,5,8-trihydroxyquinoline-2-carboxylic acid (**11**) in MeOD (500 MHz)

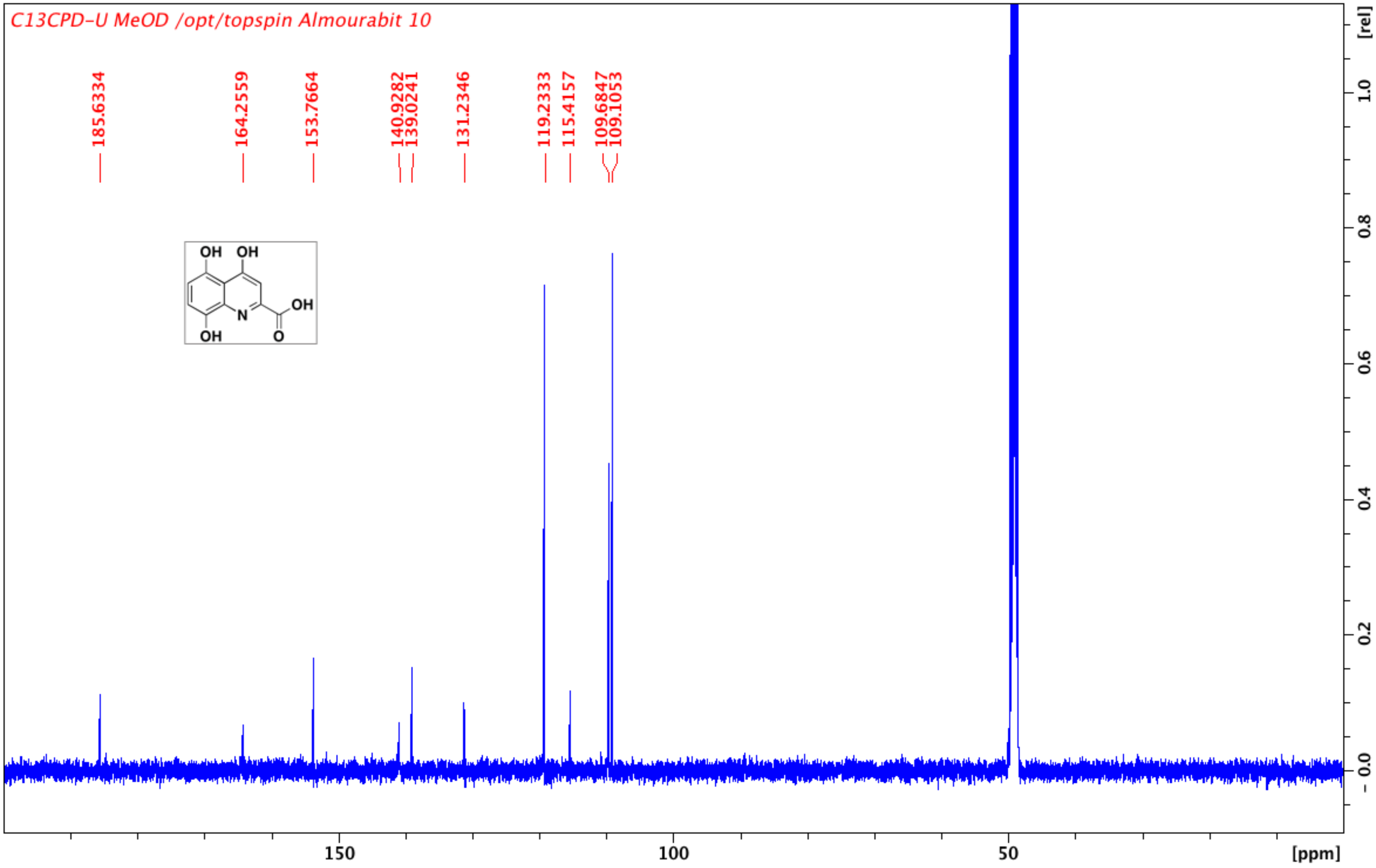

Figure S51. ^{1}H NMR spectrum of 4,5,8-trihydroxyquinoline-2-carboxylic acid (**11**) in DMF-d_7 (600 MHz)

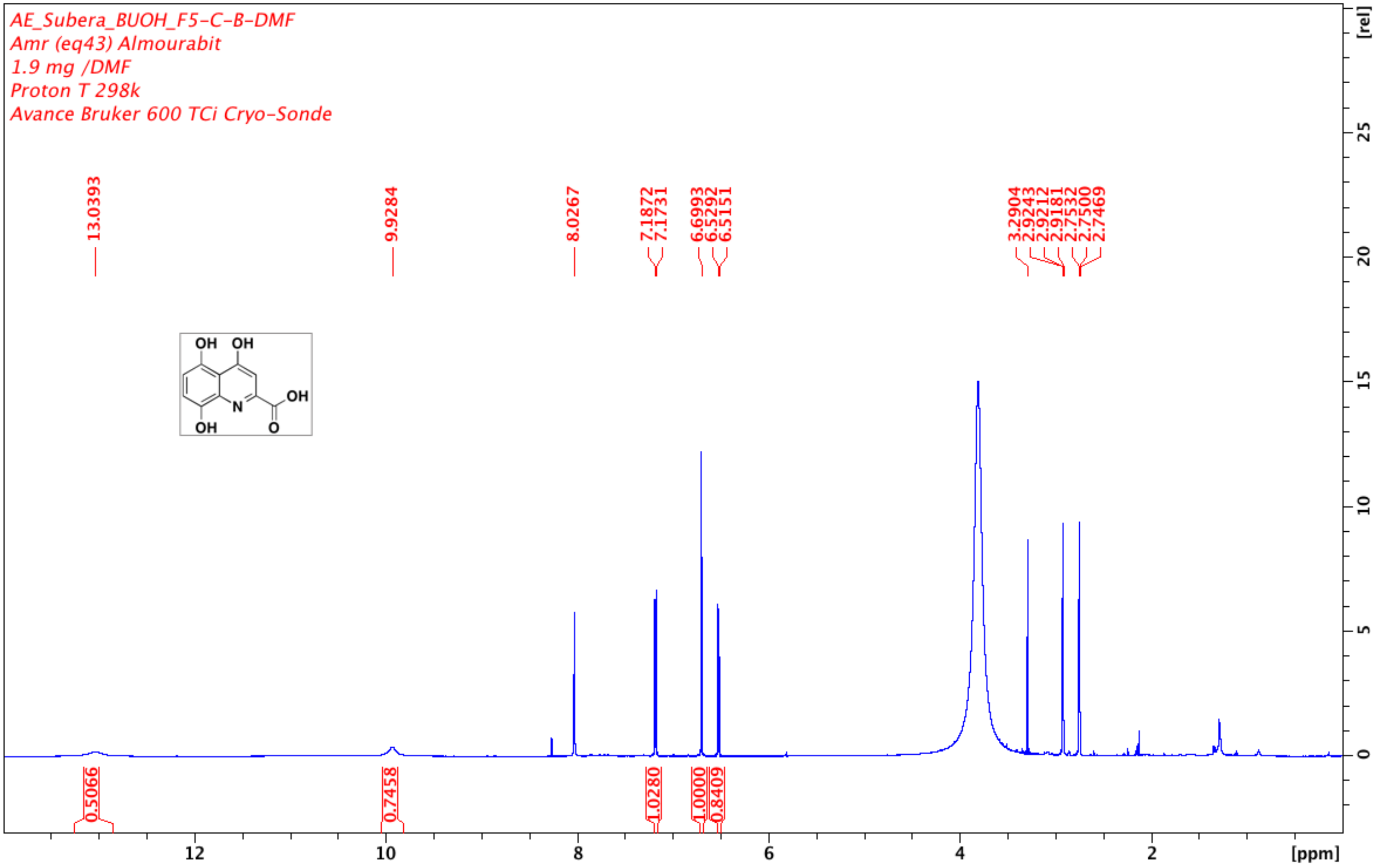

Figure S52. ^{1}H NMR spectrum of 4,5,8-trihydroxyquinoline-2-carboxylic acid (**11**) in DMSO (500 MHz)

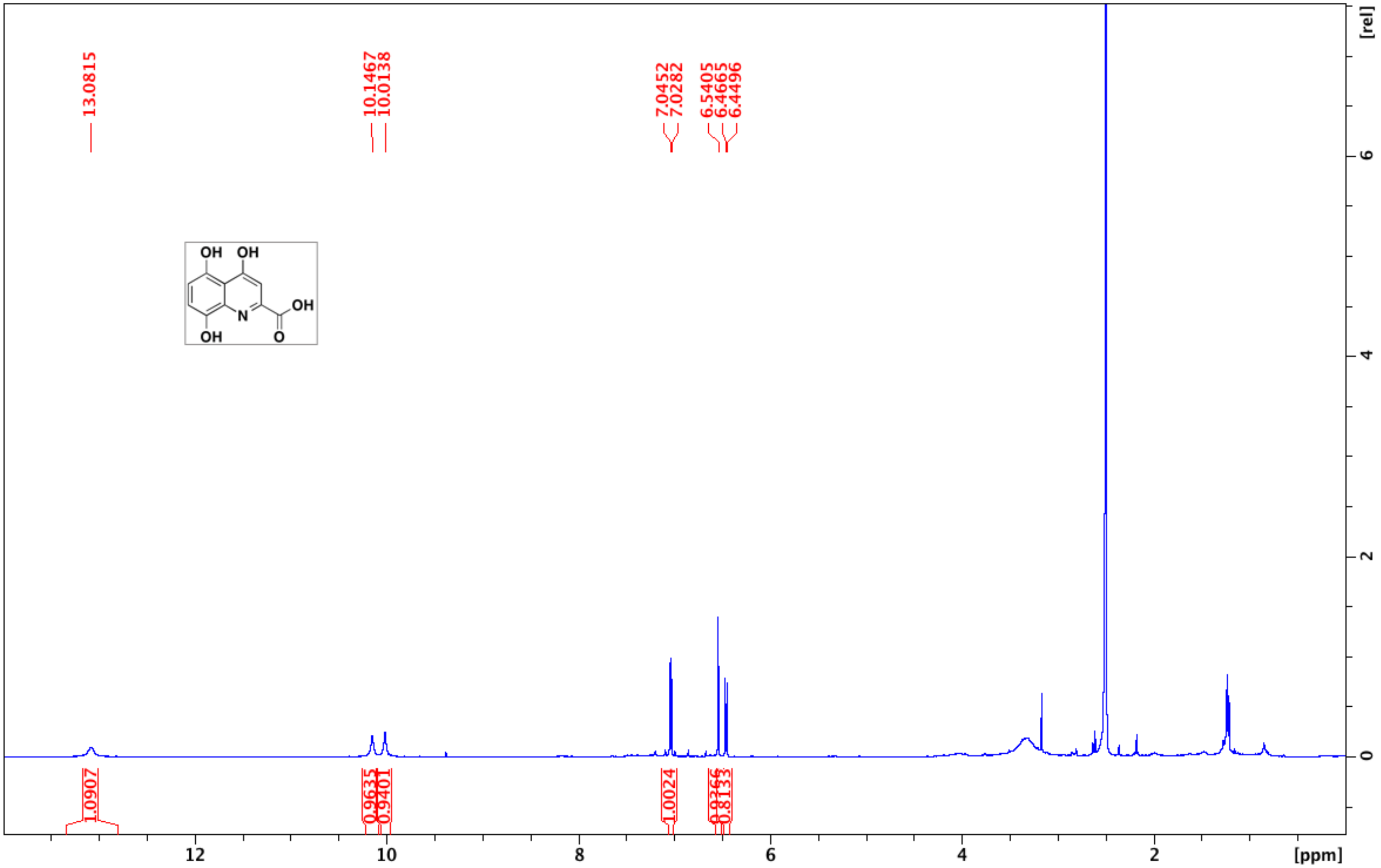

Figure S53. HR-ESI mass spectrum of 4,5,8-trihydroxyquinoline-2-carboxylic acid (**11**)

Elemental Composition Report

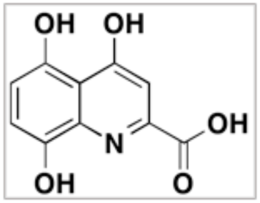

Single Mass Analysis

Tolerance = 50.0 PPM / DBE: min = -1.5, max = 100.0
Element prediction: Off
Number of isotope peaks used for i-FIT = 9

Monoisotopic Mass, Even Electron Ions
243 formula(e) evaluated with 11 results within limits (up to 50 best isotopic matches for each mass)
Elements Used:
C: 0-100 H: 0-100 N: 0-10 O: 0-10

ALMOURABIT_ahmed140-1 199 (0.948) Cm (195:204)

1: TOF MS ES+
2.38e+005

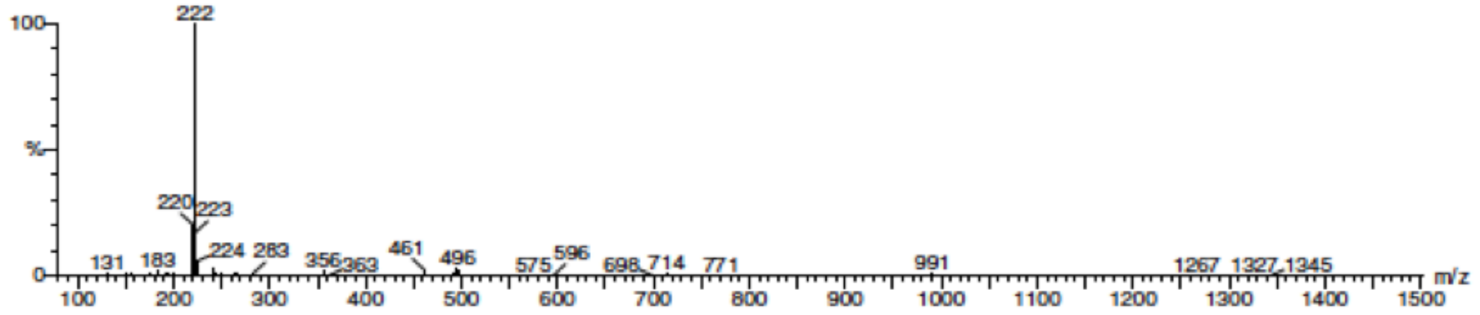

Minimum:				-1.5			
Maximum:		200.0	50.0	100.0			

Mass	Calc. Mass	mDa	PPM	DBE	i-FIT	i-FIT (Norm)	Formula
222.0441	222.0528	-8.7	-39.2	12.5	110.3	0.1	C10 H4 N7
	222.0488	-4.7	-21.2	8.5	112.9	2.7	C5 H4 N9 O2
	222.0416	2.5	11.3	12.5	113.3	3.2	C11 H4 N5 O
	222.0475	-3.4	-15.3	3.5	116.2	6.0	C4 H8 N5 O6
	222.0461	-2.0	-9.0	-1.5	116.7	6.6	C3 H12 N O10
	222.0515	-7.4	-33.3	7.5	117.1	6.9	C9 H8 N3 O4
	222.0376	6.5	29.3	8.5	122.4	12.3	C6 H4 N7 O3
	222.0335	10.6	47.7	4.5	122.6	12.4	C H4 N9 O5
	222.0344	9.7	43.7	16.5	124.0	13.9	C17 H4 N
	222.0362	7.9	35.6	3.5	125.3	15.2	C5 H8 N3 O7
	222.0402	3.9	17.6	7.5	126.7	16.5	C10 H8 N O5

Figure S54. ^{1}H NMR spectrum of Xantherunic acid (**12**) in MeOD (600 MHz)

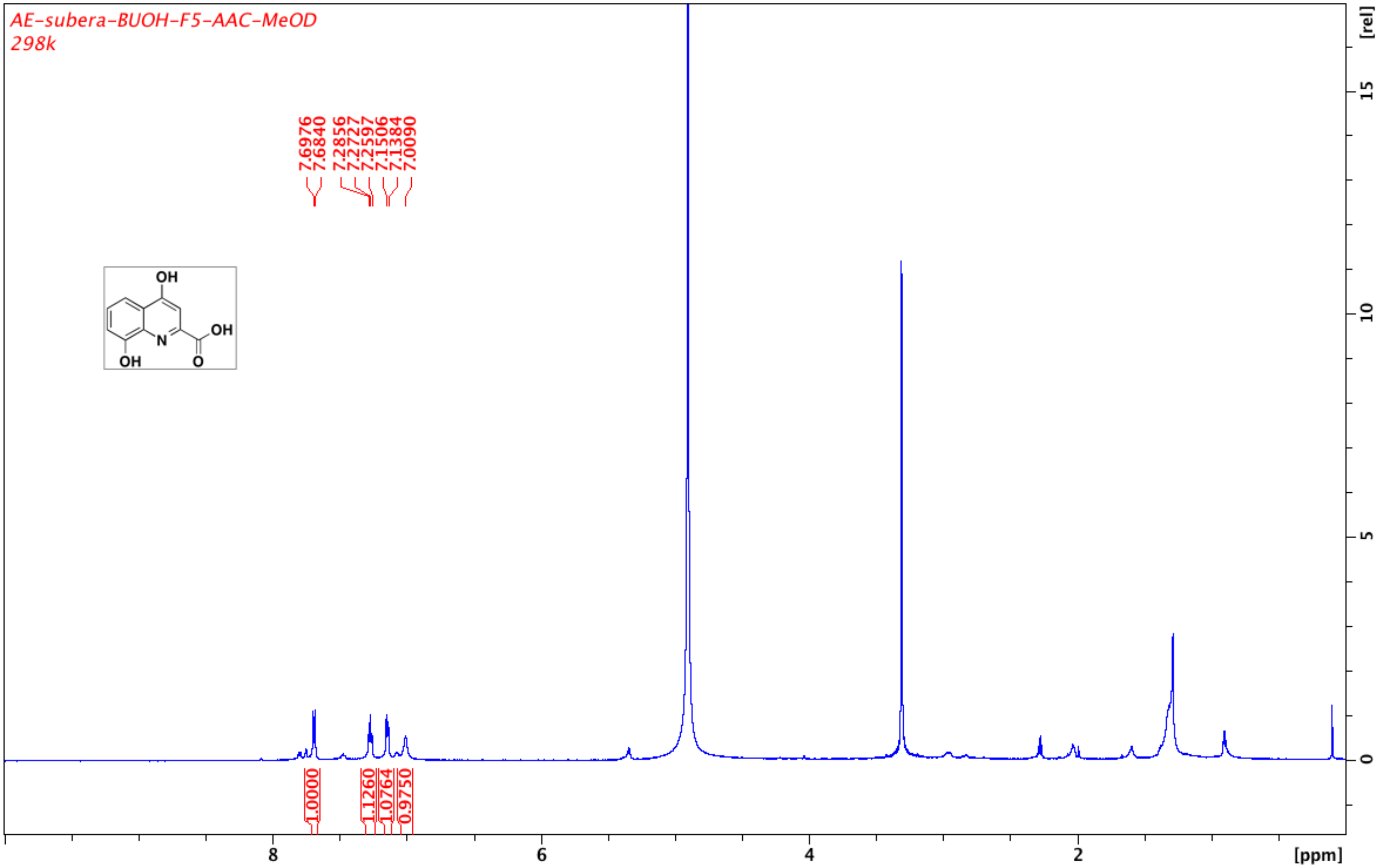

Figure S55. ^{13}C NMR spectrum of Xantherunic acid (**12**) in MeOD (600 MHz)

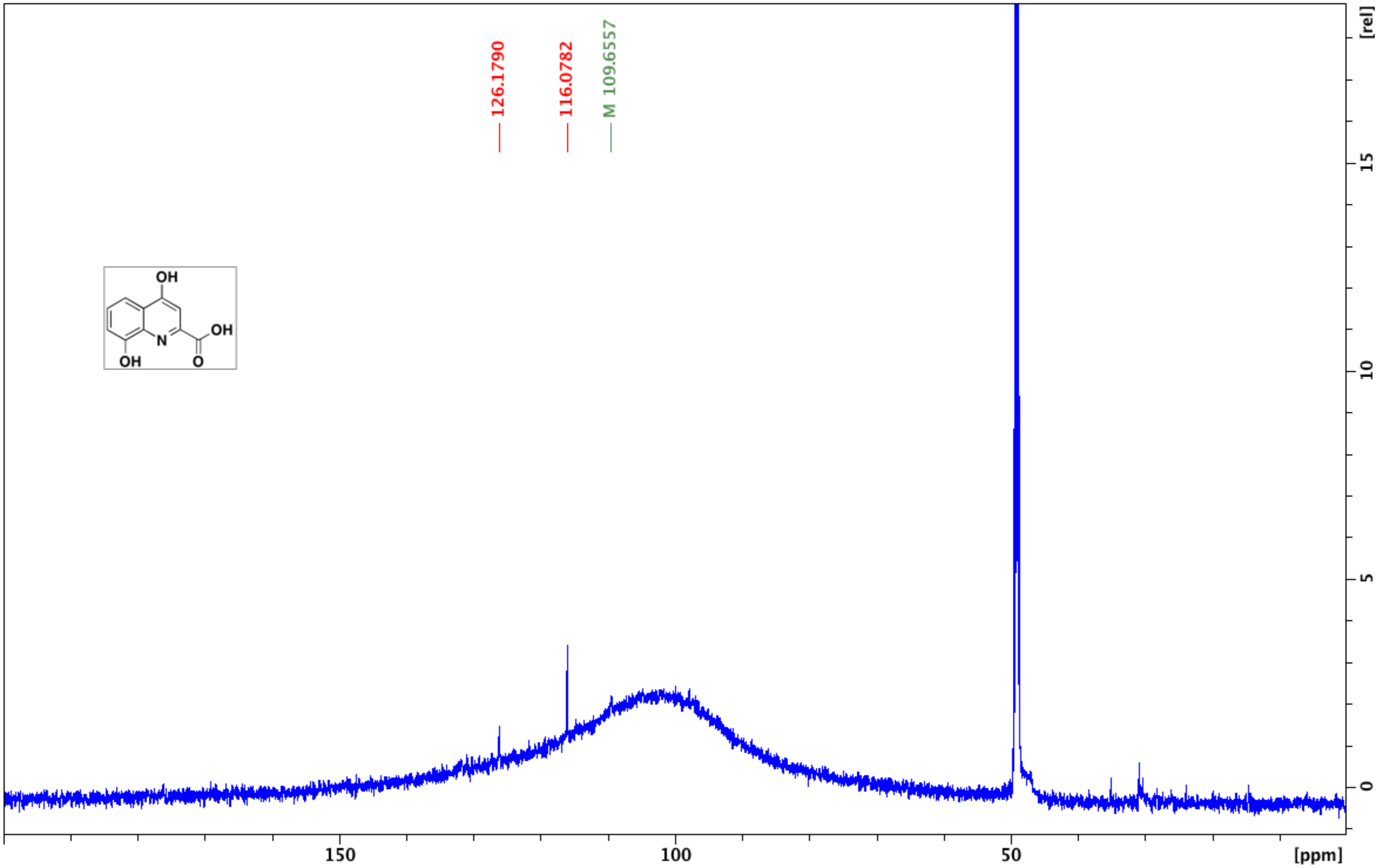

Figure S56. HR-ESI mass spectrum of Xantherunic acid (**12**)

Elemental Composition Report

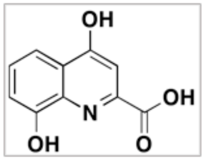

Single Mass Analysis

Tolerance = 30.0 PPM / DBE: min = -1.5, max = 100.0
Element prediction: Off
Number of isotope peaks used for i-FIT = 9

Monoisotopic Mass, Even Electron Ions
211 formula(e) evaluated with 6 results within limits (up to 50 best isotopic matches for each mass)
Elements Used:
C: 0-100 H: 0-100 N: 0-10 O: 0-10
ALMOURABIT_ahmed142-1 184 (0.879) Cm (182:188)

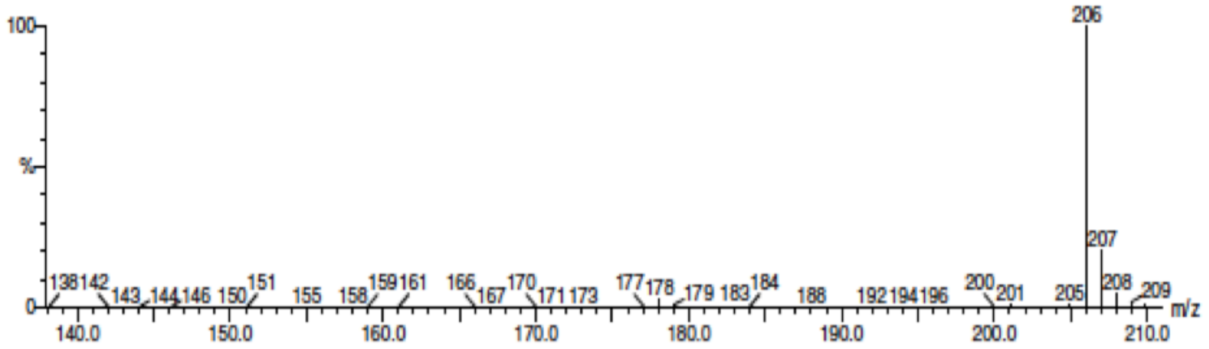

Minimum:				-1.5			
Maximum:		200.0	30.0	100.0			
Mass	Calc. Mass	mDa	PPM	DBE	i-FIT	i-FIT (Norm)	Formula
206.0511	206.0467	4.4	21.4	12.5	105.5	0.7	C11 H4 N5
	206.0453	5.8	28.1	7.5	106.1	1.3	C10 H8 N O4
	206.0566	-5.5	-26.7	7.5	106.6	1.8	C9 H8 N3 O3
	206.0539	-2.8	-13.6	8.5	108.5	3.6	C5 H4 N9 O
	206.0525	-1.4	-6.8	3.5	109.3	4.5	C4 H8 N5 O5
	206.0512	-0.1	-0.5	-1.5	110.6	5.8	C3 H12 N O9
